# Molecular Editing of Bilobalide: Regioselective C‑Ring Lactam Formation

**DOI:** 10.1021/acs.orglett.6c01262

**Published:** 2026-05-05

**Authors:** Wenjing Wang, Stephan Scheeff, Sam Chun-Kit Hau, Yao Qin, Chanin Sillapachaiyaporn, Billy Wai-Lung Ng

**Affiliations:** † School of Pharmacy, Faculty of Medicine, 26451 The Chinese University of Hong Kong, Hong Kong; ‡ Li Ka Shing Institute of Health Sciences, Faculty of Medicine, 26451 The Chinese University of Hong Kong, Hong Kong; § Department of Chemistry, Faculty of Science, 26451 The Chinese University of Hong Kong, Shatin, Hong Kong; ∥ CUHK-Hub of Obstetric and Paediatric Excellence, 26451 The Chinese University of Hong Kong, Hong Kong; ⊥ Gerald Choa Neuroscience Institute, 26451 The Chinese University of Hong Kong, Hong Kong; # Peter Hung Pain Research Institute, 26451 The Chinese University of Hong Kong, Hong Kong

## Abstract

Late-stage diversification of complex natural products enables rapid access to analogues for structure–activity relationship studies. Herein, we report a regioselective lactone-to-lactam editing strategy for bilobalide, affording previously inaccessible C-ring lactam analogues. The transformation proceeds through *in situ* activation of a benzoylated intermediate followed by cyclization, delivering fused bilobalide lactam scaffolds. This work expands the accessible chemical space of bilobalide for future biological exploration.

## Graphical abstract

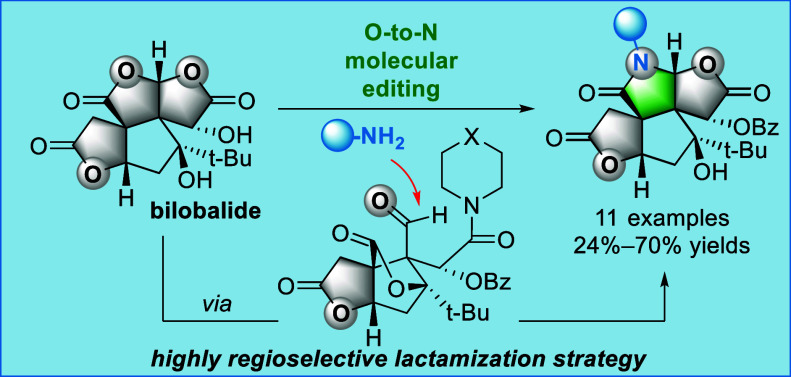

Late-stage derivatization of complex natural products is a valuable strategy in drug discovery. It enables rapid diversification of bioactive scaffolds for structure–activity relationship studies and pharmacological optimization.^1^ *Ginkgo biloba* is a prominent medicinal plant,^2,3^ whose leaves are rich in terpene trilactones, primarily represented by ginkgolides and bilobalide (Figure 1A).^4^ Its unique core scaffold and the high density of consecutive stereocenters have long attracted widespread attention as a target for total synthesis.^4–7^ In the late 1980s, Corey *et* *al*. accomplished the total synthesis of both racemic bilobalide^4^ and (−)-bilobalide^5^ and more recently, Shenvi *et* *al*. reported a concise asymmetric synthesis of bilobalide.^7^ Subsequent work from Shenvi *et* *al*. further interrogated bilobalide chemical space and highlighted the unusual reactivity of this densely functionalized scaffold.^8^

**Figure 1:**
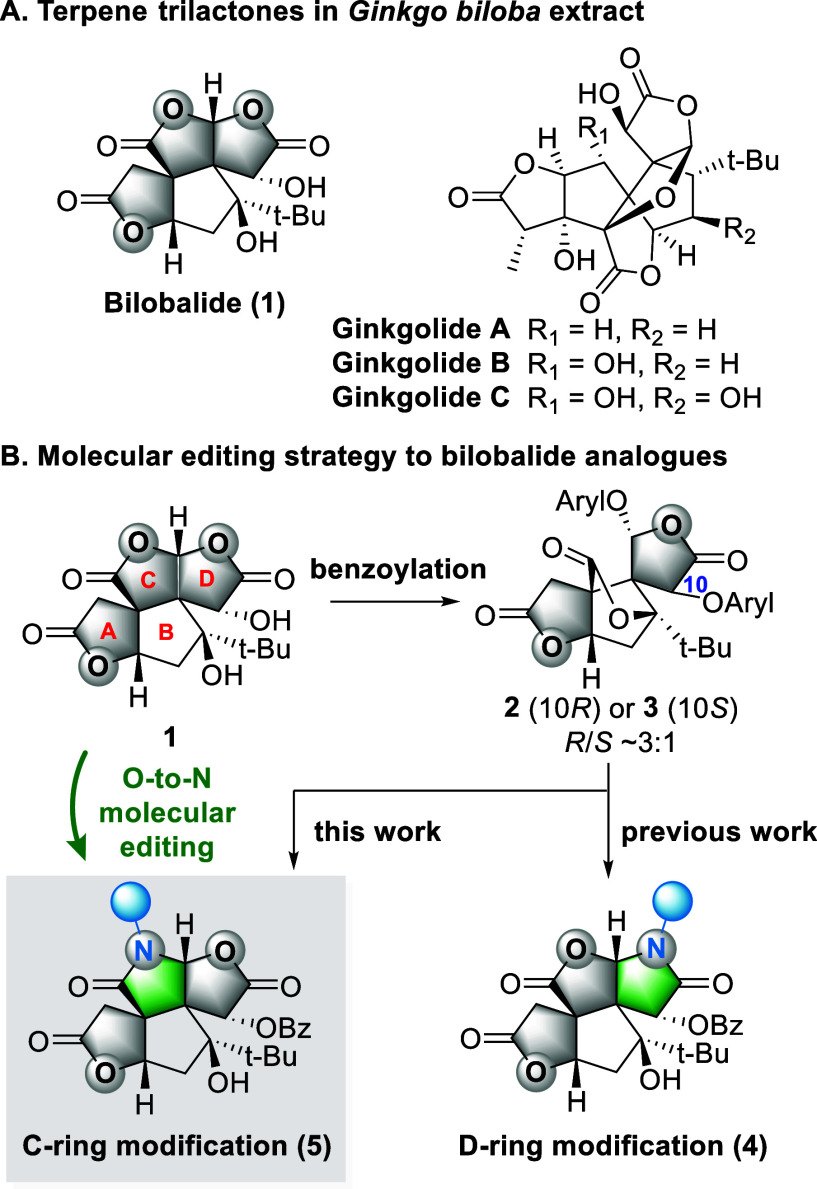
Background and concept of this work.

Although these studies provided insight into bilobalide reactivity, this knowledge has not yet translated into the design of biologically relevant analogues. Late-stage functionalization of bilobalide is rare and often unfruitful, as the base-sensitive, highly strained lactone framework impedes its use in medicinal chemistry studies.^3,9^ Previous strategies for the synthesis of bilobalide analogues resulted in the decay of the core structure and the subsequent alteration of the pharmacophore.^10^

Recently, we partially addressed this challenge by employing activated diaryl intermediates^10,11^ **2** and **3** to synthesize D-ring lactam analogues **4** (Figure 1B).^12^ Having established that bilobalide can undergo late-stage lactone-to-lactam editing without collapse of the core scaffold, we envisioned that this strategy could be redirected to the C-ring. Herein, we report a simple yet regioselective conversion, yielding a series of C-ring lactams **5**. In addition, we develop a practical TMEDA-mediated benzoylation protocol, which addresses existing limitations in the preparation of intermediate **2** and enables efficient, multigram-scale synthesis. Collectively, these advances provide a convergent strategy for previously inaccessible bilobalide derivatives.

Previous methods to obtain key intermediate **2a** or its epimer **3a** were hampered by the low reaction rate requiring harsh conditions to induce the rearrangement and benzoylation.^11^ Using reported conditions (BzCl, pyridine, 50 °C, 26 h), a mixture of C10 isomers was isolated in 73% total yield, which can only be separated after tedious column chromatography (Table 1). Consistent with our initial hypothesis that **3a** is the thermodynamically favored product of the enolized **2a**, pure **2a** was refluxed in a pyridine/toluene mixture, yielding a 1:1.8 mixture of **2a**/**3a** in 94% isolated recovery (Scheme 1). These results suggested that under milder conditions, the formation of **3a** may be suppressed.

**Scheme 1:**
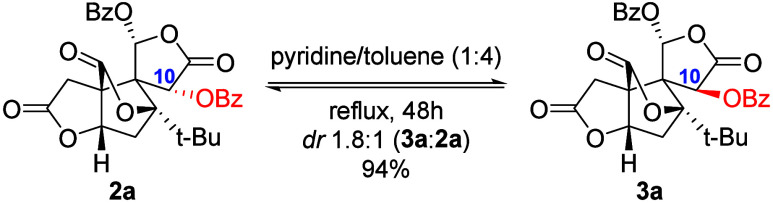
C10 Epimerization Induced by Pyridine

**Table 1:**
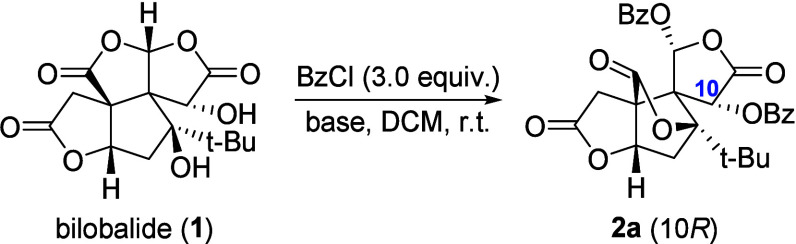
Conditions for Benzoylation

#	conditions	**2a** (%)
ref^11^	BzCl (15 equiv), pyridine, 50 °C, 26 h	55^a^
1	TMEDA (3.0 equiv), 3 h	98
2	TMEDA (0.2 equiv), DIPEA (2.8 equiv), 3 h	80
3	TMEDA (0.2 equiv), DIPEA (2.8 equiv), 18 h	82
4	DIPEA (3.0 equiv), 18 h	ND^b^

a Additionally, **3a** (10*S*) could be isolated in 18% yield.

b Not Detected.

Following this strategy, our first attempts to yield **2a** were disappointing. At room temperature, typical benzoylation protocols (e.g., pyridine/BzCl, DBU/BzCl, Et_3_N/BzCl, Et_3_N/Bz_2_O, HATU/BzOH/DIPEA) failed to afford detectable products. Because direct activation of bilobalide was not feasible, we turned to activation of the acylating reagent first. To our knowledge, only limited reports are available for the activation of reactive BzCl, one of these involves the use of TMEDA as a base to form a putative activated [BzTMEDA]^+^[Cl]^−^ complex.^13^ Gratifyingly, the use of TMEDA and BzCl gave **2a** in excellent yield under ambient conditions (Table 1, entry 1). The exceptional stereoretention (10*R*/10*S* > 20:1, as determined by ^1^H NMR analysis of the crude mixture) facilitated product isolation via simple workup and crystallization, enabling the multigram-scale access of **2a**. TMEDA was also effective in catalytic amounts when paired with DIPEA as a cobase, albeit with a marginal decrease in the yield of **2a** (Table 1, entries 2 and 3). Notably, no product formation was observed in the absence of TMEDA (Table 1, entry 4).

Encouraged by this result, we subsequently explored the scope of aromatic acyl chlorides (Scheme S1). The reaction of bilobalide with various aromatic acyl chlorides provided the corresponding analogues **2a** to **2i** in 63–98% yield. The transformation proceeded rapidly with excellent stereoretention at C10, underscoring the broad substrate scope and practical applicability of this method. Beyond **2a**, analogues **2b** to **2i** may also serve as useful entry points for further bilobalide diversification.

While we established the formation of D-ring lactams **4** from **2a**,^12^ we sought to develop strategies to selectively access the C-ring lactams **5**. We hypothesized that morpholine-derived aldehyde **6** could serve as a suitable substrate for this formation (Scheme 2). In detail, a primary amine should either cleave the lactone to form intermediate **I** or react with the aldehyde to yield hemiaminal **II**. Both pathways trigger a cascade reaction delivering desired compound **5**. Intermediate **II** presents a potential regioselectivity challenge, as **4** and **5** are available from **II**. In contrast, intermediate **I** would exclusively afford the desired C-ring lactam **5**.

**Scheme 2:**
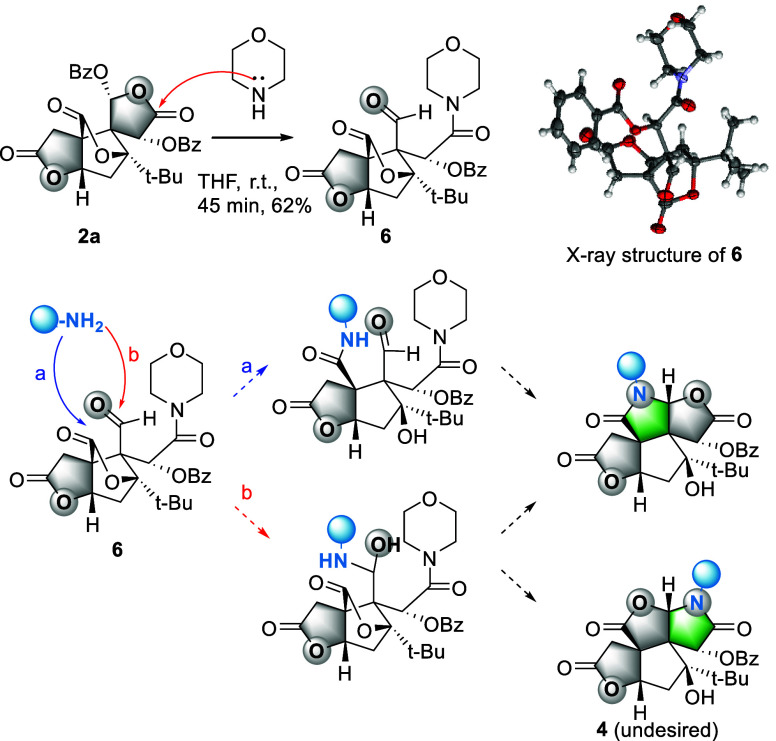
Proposed Synthesis of C-Ring Lactams from Aldehyde Intermediate **6**

To our delight, treatment of **6** with BnNH_2_ afforded a mixture of **4a** and **5a** (1:1.5 ratio) after 2 h (Figure S1). The formation of both regioisomers supports the involvement of intermediate **II** in this transformation. Notably, the formation of **6** from **2a** proceeded cleanly without the detection of any side products in the crude (see the Supporting Information). However, product **6** was only obtained in a modest yield following silica gel chromatography, suggesting degradation during isolation. Therefore, we developed a one-pot protocol to directly transform **2a** into **5a**, bypassing the isolation of intermediate **6**. Initially, solvent selection was found to influence both reactivity and regioselectivity between D-ring lactam **4a** and desired C-ring lactam **5a** (Table S1). While the *in situ* generation of **6** from **2a** with morpholine proceeds smoothly in ACN, DCM and THF, no conversion was observed in DMF. In the subsequent step to lactam **5a**, both regioselectivity and reactivity varied strongly. While the formation rates for **4a** and **5a** were highest in DCM or THF, DCM delivered the superior regioselectivity to **5a**. Therefore, DCM was identified as the optimal solvent for one-pot sequence, affording a 65% combined yield with a 2:1 regioselective ratio of **5a**:**4a** (Table 2, entry 1).

**Table 2:**
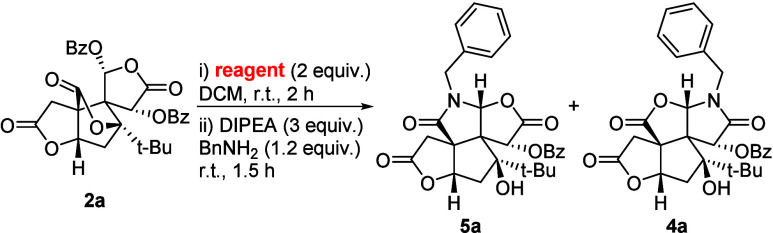
Conditions for C-Ring Modification

#	reagent	combined yield (%)	**5a**:**4a**
1	morpholine	65	2:1
2	piperazine	66	1.2:1
3	3,5-dimethyl morpholine	91	2.5:1
4	*trans*-2,5-dimethylpiperazine	60	<1:20
5	2,6-dimethyl piperazine	98	<1:20
6	1-Boc-piperazine	73	4.6:1

To further enhance the selectivity toward **5a**, the selection of the secondary amine reagent (e.g., morpholine) was critical. Amide formation to **6** needs to be reversible to allow the attack by the hemiaminal intermediate **II**. A sterically demanding reagent may favor the formation of **5a** through steric repulsion of the amide with the primary amine. However, it may also impede the formation of **6** (*vide infra*). Commonly employed amines with good leaving group properties (imidazole, DABCO, pyrrolidine) failed to yield any lactam product. Notably, only piperazine gave results comparable to those of morpholine, albeit with lower selectivity (Table 2, entry 2). Next, reagents with greater steric demand were investigated. The use of 3,5-dimethyl morpholine dramatically improved the yield while preserving the regioselectivity (entry 3). However, with sterically encumbered piperazine analogues (entries 4 and 5), **4a** was the sole isolated product. This result is attributed to the sluggish reaction of the hindered reagent with **2a**. Consequently, upon the addition of benzylamine in the subsequent step, **2a** reacts preferentially with BnNH_2_ to yield **4a** directly. Encouragingly, 1-Boc-piperazine afforded **5a** in good yield with improved selectivity (entry 6).

Accordingly, 1-Boc-piperazine and 3,5-dimethyl morpholine were selected as the most suitable reagents. We then explored whether reaction temperature in the second step affects the regioselectivity of C-ring modification. Interestingly, when the temperature was lowered to 0 °C, the C-ring/D-ring lactam ratio shifted significantly toward the undesired D-ring product **4a** (Table 3, entries 1 and 2). Meanwhile, we observed that a slight increase in benzylamine improved the yields while maintaining the overall regioselectivity (entries 3 and 4). Furthermore, elevated temperatures markedly enhanced selectivity, albeit at the expense of a reduced combined yield (entries 5 and 6). We concluded that conducting the reaction at room temperature was not only the most convenient approach but also offered the optimal balance between regioselectivity and combined yield (entry 7).

**Table 3:**
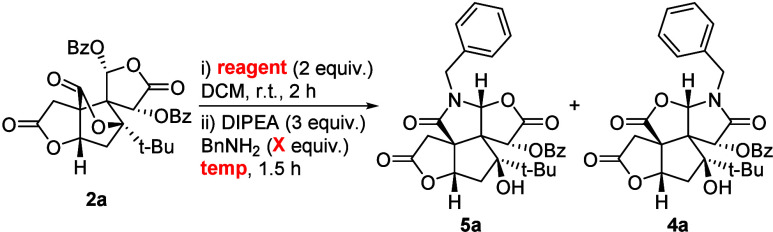
Optimization of Conditions for C-Ring Modification

#	reagent	temp (°C)	X equiv.	combined yield (%)	**5a**:**4a**
1	1-Boc-piperazine	0	1.2	78	1:1.2
2	3,5-dimethyl morpholine	0	1.2	76	1:1.7
3	1-Boc-piperazine	0	2.0	92	1.2:1
4	3,5-dimethyl morpholine	0	2.0	84	1:1.9
5	1-Boc-piperazine	40	2.0	70	6:1
6	3,5-dimethyl morpholine	40	2.0	67	3.5:1
7	1-Boc-piperazine	r.t.	2.0	81	4.8:1
8	1-Boc-piperazine; (step i, 3.5 h)	r.t.	2.0	70	>20:1

Of note, the strong temperature dependence of the **4a**:**5a** ratio suggests that the observed selectivity may be determined at the intermediate stage. However, we were unable to detect any interconversion between the isolated final products themselves. Consistent with our prior findings,^12^ both **4** and **5** are stable toward further modification; exposure to substituted amines, benzoylation conditions, or elevated temperatures did not result in any observable alteration.

Since residual intermediate **2a** was still detected after 2 h in the first-step reaction, a longer reaction time was employed to ensure the complete conversion of **2a** with 1-Boc-piperazine (entry 8). This change gave **5a** with excellent regioselectivity (>20:1).

Finally, we investigated the substrate scope of this transformation to prepare C-ring lactam analogues **5**. As shown in Scheme 3, a broad range of primary amines was compatible with this reaction condition. The relative configuration was determined through extensive 2D NMR studies of **5a** and unambiguously confirmed by X-ray crystallographic analysis of **5j**. Specifically, aryl amines afforded C-ring lactams **5b**, **5c**, **5i**−**5k** in moderate to good yields (44–70%). The isolation of compound **5h** was hindered by its high polarity. However, optimized chromatography conditions afforded enough material to evaluate its biological activity. For nonaromatic amines, the yields were generally lower, amounting to 34% for **5e** and **5f**.

**Scheme 3:**
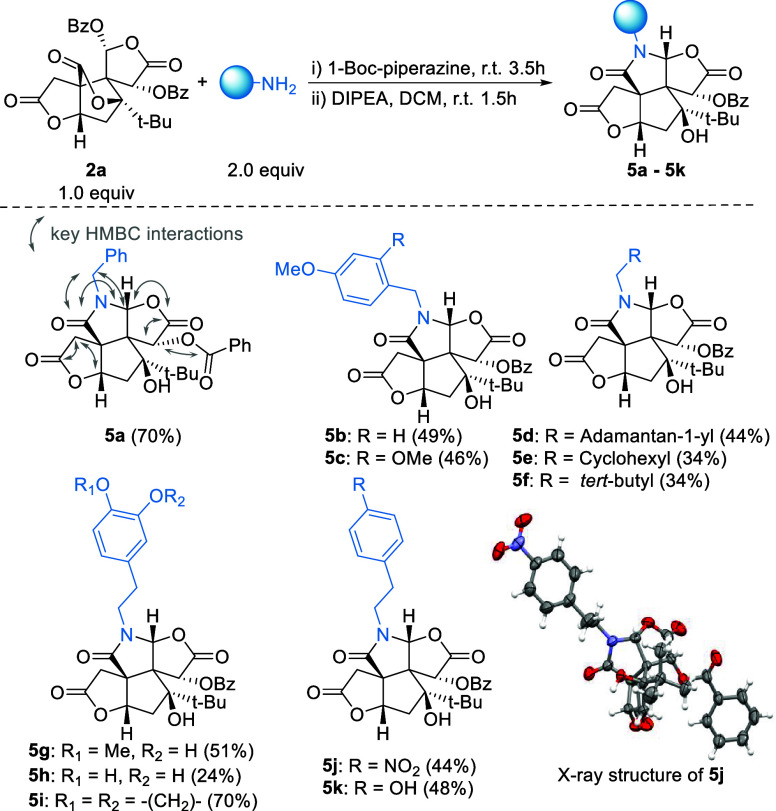
Regioselective Lactamization of **2a** with Various Amines^a^

a Yields denote isolated yields.

This complementary approach expands the accessible chemical space of bilobalide and may facilitate future exploration of biological targets and structure–activity relationships. Based on our previous findings, **BB10** (**4h**, corresponding D-ring analogue of **5h**) is a ferroptosis inhibitor and among the D-ring modifications the most potent. Therefore, we were interested in whether C-ring modified **5h** displays similar activity to **BB10**. The ferroptosis rescue assay revealed similar antiferroptosis activity in a dose-dependent manner (Figure 2) and **5h** did not show toxicity (Figure S2). These results suggest that the synthesis of the C-ring modification may provide opportunities to explore potential ferroptosis inhibitors.^14^ The examination of the full biology profile of the novel compounds is underway and will be reported elsewhere.

**Figure 2:**
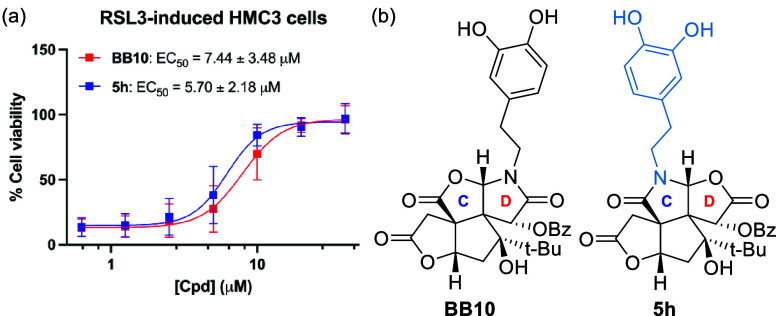
(a) Dose-dependent curves of **BB10** and **5h**. At 50% of maximal antiferroptosis effect, we indicate the EC_50_, which is the concentration of a compound that generates 50% of the maximal effect. HMC3 cells were pretreated with 200 nM RSL3 for 2 h, followed by the treatment of **BB10** or **5h** for 22 h. Cell viability was determined by CCK-8 assay. Data are plotted as mean ± s.d.; *n* = 3 biological replicates. (b) Chemical structure of **BB10** and **5h**.

In summary, we have identified a facile method to prepare a variety of C-ring modified bilobalide lactam analogues in moderate to good yields and high regioselectivity. This work has not only offered a new and useful strategy for the modification of bilobalide but also provided the opportunity to further study their biological applications.

## Supplementary Material



## Data Availability

The data underlying this study are available in the published article and its Supporting Information.

## Notes

Supporting figures, experimental details and methods, chemical synthesis and characterization data, NMR spectra (PDF) Deposition Numbers 2535289 (corresponds to X-ray structure of compound **5j**) and 2535290 (corresponds to X-ray structure of compound **6)** contain the supplementary crystallographic data for this paper (PDF)

## Notes

In memory of Professor Koji Nakanishi (1925–2019).
