# Supplementary material for: Molecular Editing of Bilobalide: Regioselective C‑Ring Lactam Formation
Source: Org Lett. 2026 May 5;28(19):6027–31. doi: 10.1021/acs.orglett.6c01262 (PMC13185091; doi:10.1021/acs.orglett.6c01262)

Supporting Information for

Molecular Editing of Bilobalide: Regioselective C-Ring Lactam Formation

Wenjing Wang[a, b⊥], Stephan Scheeff [a, b⊥], Sam Chun-Kit Hau[c], Yao Qin[a, b], Chanin Sillapachaiyaporn[a, b], Billy Wai-Lung Ng[a, b, d, e, f*]

[a] School of Pharmacy, Faculty of Medicine, The Chinese University of Hong Kong, Hong Kong

[b] Li Ka Shing Institute of Health Sciences, Faculty of Medicine, The Chinese University of Hong Kong, Hong Kong

[c] Department of Chemistry, Faculty of Science, The Chinese University of Hong Kong, Shatin, Hong Kong

[d] CUHK-Hub of Obstetric and Paediatric Excellence; The Chinese University of Hong Kong, Hong Kong

[e] Gerald Choa Neuroscience Institute, The Chinese University of Hong Kong, Hong Kong

[f] Peter Hung Pain Research Institute, The Chinese University of Hong Kong, Hong Kong

⊥Authors contributed equally

*Corresponding author. Email: billyng@cuhk.edu.hk

Table of Contents

1. Additional Figures and Schemes S3

2. Chemical Experiments S6

2.1. General Chemical Experimental Information S6

2.2. Optimization of Reaction Conditions S7

2.2.1. Solvents Selection for C-Ring Modification S7

2.2.2. Reagents Screening for C-Ring Modification S9

2.2.3. Conditions Optimization for C-Ring Modification S10

2.2.4. ^{1}H NMR spectra of the crude product S15

3. Synthesis Procedures and Analytical Data S23

3.1. General Procedure D: Synthesis of Dibenzoylated Analogues 2a-2i..........S23

3.2. General Procedure E: Synthesis of C-Ring modified 5a-5k S29

4. Biological Experiments S38

4.1. Cell Culture S38

4.2. Cell viability assay S38

5. X-Ray Data for Compounds 5j and 6 ....S39

6. NMR Spectra of final products S46

6.1. NMR spectra of diBz-Bilobalide analogues 2a-2i S46

6.2. NMR spectra of C-Ring Modification 5a-5k S54

1. Additional Figures and Schemes

Scheme S1. Dibenzoylation of bilobalide (1) with aromatic acyl chlorides

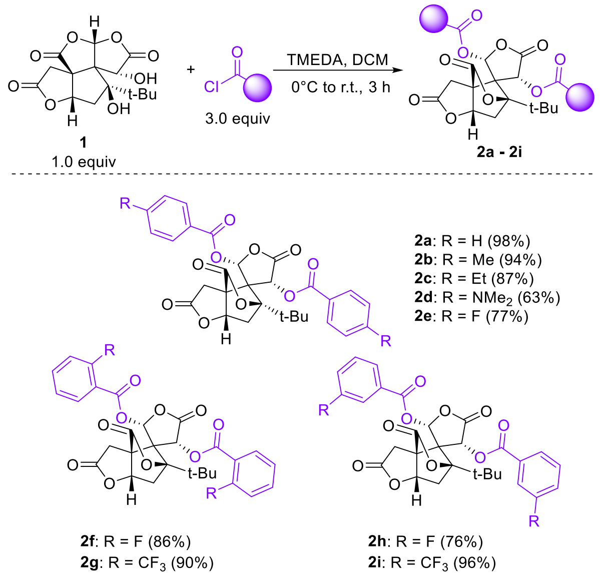

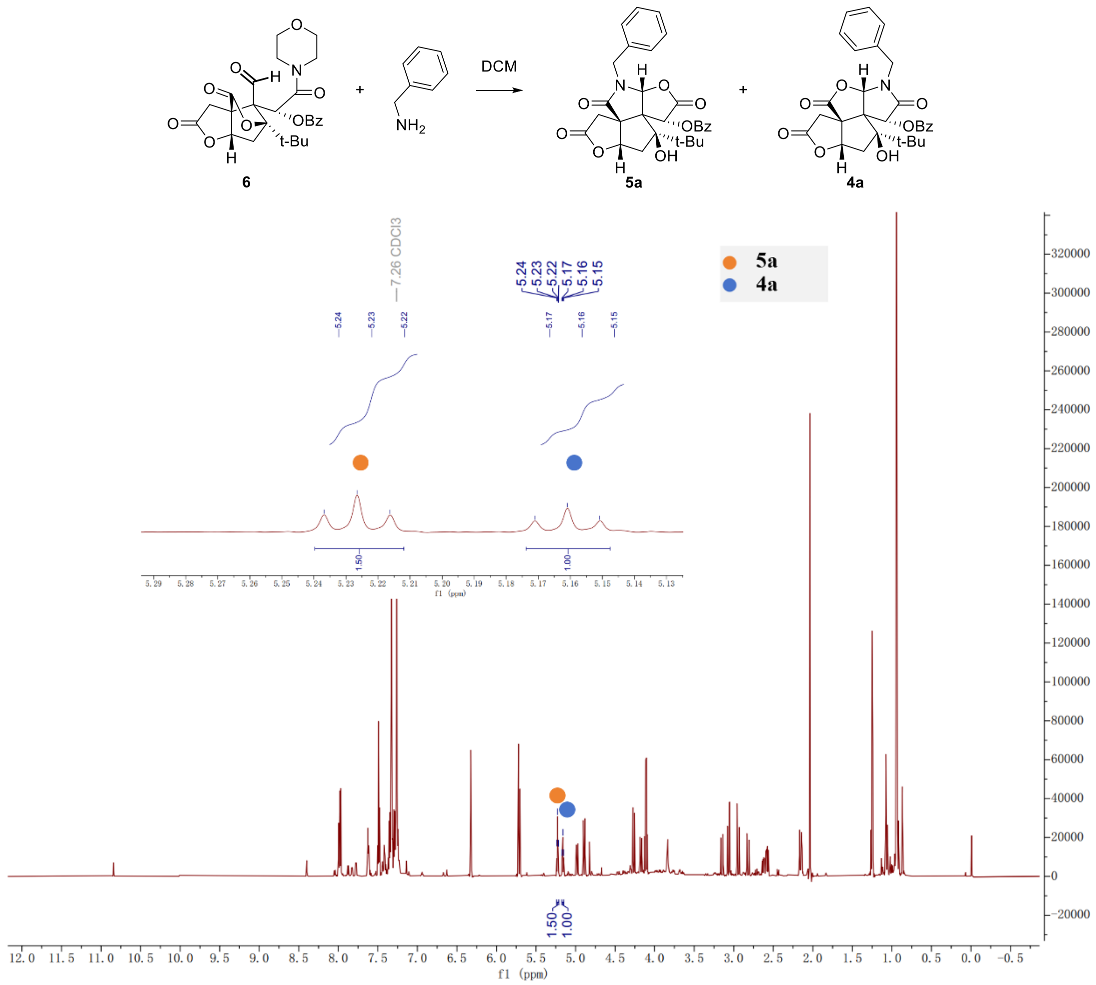

Figure S1. Intermediate **6** reacts with benzylamine to produce **4a** and **5a.**

Table S1. Solvents Selection for C-Ring Modification[a]

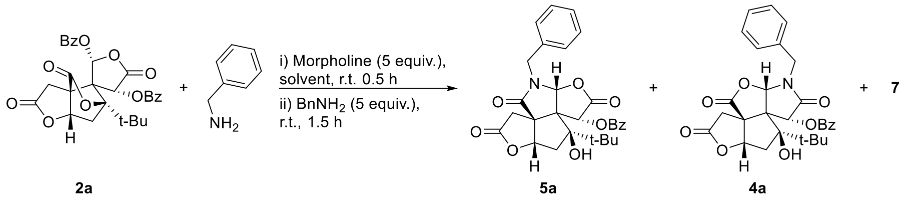

Compound **7** may be one of following structures

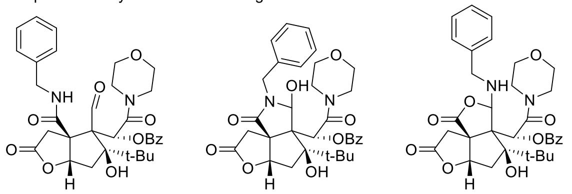

Entry	Solvent	Combined Yield	**5a**: **7**	**5a**: **4a**
1	DMF	-	-	-
2	ACN	-	1: 1.5	1.3: 1
3	THF	50	2.5: 1	2: 1
4	DCM	71	2: 1	7: 1

a) Reaction conditions: **2a** (0.019 mmol), solvent (1 mL).

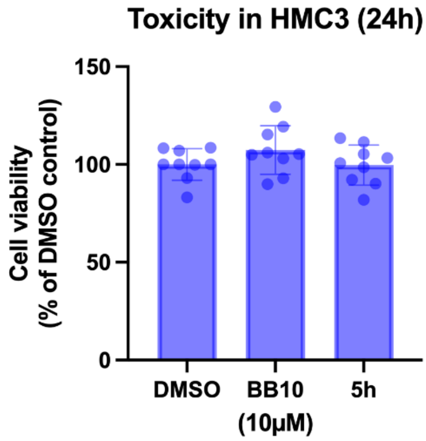

Figure S2. Toxicity evaluation of **BB10** and **5h** in HMC3 cells

2. Chemical Experiments

2.1. General Chemical Experimental Information

Unless otherwise specified, syntheses and manipulations of air- and moisture-sensitive materials were carried out under nitrogen atmosphere using standard Schlenk techniques. All glassware was oven-dried immediately prior to use. Authentic (–)-bilobalide (CAS: 33570-04-6) is commercially available and can be obtained from established chemical suppliers, including TCI, Sigma-Aldrich, MedChemExpress, Fluorochem or Energy Chemicals. Other materials were purchased from commercial sources and used without additional purification unless otherwise noted. Reactions were magnetically stirred and monitored by analytical thin-layer chromatography (TLC). TLC was performed on Merck silica gel 60 F_{254} TLC glass plates and observed by exposure to ultraviolet light. Organic solutions were concentrated by rotary evaporation at 20-45 °C.

Silica gel chromatography was performed on Chemical Reagent silica gel (200-300 mesh) using the indicated solvent system. The purity of the resulting compounds was determined by Agilent 6430 triple quadrupole liquid chromatography/tandem mass spectrometry (LC-MS/MS) (Agilent Technologies, Santa Clara, CA, United States). Nuclear magnetic resonance (NMR) spectra of ^{1}H and ^{13}C were obtained using a Bruker Ultrashield 400 Plus NMR spectrometer or Bruker Ascend 700 NMR spectrometer at ambient temperature. Chemical shifts (δ) were indicated in parts per million (ppm). Tetramethylsilane was used as an internal reference. Coupling constants, *J*, were expressed in Hertz. The following abbreviations are used for signal multiplicities: singlet, s; doublet, d; triplet, t; quartet, q; multiplet, m; broad, br. Samples were tested using flow-injection analysis into a Thermo Q Exactive™ Focus Hybrid Quadrupole-Orbitrap™ Mass Spectrometer for high-resolution mass spectrometry (HRMS) analysis. X-ray crystallographic analysis was performed on Bruker D8 Venture Diffractometer. Crystal structural data were collected by the single-crystal X-ray diffraction method with a Bruker D8-Venture system. Structural assignments were made with additional information from gNOESY, gHSQC, and gHMBC experiments.

2.2. Optimization of Reaction Conditions

2.2.1. Solvents Selection for C-Ring Modification

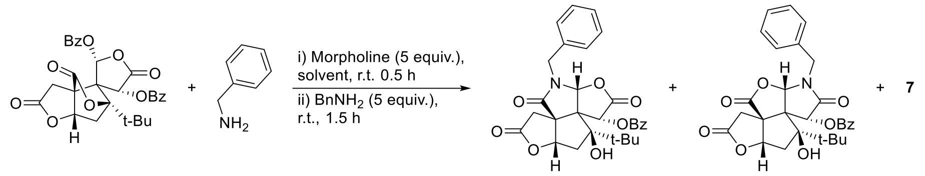

2a **5a** **4a**

General procedure A:

To an oven-dried round bottom flask with a magnetic stir bar was added **2a** (10 mg, 0.019 mmol, 1.0 equiv.), followed by the addition of solvent (2 mL). Morpholine was then added to the flask. The resulting solution was stirred at room temperature for 0.5 h. Upon completion indicated by TLC, benzylamine (10 µL, 0.095 mmol, 5.0 equiv.) was added. The reaction solution was stirred for 1.5 h. The solution was diluted with saturated NH_4Cl solution (10 mL) and DCM (10 mL). After phase separation, the aqueous phase was extracted with DCM (2 x 10 mL). Then the combined organic layers were washed with brine (20 mL), dried over Na_2SO_4, and concentrated *in vacuo*. The crude products were analyzed using LCMS. The regioselectivity of the compound can be determined by the analysis of the crude reaction mixture using ^{1}H NMR spectroscopy.

Entry 1: The reaction was performed on a 0.019 mmol scale following the **general procedure A.** Compound **2a** (10 mg, 0.019 mmol, 1.0 equiv.) was dissolved in Dimethylformamide (DMF, 1 mL). Morpholine (8 µL, 0.095 mmol, 5.0 equiv.) was then added to the flask. The resulting solution was stirred for 0.5 h at room temperature. Upon completion indicated by TLC, benzylamine (10 µL, 0.095 mmol, 5.0 equiv.) was added. The reaction solution was stirred for 1.5 h at room temperature. The result was checked by the analysis of the crude reaction using LCMS and no conversion was observed.

Entry 2: The reaction was performed on a 0.019 mmol scale following **general procedure A**. Compound **2a** (10 mg, 0.019 mmol, 1.0 equiv.) was dissolved in ACN (1

mL). Morpholine (8 μL, 0.095 mmol, 5.0 equiv.) was then added to the flask. The resulting solution was stirred for 0.5 h at room temperature. Upon completion indicated by TLC, benzylamine (10 μL, 0.095 mmol, 5.0 equiv.) was added. The reaction solution was stirred for 1.5 h at room temperature. The result was checked by the analysis of the crude reaction using LCMS. The ratio of **5a** to **4a** is 1.3: 1 and the ratio of **5a** to **7** is 1: 1.5, respectively, as determined by analyzing the crude reaction mixture using LCMS.

Entry 3: The reaction was performed on a 0.019 mmol scale following **general procedure A**. Compound **2a** (10 mg, 0.019 mmol, 1.0 equiv.) was dissolved in tetrahydrofuran (THF, 1 mL). Morpholine (8 μL, 0.095 mmol, 5.0 equiv.) was then added to the flask. The resulting solution was stirred for 0.5 h at room temperature. Upon completion indicated by TLC, benzylamine (10 μL, 0.095 mmol, 5.0 equiv.) was added to the reaction solution for 1.5 h at room temperature. The products were detected by the analysis of the crude reaction using LCMS. The combined yield of **4a**, **5a**, and **7** was 50%. The ratio of **5a** to **4a** is 2: 1 and the ratio of **5a** to **7** is 2.5: 1, respectively, as determined by analyzing the crude reaction mixture using LCMS.

Entry 4: The reaction was performed on a 0.019 mmol scale following **general procedure A**. Compound **2a** (10 mg, 0.019 mmol, 1.0 equiv.) was dissolved in DCM (2 mL). Morpholine (8 μL, 0.095 mmol, 5.0 equiv.) was then added. The resulting solution was stirred for 0.5 h at room temperature. Upon completion indicated by TLC, benzylamine (10 μL, 0.095 mmol, 5.0 equiv.) was added. The reaction solution was stirred for 1.5 h at room temperature. The products were detected by the analysis of the crude reaction using LCMS. The combined yield of **4a**, **5a**, and **7** was 71%. The ratio of **5a** to **4a** is 7: 1 and the ratio of **5a** to **7** is 2: 1, respectively, as determined by analyzing the crude reaction mixture using LCMS.

2.2.2. Reagents Screening for C-Ring Modification

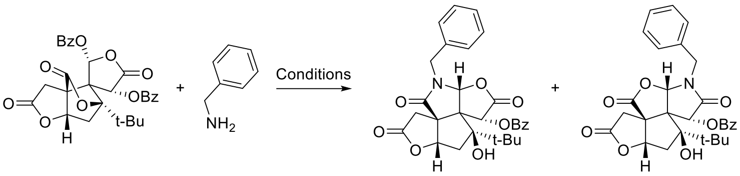

2a **5a** **4a**

General procedure B:

To an oven-dried round bottom flask with a magnetic stir bar was added **2a** (26.7 mg, 0.05 mmol, 1.0 equiv.) in the solvent (1 mL). Reagent was then added. The resulting solution was stirred. Upon completion as indicated by TLC, benzylamine was added to the reaction solution. With the reaction finished, the solution was diluted with saturated NH_4Cl solution (5 mL) and DCM (5 mL). After phase separation, the aqueous phase was extracted with DCM (2 x 5 mL). Then the combined organic layers were washed with brine (10 mL), dried over Na_2SO_4, and concentrated *in vacuo*. The crude was analyzed using LCMS. The respective yield can be determined by the analysis of the crude reaction mixture using ^{1}H NMR spectroscopy. The crude product was purified by column chromatography using hexane/EtOAc to provide **5a**.

Entry 1: The reaction was performed on a 0.05 mmol scale following **general procedure B**. Compound **2a** (26.7 mg, 0.05 mmol, 1.0 equiv.) was dissolved in dry THF (1 mL). Imidazole (8.5 mg, 0.13 mmol, 2.5 equiv.) was then added to the flask. The resulting solution was stirred at room temperature. The result was checked by the analysis of the crude reaction using LCMS and no conversion was observed.

Entry 2: The reaction was performed on a 0.05 mmol scale following **general procedure B**. Compound **2a** (26.7 mg, 0.05 mmol, 1.0 equiv.) was dissolved in THF (1 mL). 1,4-diazabicyclo[2.2.2]octane (DABCO, 14 mg, 0.13 mmol, 2.5 equiv.) was then added to the flask. The resulting solution was stirred at room temperature. The result was checked by the analysis of the crude reaction using LCMS and it shows that no or slow conversion was performed.

Entry 3: The reaction was performed on a 0.05 mmol scale following **general procedure B**. Compound **2a** (26.7 mg, 0.05 mmol, 1.0 equiv.) was dissolved in DCM (2 mL). Pyrrolidine (10 μL, 0.13 mmol, 2.5 equiv.) was then added to the flask. The

resulting solution was stirred for 0.5 h at room temperature. Upon completion indicated by TLC, benzylamine (27 μL, 0.25 mmol, 5.0 equiv.) was added to the reaction solution at room temperature. The result was checked by the analysis of the crude reaction using LCMS and it shows that low conversion was performed.

Entry 4: The reaction was performed on a 0.05 mmol scale following **general procedure B**. Compound **2a** (26.7 mg, 0.05 mmol, 1.0 equiv.) was dissolved in DCM (2 mL). Piperazine (10.8 mg, 0.13 mmol, 2.5 equiv.) was then added to the flask. The resulting solution was stirred for 2.5 h at room temperature. Upon completion indicated by TLC, benzylamine (27 μL, 0.25 mmol, 5.0 equiv.) was added to the reaction solution for 2 h at room temperature. The result was checked by the analysis of the crude reaction using LCMS. The yield of **5a** was 51%, as determined using ^{1}H NMR spectroscopy.

Entry 5: The reaction was performed on a 0.05 mmol scale following **general procedure B**. Compound **2a** (26.7 mg, 0.05 mmol, 1.0 equiv.) was dissolved in DCM (2 mL). Methyl piperazine (14 μL, 0.13 mmol, 2.5 equiv.) was then added to the flask. The resulting solution was stirred for 2 h at room temperature. Upon completion indicated by TLC, benzylamine (27 μL, 0.25 mmol, 5.0 equiv.) was added to the reaction solution for 3 h at room temperature. The yield of **5a** was 39%, as determined using ^{1}H NMR spectroscopy.

2.2.3. Conditions Optimization for C-Ring Modification

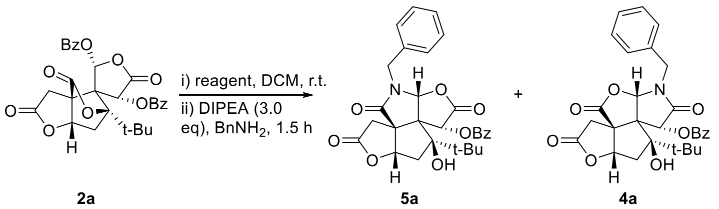

2a **5a** **4a**

General procedure C:

To an oven-dried round bottom flask with a magnetic stir bar was added **2a** (75 mg, 0.14 mmol, 1.0 equiv.), followed by the addition of solvent (2 mL). Reagent was then added. The mixture was stirred until completion indicated by TLC, benzylamine and DIPEA (73 μL, 0.42 mmol, 3.0 equiv.) were added to the reaction solution for 1.5 h. Then the solution was diluted with saturated NH_4Cl solution (10 mL) and DCM (10

mL). After phase separation, the aqueous phase was extracted with DCM (2 x 10 mL). Then the combined organic layers were washed with brine (20 mL), dried over Na_2SO_4, and concentrated *in vacuo*. The crude product was analyzed using LCMS. The regioselectivity of the compound can be determined by the analysis of the crude reaction mixture using ^{1}H NMR spectroscopy. The crude product was purified by column chromatography using hexane/EtOAc to afford **4a** and **5a**.

Entry 1 in Table 2: The reaction was performed on a 0.14 mmol scale following **general procedure C**. Compound **2a** (75 mg, 0.14 mmol, 1.0 equiv.) was dissolved in DCM (2 mL). Morpholine (24 mg, 0.28 mmol, 2.0 equiv.) was then added to the flask. The resulting solution was stirred for 2 h at room temperature. Upon completion indicated by TLC, benzylamine (18 µL, 0.168 mmol, 1.2 equiv.) and DIPEA (73 µL, 0.42 mmol, 3.0 equiv.) were added. The reaction solution was stirred for 1.5 h at room temperature. The products **5a** and **4a** were detected by the analysis of the crude reaction using LCMS. The yields of **5a** and **4a** were 43% and 22%, respectively, as determined by analyzing the crude reaction mixture using ^{1}H NMR spectroscopy.

Entry 2 in Table 2: The reaction was performed on a 0.14 mmol scale following **general procedure C**. Compound **2a** (75 mg, 0.14 mmol, 1.0 equiv.) was dissolved in DCM (2 mL). Piperazine (24 mg, 0.28 mmol, 2.0 equiv.) was then added to the flask. The resulting solution was stirred for 2 h at room temperature. Upon completion indicated by TLC, benzylamine (18 µL, 0.168 mmol, 1.2 equiv.) and DIPEA (73 µL, 0.42 mmol, 3.0 equiv.) were added. The reaction solution was stirred for 1.5 h at room temperature. The products **5a** and **4a** were detected by the analysis of the crude reaction using LCMS. The yields of **5a** and **4a** were 36% and 30% respectively, as determined by analyzing the crude reaction mixture using ^{1}H NMR spectroscopy.

Entry 3 in Table 2: The reaction was performed on a 0.14 mmol scale following **general procedure C**. Compound **2a** (75 mg, 0.14 mmol, 1.0 equiv.) was dissolved in DCM (2 mL). 3,5-dimethyl morpholine (37 µL, 0.28 mmol, 2.0 equiv.) was then added to the flask. The resulting solution was stirred for 2 h at room temperature. Upon

completion indicated by TLC, benzylamine (18 μL, 0.168 mmol, 1.2 equiv.) and DIPEA (73 μL, 0.42 mmol, 3.0 equiv.) were added. The reaction solution was stirred for 1.5 h at room temperature. The products **5a** and **4a** were detected by the analysis of the crude reaction using LCMS. The yields of **5a** and **4a** were 65% and 26%, respectively, as determined by analyzing the crude reaction mixture using ^{1}H NMR spectroscopy.

Entry 4 in Table 2: The reaction was performed on a 0.14 mmol scale following **general procedure C**. Compound **2a** (75 mg, 0.14 mmol, 1.0 equiv.) was dissolved in DCM (2 mL). *Trans*-2,5-dimethylpiperazine (32 mg, 0.28 mmol, 2.0 equiv.) was then added to the flask. The resulting solution was stirred for 2 h at room temperature. Upon completion indicated by TLC, benzylamine (18 μL, 0.168 mmol, 1.2 equiv.) and DIPEA (73 μL, 0.42 mmol, 3.0 equiv.) were added. The reaction solution was stirred for 1.5 h at room temperature. The products **4a** were detected by the analysis of the crude reaction using LCMS. The yield of **4a** was 60%, as determined by analyzing the crude reaction mixture using ^{1}H NMR spectroscopy.

Entry 5 in Table 2: The reaction was performed on a 0.14 mmol scale following **general procedure C**. Compound **2a** (75 mg, 0.14 mmol, 1.0 equiv.) was dissolved in DCM (2 mL). 2,6-dimethyl piperazine (32 mg, 0.28 mmol, 2.0 equiv.) was then added. The solution was stirred for 2 h at room temperature. Upon completion as indicated by TLC, benzylamine (18 μL, 0.168 mmol, 1.2 equiv.) and DIPEA (73 μL, 0.42 mmol, 3.0 equiv.) were added. The reaction was stirred for 1.5 h at room temperature. The product **4a** was detected by LCMS. The yield of **4a** was 98% as determined by analyzing the crude reaction mixture using ^{1}H NMR analysis of the crude reaction mixture.

Entry 6 in Table 2: The reaction was performed on a 0.14 mmol scale following **general procedure C**. Compound **2a** (75 mg, 0.14 mmol, 1.0 equiv.) was dissolved in DCM (2 mL). 1-Boc-piperazine (52 mg, 0.28 mmol, 2.0 equiv.) was then added to the flask. The resulting solution was stirred for 2 h at room temperature. Upon completion indicated by TLC, benzylamine (18 μL, 0.168 mmol, 1.2 equiv.) and DIPEA (73 μL, 0.42 mmol, 3.0 equiv.) were added. The reaction solution was stirred for 1.5 h at room temperature. The products **5a** and **4a** were detected by the analysis of the crude reaction

using LCMS. The yields of **5a** and **4a** were 60% and 13%, respectively, as determined by analyzing the crude reaction mixture using ^{1}H NMR spectroscopy.

Entry 1 in Table 3: The reaction was performed on a 0.14 mmol scale following **general procedure C**. Compound **2a** (75 mg, 0.14 mmol, 1.0 equiv.) was dissolved in DCM (2 mL). 1-Boc-piperazine (52 mg, 0.28 mmol, 2.0 equiv.) was then added to the flask. The resulting solution was stirred for 2 h at room temperature. Upon completion indicated by TLC, benzylamine (18 μL, 0.168 mmol, 1.2 equiv.) and DIPEA (73 μL, 0.42 mmol, 3.0 equiv.) were added. The reaction solution was stirred for 1.5 h at 0 °C. The products **5a** and **4a** were detected by the analysis of the crude reaction using LCMS. The yields of **5a** and **4a** were 35% and 43%, respectively, as determined by analyzing the crude reaction mixture using ^{1}H NMR spectroscopy.

Entry 2 in Table 3: The reaction was performed on a 0.14 mmol scale following **general procedure C**. Compound **2a** (75 mg, 0.14 mmol, 1.0 equiv.) was dissolved in DCM (2 mL). 3,5-dimethyl morpholine (37 μL, 0.28 mmol, 2.0 equiv.) was then added to the flask. The resulting solution was stirred for 2 h at room temperature. Upon completion indicated by TLC, benzylamine (18 μL, 0.168 mmol, 1.2 equiv.) and DIPEA (73 μL, 0.42 mmol, 3.0 equiv.) were added. The reaction solution was stirred for 1.5 h at 0°C. The products **5a** and **4a** were detected by the analysis of the crude reaction using LCMS. The yields of **5a** and **4a** were 28% and 48%, respectively, as determined by analyzing the crude reaction mixture using ^{1}H NMR spectroscopy.

Entry 3 in Table 3: The reaction was performed on a 0.14 mmol scale following **general procedure C**. Compound **2a** (75 mg, 0.14 mmol, 1.0 equiv.) was dissolved in DCM (2 mL). 1-Boc-piperazine (52 mg, 0.28 mmol, 2.0 equiv.) was then added to the flask. The resulting solution was stirred for 2 h at room temperature. Upon completion indicated by TLC, benzylamine (31 μL, 0.28 mmol, 2.0 equiv.) and DIPEA (73 μL, 0.42 mmol, 3.0 equiv.) were added. The solution was stirred for 1.5 h at 0°C. The products **5a** and **4a** were detected by the analysis of the crude reaction using LCMS. The yields of **5a** and **4a** were 50% and 42%, respectively, as determined by analyzing the crude reaction mixture using ^{1}H NMR spectroscopy.

Entry 4 in Table 3: The reaction was performed on a 0.14 mmol scale following **general procedure C**. Compound **2a** (75 mg, 0.14 mmol, 1.0 equiv.) was dissolved in DCM (2 mL). 3,5-dimethyl morpholine (37 μL, 0.28 mmol, 2.0 equiv.) was then added to the flask. The resulting solution was stirred for 2 h at room temperature. Upon completion indicated by TLC, benzylamine (31 μL, 0.28 mmol, 2.0 equiv.) and DIPEA (73 μL, 0.42 mmol, 3.0 equiv.) were added. The reaction solution was stirred for 1.5 h at 0°C. The products **5a** and **4a** were detected by the analysis of the crude reaction using LCMS. The yields of **5a** and **4a** were 29% and 55%, respectively, as determined by analyzing the crude reaction mixture using ^{1}H NMR spectroscopy.

Entry 5 in Table 3: The reaction was performed on a 0.14 mmol scale following **general procedure C**. Compound **2a** (75 mg, 0.14 mmol, 1.0 equiv.) was dissolved in DCM (2 mL). 1-Boc-piperazine (52 mg, 0.28 mmol, 2.0 equiv.) was then added to the flask. The resulting solution was stirred for 2 h at room temperature. Upon completion indicated by TLC, benzylamine (31 μL, 0.28 mmol, 2.0 equiv.) and DIPEA (73 μL, 0.42 mmol, 3.0 equiv.) were added. The reaction solution was stirred for 1.5 h at 40 °C. The products **5a** and **4a** were detected by the analysis of the crude reaction using LCMS. The yields of **5a** and **4a** were 60% and 10%, respectively, as determined by analyzing the crude reaction mixture using ^{1}H NMR spectroscopy.

Entry 6 in Table 3: The reaction was performed on a 0.14 mmol scale following **general procedure C**. Compound **2a** (75 mg, 0.14 mmol, 1.0 equiv.) was dissolved in DCM (2 mL). 3,5-dimethyl morpholine (37 μL, 0.28 mmol, 2.0 equiv.) was then added to the flask. The resulting solution was stirred for 2 h at room temperature. Upon completion indicated by TLC, benzylamine (31 μL, 0.28 mmol, 2.0 equiv.) and DIPEA (73 μL, 0.42 mmol, 3.0 equiv.) were added. The reaction solution was stirred for 1.5 h at 40°C. The products **5a** and **4a** were detected by the analysis of the crude reaction using LCMS. The yields of **5a** and **4a** were 52% and 15%, respectively, as determined by analyzing the crude reaction mixture using ^{1}H NMR spectroscopy.

Entry 7 in Table 3: The reaction was performed on a 0.14 mmol scale following **general procedure C**. Compound **2a** (75 mg, 0.14 mmol, 1.0 equiv.) was dissolved in

DCM (2 mL). 1-Boc-piperazine (52 mg, 0.28 mmol, 2.0 equiv.) was then added to the flask. The resulting solution was stirred for 2 h at room temperature. Upon completion indicated by TLC, benzylamine (31 μL, 0.28 mmol, 2.0 equiv.) and DIPEA (73 μL, 0.42 mmol, 3.0 equiv.) were added. The reaction solution was stirred for 1.5 h at room temperature. The products **5a** and **4a** were detected by the analysis of the crude reaction using LCMS. The yields of **5a** and **4a** were 67% and 14%, respectively, as determined by analyzing the crude reaction mixture using ^{1}H NMR spectroscopy.

Entry 8 in Table 3: The reaction was performed on a 0.14 mmol scale following **general procedure C**. Compound **2a** (75 mg, 0.14 mmol, 1.0 equiv.) was dissolved in DCM (2 mL). 1-Boc-piperazine (52 mg, 0.28 mmol, 2.0 equiv.) was then added to the flask. The resulting solution was stirred for 3.5 h at room temperature. Upon completion indicated by TLC, benzylamine (31 μL, 0.28 mmol, 2.0 equiv.) and DIPEA (73 μL, 0.42 mmol, 3.0 equiv.) were added. The reaction solution was stirred for 1.5 h at room temperature. Product **5a** was detected by the analysis of the crude reaction using LCMS. The yield of **5a** was 70%, as determined by analyzing the crude reaction mixture using ^{1}H NMR spectroscopy.

2.2.4. ^{1}H NMR spectra of the crude product

The analysis of the ratio from the crude reaction mixture is shown below.

^{1}H NMR comparison of the crude and pure product of intermediate **6**

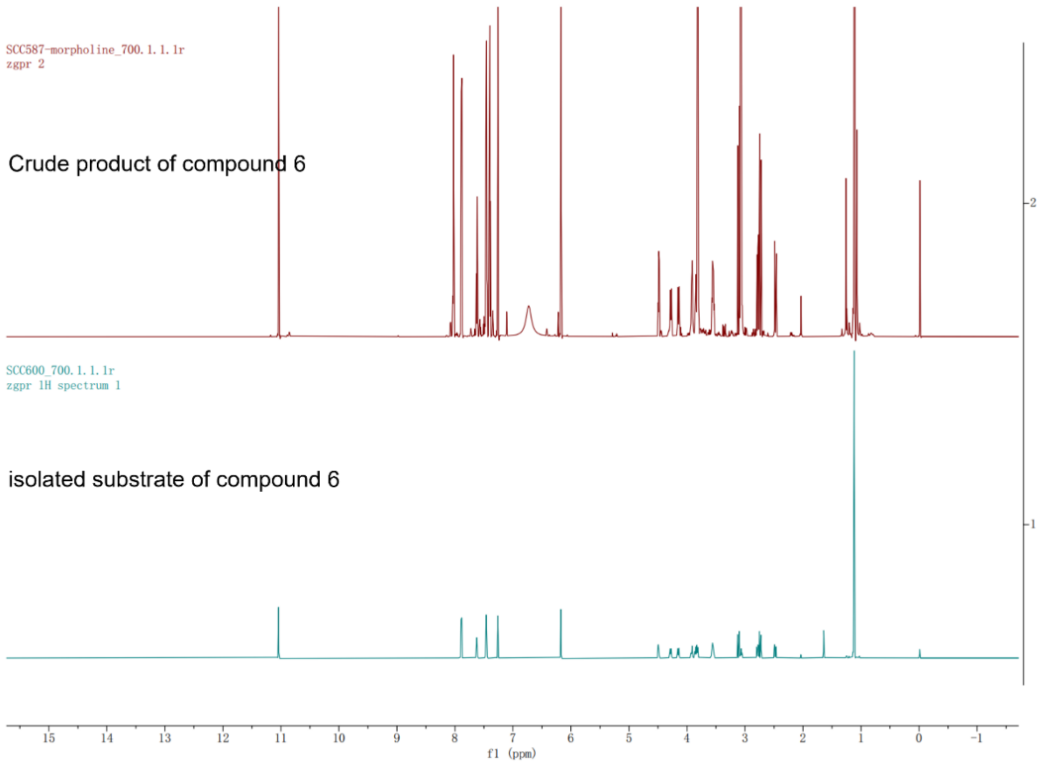

The respective yield was calculated by the ratio of the integrations of the peaks δ 5.15 ppm (compound **5a**) and δ 5.08 ppm (compound **4a**) or δ 4.83 ppm (compound **5a**) and δ 4.90 ppm (compound **4a**).

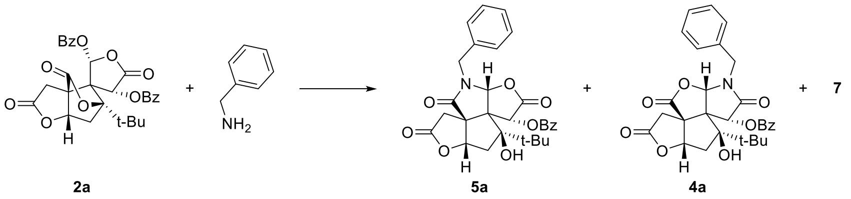

Compound **7** may be one of following structures

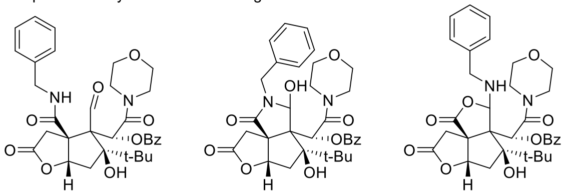

^{1}H NMR of the crude reaction mixture (for the case of **Entry 1 in Table 2**)

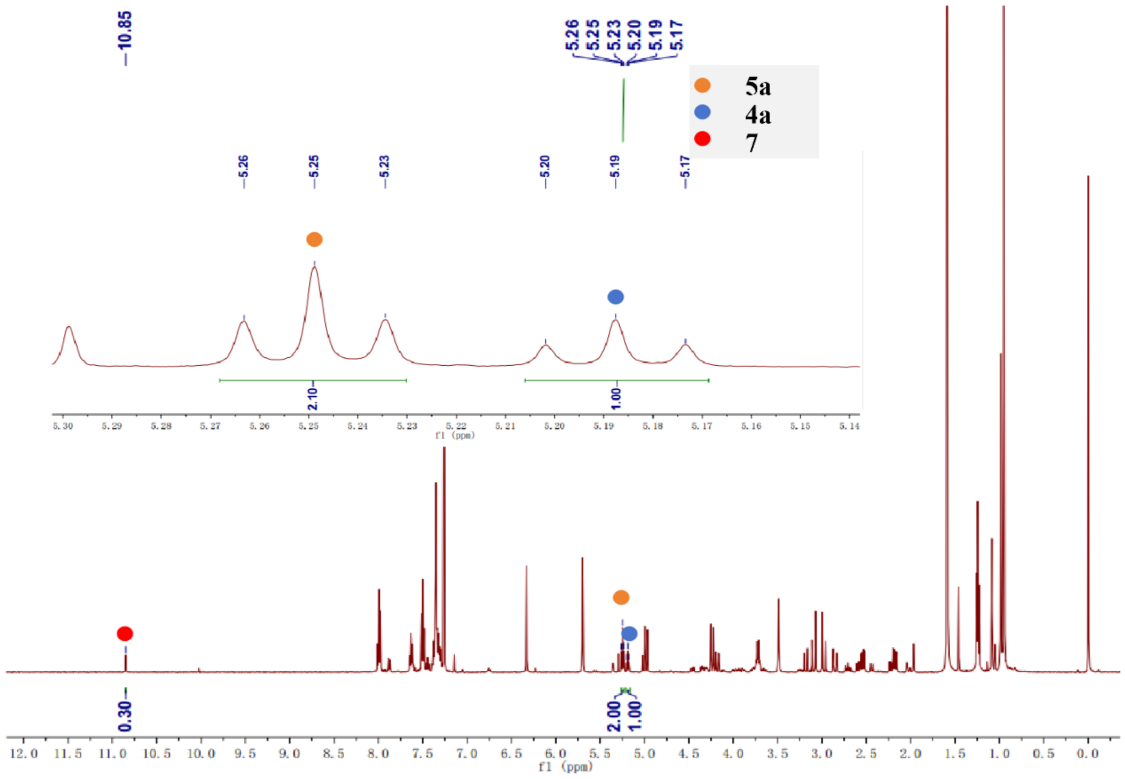

^{1}H NMR of the crude reaction mixture (for the case of **Entry 2 in Table 2**)

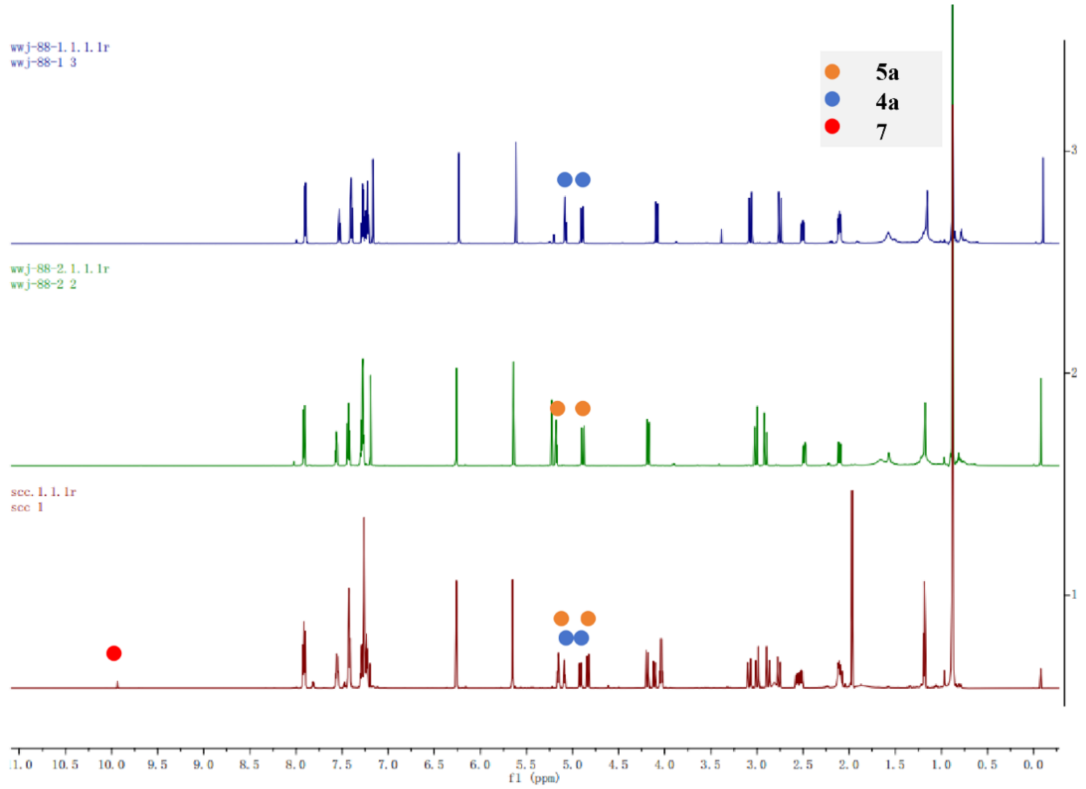

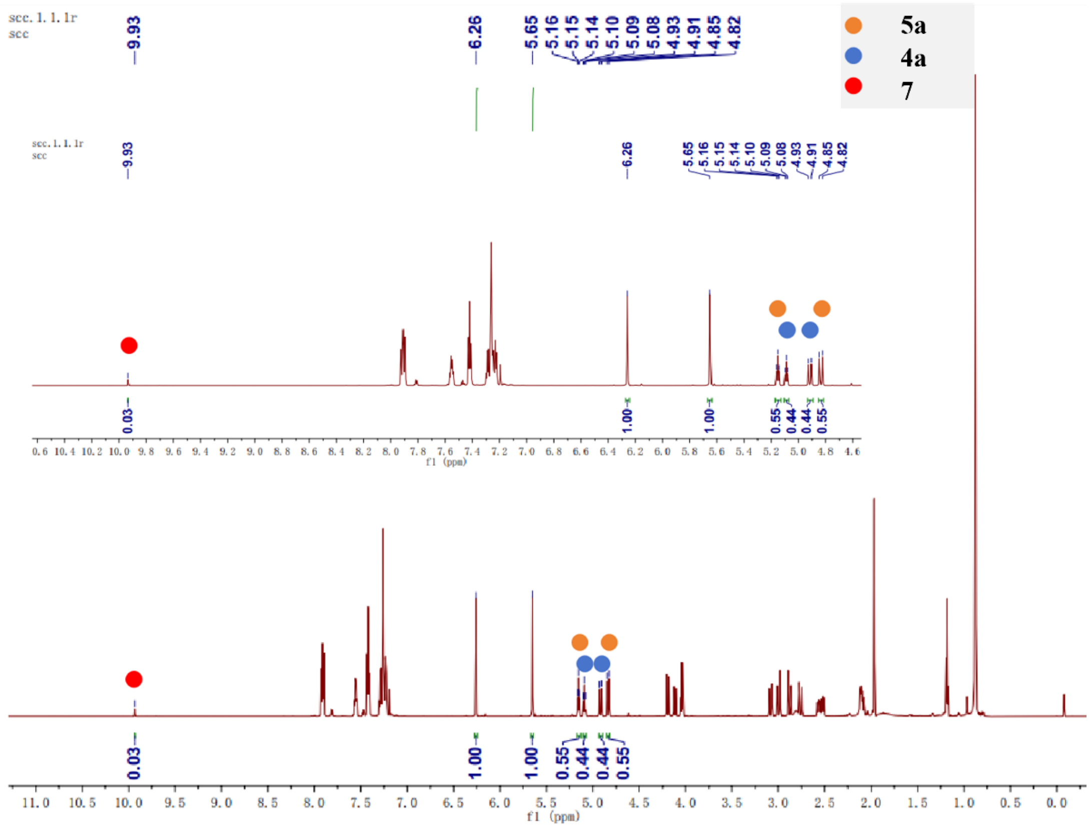

^{1}H NMR of the crude reaction mixture (for the case of **Entry 3 in Table 2**)

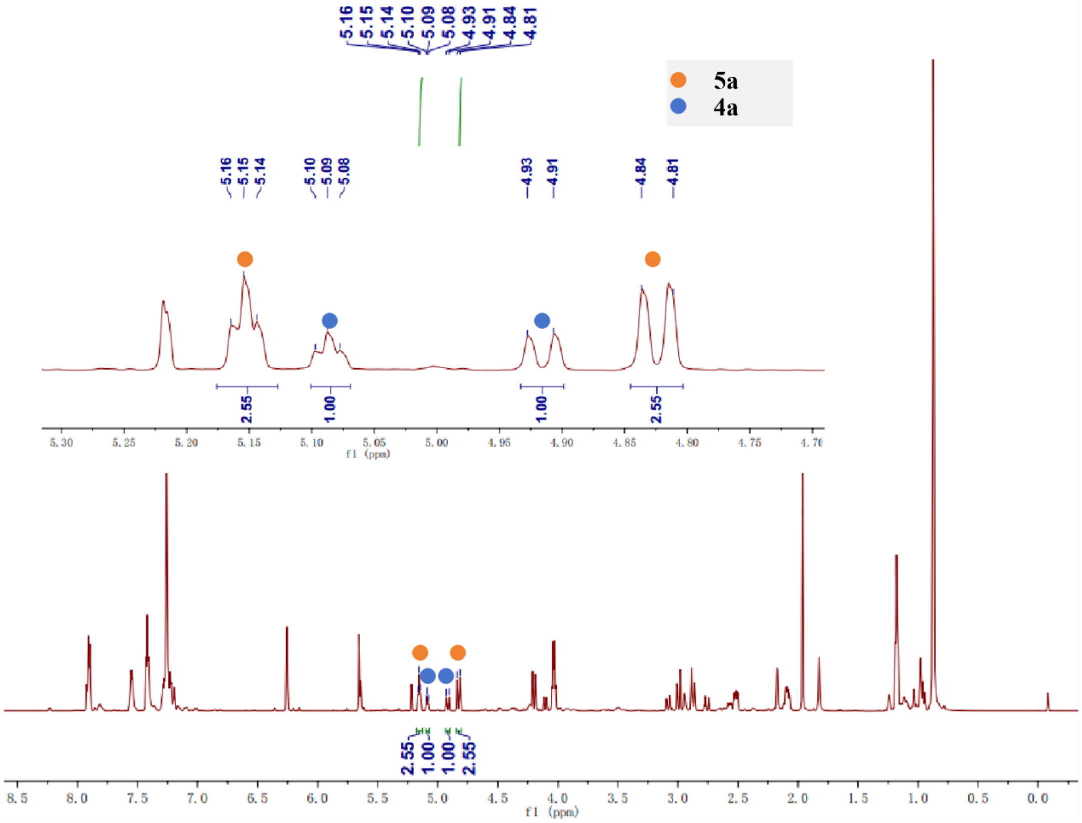

^{1}H NMR of the crude reaction mixture (for the case of **Entry 6 in Table 2**)

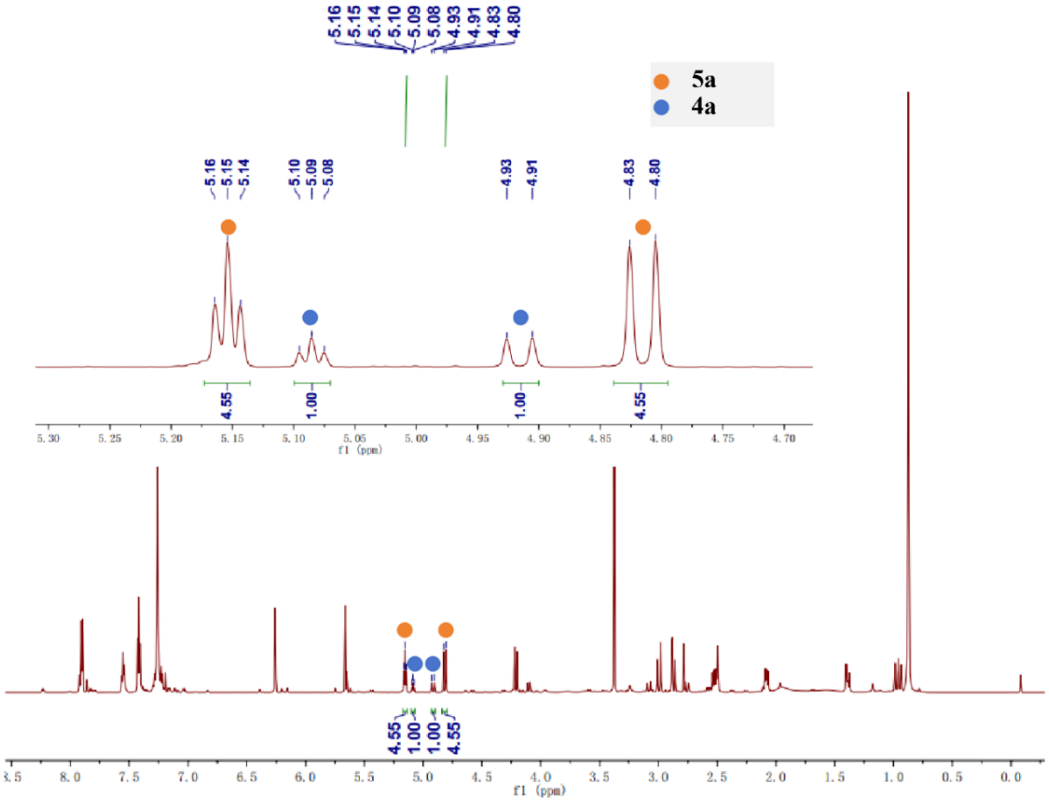

^{1}H NMR of the crude reaction mixture (for the case of **Entry 1 in Table 3**)

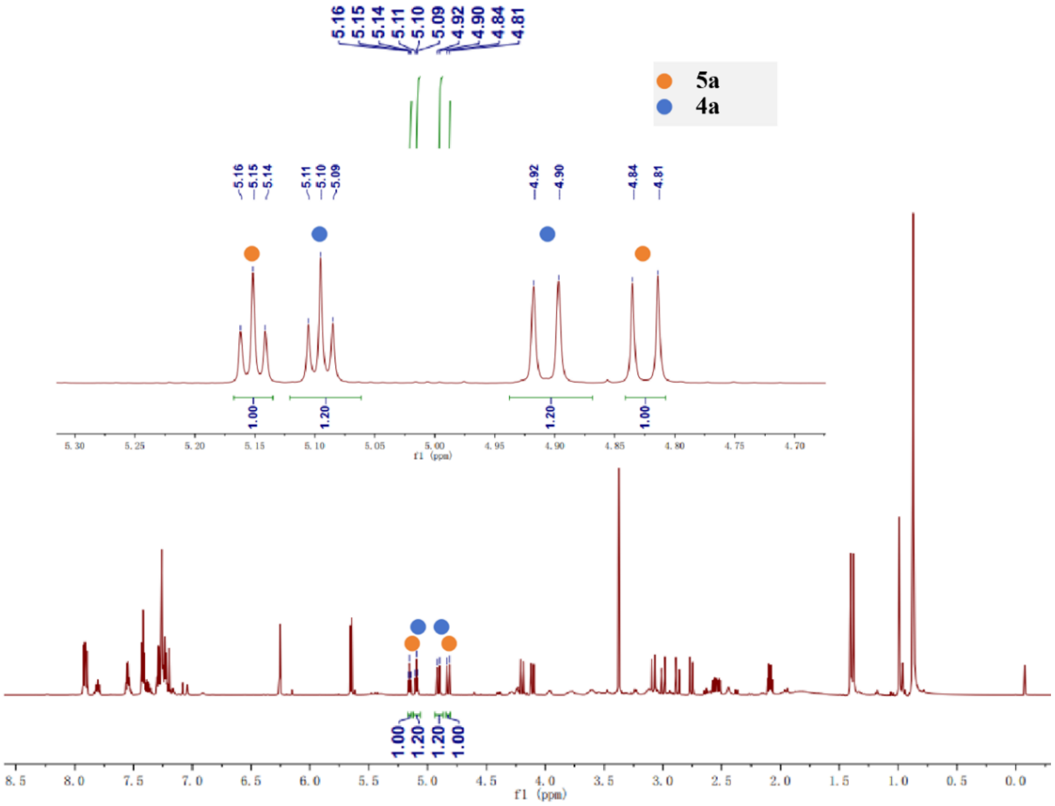

^{1}H NMR of the crude reaction mixture (for the case of **Entry 2 in Table 3**)

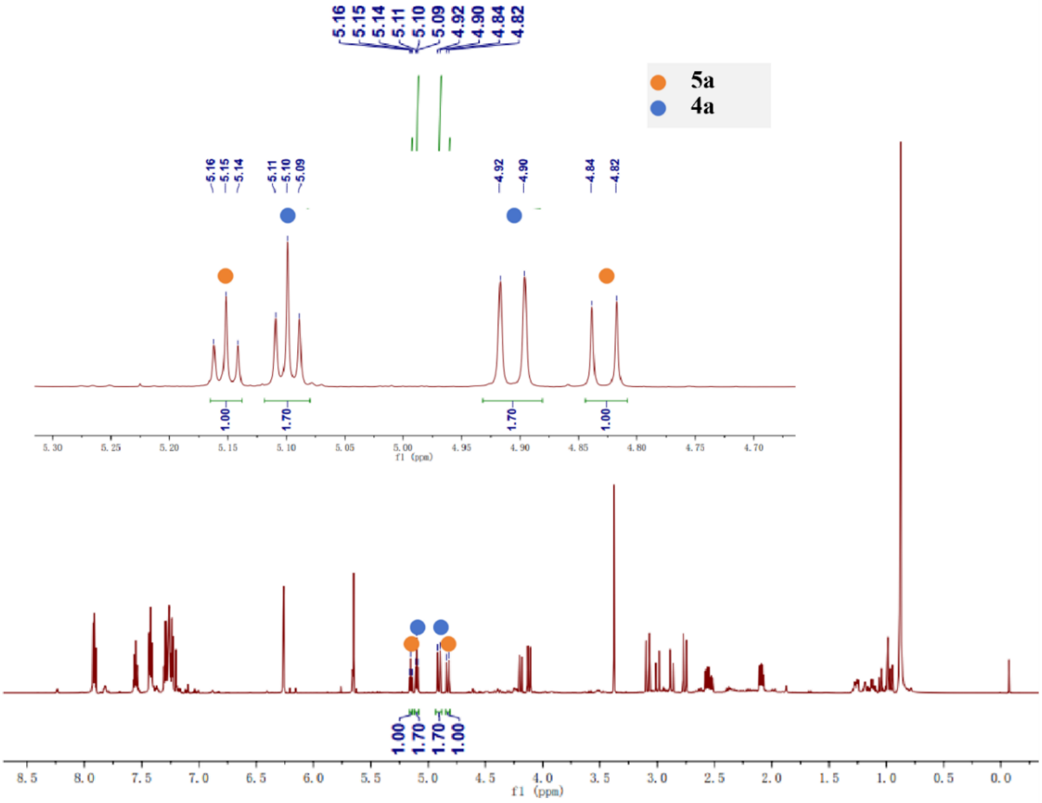

^{1}H NMR of the crude reaction mixture (for the case of **Entry 3 in Table 3**)

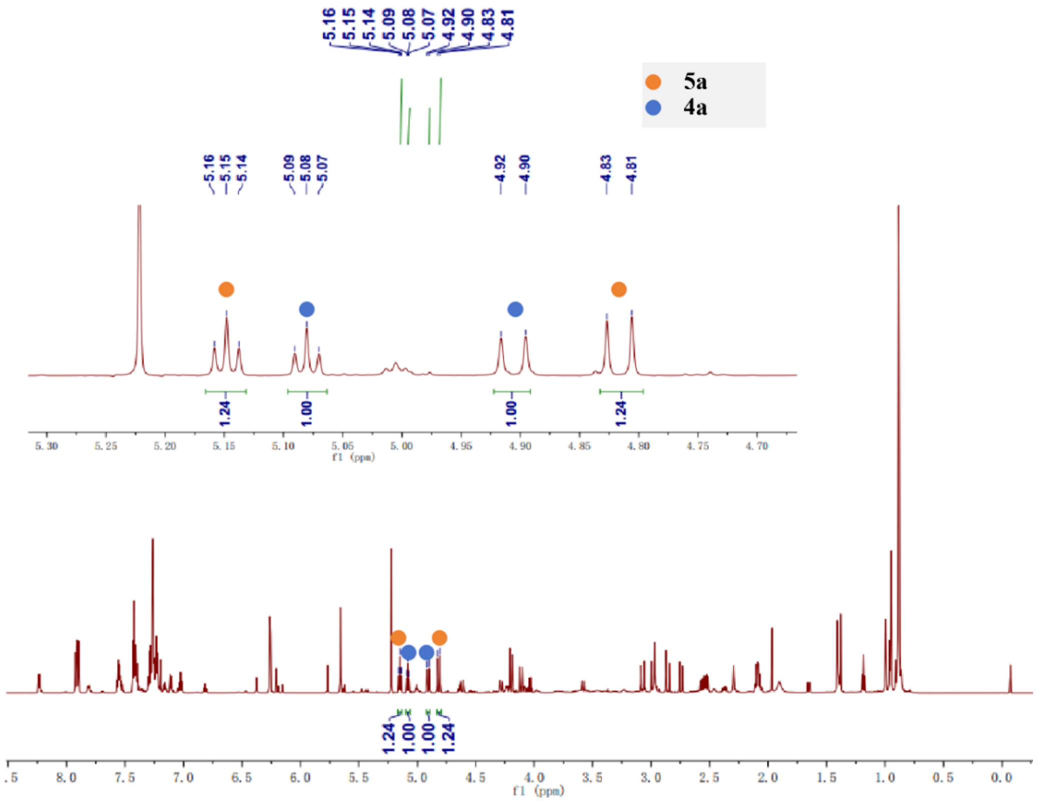

^{1}H NMR of the crude reaction mixture (for the case of **Entry 4 in Table 3**)

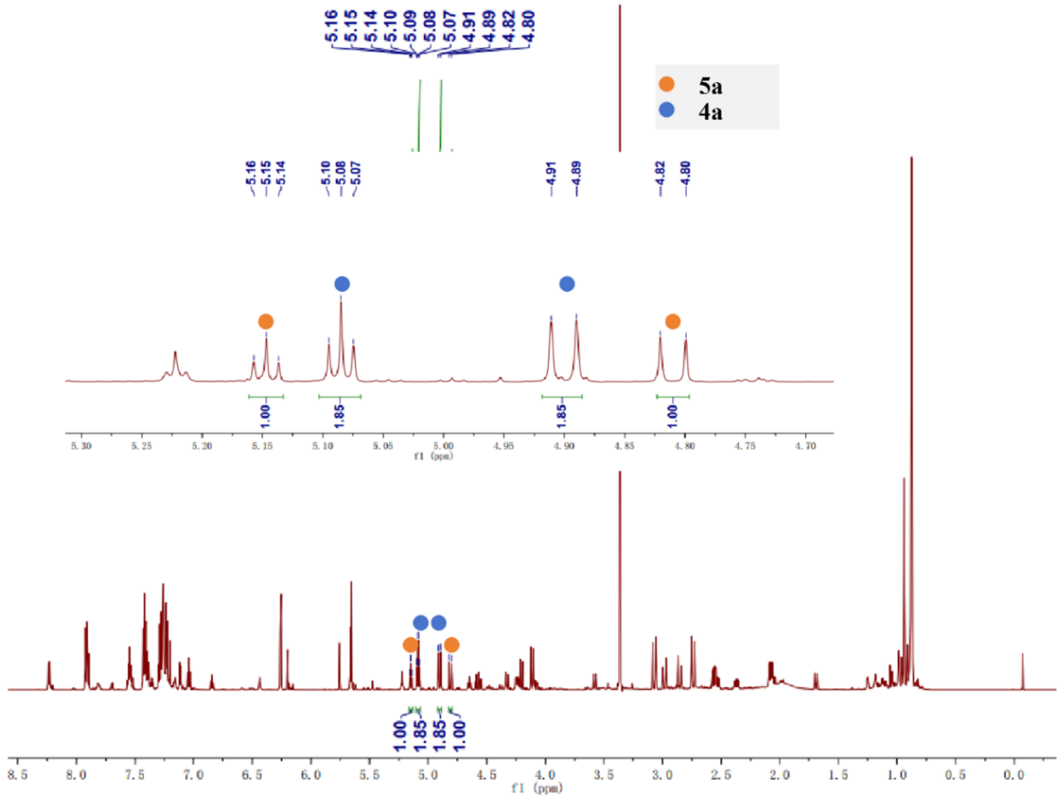

^{1}H NMR of the crude reaction mixture (for the case of **Entry 5 in Table 3**)

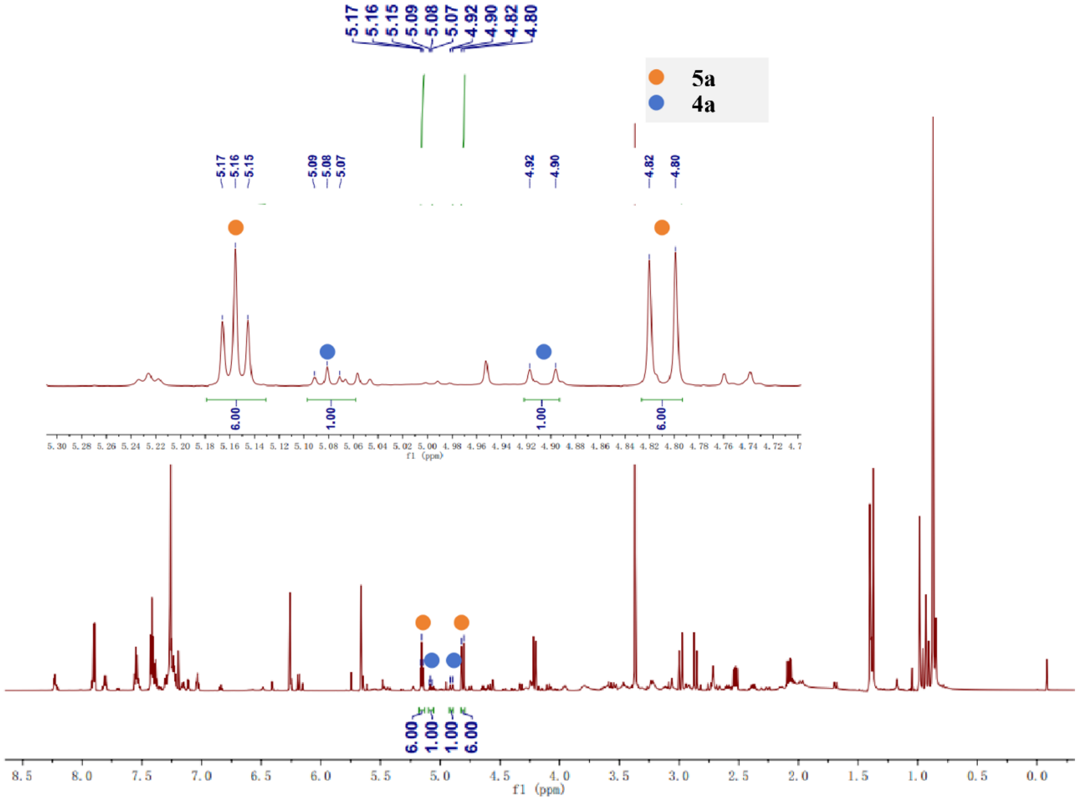

¹H NMR of the crude reaction mixture (for the case of **Entry 6 in Table 3**)

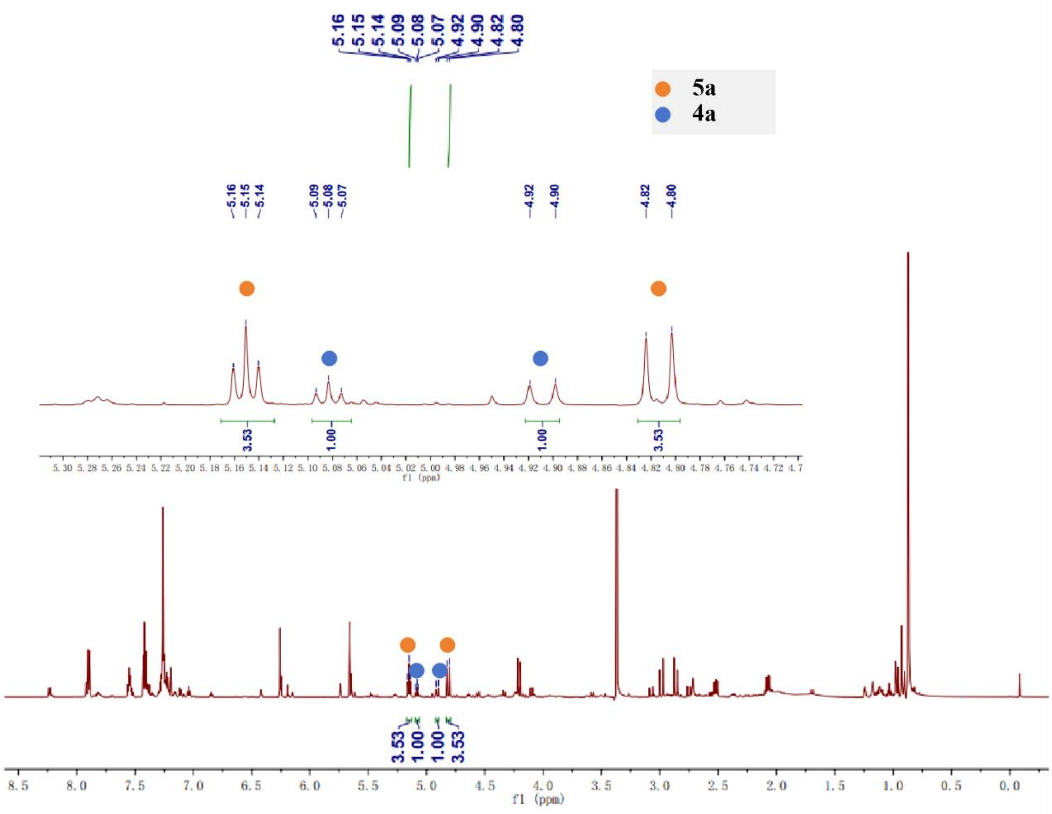

¹H NMR of the crude reaction mixture (for the case of **Entry 7 in Table 3**)

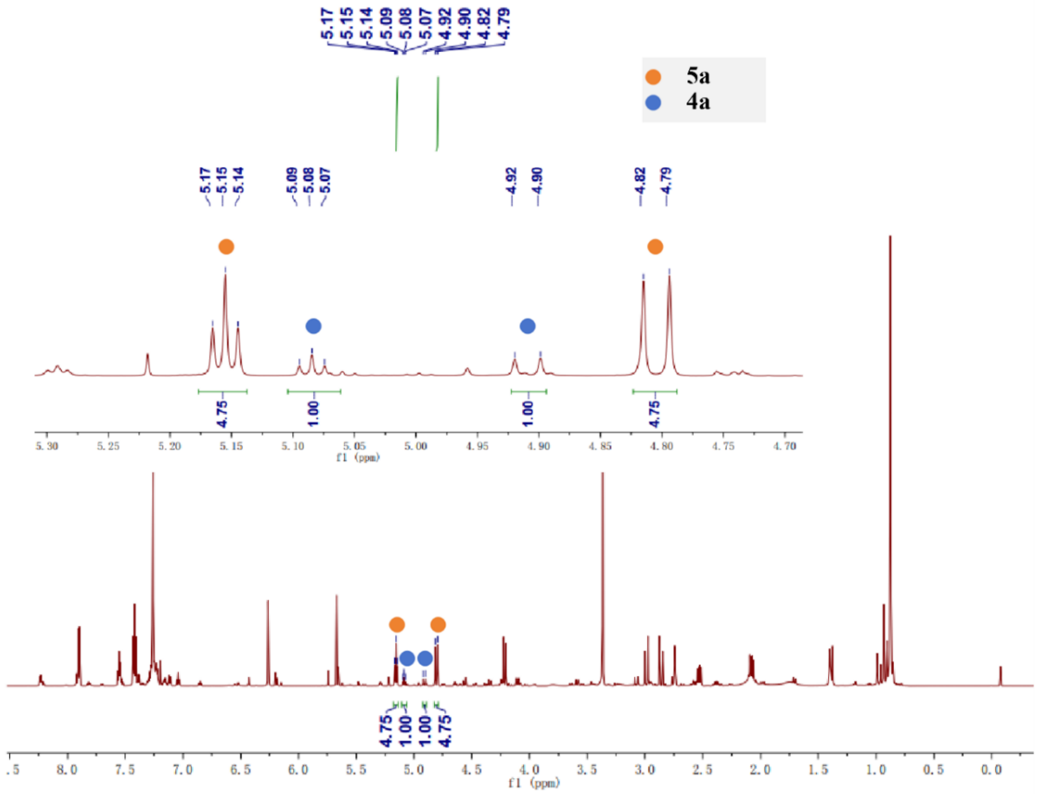

3. Synthesis Procedures and Analytical Data

3.1. General Procedure D: Synthesis of Dibenzoylated Analogues 2a-2i

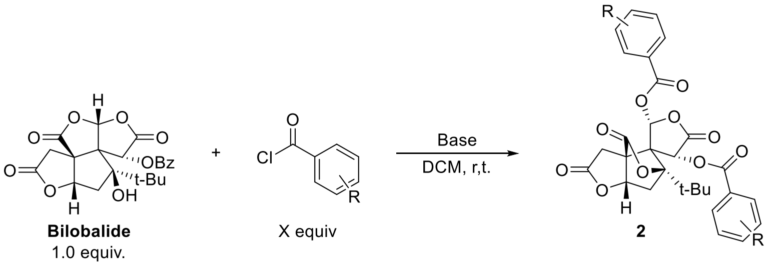

To an oven-dried flask containing a magnetic stirrer bar was added bilobalide (0.10 g, 0.31 mmol, 1.0 equiv.), followed by the addition of anhydrous dichloromethane (DCM, 3.0 mL). Then aromatic acyl chloride (3.0 equiv., 0.92 mmol) and TMEDA (138 µL, 0.92 mmol, 3.0 equiv.) were added. The reaction was stirred under nitrogen atmosphere. The reaction was monitored by TLC (reaction time usually between 3 and 18h). Then the reaction solution was diluted with saturated NH_4Cl solution (10 mL) and DCM (10 mL). After phase separation, the aqueous phase was extracted with DCM (2 x 10 mL). The combined organic layers were washed 3 times with saturated $NaHCO_3$ aqueous solution (10 mL). After that, the organic layers were washed with brine (1 x 10 mL), dried over anhydrous Na_2SO_4, and concentrated *in vacuo*. The crude was then purified by column chromatography using elution system (hexane/EtOAc/DCM= 4:1:1→ 3:1:1, v/v) to afford dibenzoylated **2**.

*Note: For larger scale reactions, pure **2** can be obtained by crystallization in Hex/EA (1:1) from the crude.*

(2*R*,3a'*S*,4*R*,6'*R*,7a'*S*)-6'-(*tert*-Butyl)-2',4',5-trioxohexahydro-4'*H*,6'*H*-spiro[furan-3,8'-[3a,6]methanofuro[3,2-*c*]pyran]-2,4-diyl dibenzoate (2a)

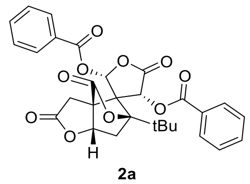

2a

Using **general procedure D** with benzoyl chloride (107 µL, 0.93 mmol, 3.0 equiv.); yield 98% (0.304 mmol, 162 mg); white powder.

Notes: Compound **2a** was also obtained at 4.60 mmol scale. To an oven-dried flask containing a magnetic stirrer bar was added bilobalide (1.50 g, 4.60 mmol, 1.0 equiv.) in anhydrous dichloromethane (DCM, 50 mL). Then benzoyl chloride (1.60 mL, 13.80 mmol, 3.0 equiv.) and TMEDA (2.07 mL, 13.8 mmol, 3.0 equiv.) were added. The reaction was stirred under nitrogen atmosphere for 3 h. Then the reaction solution was diluted with saturated NH_4Cl solution (40 mL). After phase separation, the aqueous phase was extracted with DCM (2 x 30 mL). The combined organic layer was washed twice with saturated $NaHCO_3$ aqueous solution (40 mL). After that, the organic layer was washed with brine (30 mL), dried over anhydrous $MgSO_4$, and concentrated in vacuo. The crude was washed with hexane/EA (2:1) to afford **2a** as white powder. The yield of **2a** is 93% (2.28 g).

R$_f$ = 0.41 (hexane: EtOAc = 3:1, UV detection on TLC plate). **^{1}H NMR (700 MHz, $CDCl_3$)** δ 8.08 (d, *J* = 7.9 Hz, 2H), 8.02 (d, *J* = 8.2 Hz, 2H), 7.73 (d, *J* = 8.0 Hz, 1H), 7.67 (d, *J* = 7.9 Hz, 1H), 7.60 – 7.54 (m, 2H), 7.53 – 7.47 (m, 2H), 7.35 (t, *J* = 5.0 Hz, 1H), 6.23 (s, 1H), 4.49 – 4.42 (m, 1H), 3.36 (d, *J* = 15.7 Hz, 1H), 3.09 (d, *J* = 17.7 Hz, 1H), 2.88 – 2.77 (m, 2H), 1.26 (s, 9H). **^{13}C NMR (176 MHz, $CDCl_3$)** δ 173.2, 169.1, 168.9, 163.7, 135.0, 134.6, 130.2, 129.3, 128.8, 128.1, 100.9, 94.3, 78.6, 67.5, 63.9, 62.7, 35.8, 34.8, 32.4, 26.5. **HRMS (ESI-Orbitrap) m/z:** $[M + Na]^+$ Calcd for $C_{29}H_{26}O_{10}Na^+$ 557.1418; found 557.1414.

(2*R*,3a'*S*,4*R*,6'*R*,7a'*S*)-6'-(*tert*-butyl)-2',4',5-trioxohexahydro-4'*H*,6'*H*-spiro[furan-3,8'-[3a,6]methanofuro[3,2-*c*]pyran]-2,4-diyl bis(4-methylbenzoate) (2b)

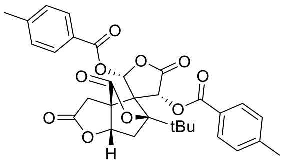

2b

Using **general procedure D** with p-toluoyl chloride (123 µL, 0.93 mmol, 3.0 equiv.); yield 94% (291 µmol, 164 mg); white powder.

R$_f$ = 0.57 (hexane/EtOAc = 2:1); **^{1}H NMR (700 MHz, $CDCl_3$)** δ [ppm] = 7.97 (d, *J* = 8.2 Hz, 2H), 7.92 (d, *J* = 8.2 Hz, 2H), 7.36 (d, *J* = 8.0 Hz, 2H), 7.35 (s, 1H), 7.31 (d, *J*

= 8.0 Hz, 2H), 6.22 (s, 1H), 4.44 (dd, *J* = 7.9, 4.3 Hz, 1H), 3.36 (d, *J* = 17.9 Hz, 1H), 3.07 (d, *J* = 17.9 Hz, 1H), 2.86 (dd, *J* = 15.2, 4.3 Hz, 1H), 2.81 (dd, *J* = 15.2, 7.9 Hz, 1H), 2.50 (s, 3H), 2.46 (s, 3H), 1.26 (s, 9H). ^{13}C NMR (176 MHz, $CDCl_3$) δ 173.2, 169.3, 169.0, 163.7, 146.2, 145.7, 130.3, 130.2, 129.9, 129.5, 125.3, 124.9, 100.9, 94.1, 78.6, 67.4, 63.9, 62.7, 35.8, 34.8, 32.3, 26.5, 22.1. **HRMS (ESI-Orbitrap) m/z:** $[M + Na]^+$ Calcd for $C_{31}H_{30}O_{10}Na^+$ 585.1731; found 585.1736.

(2*R*,3a'*S*,4*R*,6'*R*,7a'*S*)-6'-(*tert*-butyl)-2',4',5-trioxohexahydro-4'*H*,6'*H*-spiro[furan-3,8'-[3a,6]methanofuro[3,2-*c*]pyran]-2,4-diyl bis(4-ethylbenzoate) (2c)

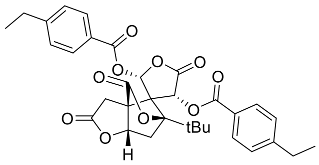

2c

Using **general procedure D** with 4-ethylbenzoyl chloride (138 µL, 0.93 mmol, 3.0 equiv.); yield 87% (270 µmol, 159 mg); white powder.

R*f* = 0.68 (hexane/EtOAc = 2:1); **^{1}H NMR (700 MHz, $CDCl_3$)** δ [ppm] = 8.00 (d, *J* = 8.2 Hz, 2H), 7.96 (d, *J* = 8.3 Hz, 2H), 7.39 (d, *J* = 8.1 Hz, 2H), 7.33 (d, *J* = 8.1 Hz, 2H), 7.30 (d, *J* = 8.0 Hz, 1H), 6.23 (s, 1H), 4.45 (dd, *J* = 7.9, 4.3 Hz, 1H), 3.38 (d, *J* = 17.9 Hz, 1H), 3.08 (d, *J* = 17.9 Hz, 1H), 2.87 (dd, *J* = 15.3, 4.3 Hz, 1H), 2.83 – 2.80 (m, 1H), 2.78 (dd, *J* = 14.3, 6.6 Hz, 2H), 2.76 – 2.73 (m, 2H), 1.32 (t, *J* = 7.6 Hz, 3H), 1.30 – 1.28 (m, 3H), 1.27 (s, 9H). **^{13}C NMR (176 MHz, $CDCl_3$)** δ [ppm] = 173.3, 169.3, 169.0, 163.7, 152.3, 151.7, 150.8, 130.5, 130.4, 128.8, 128.3, 128.2, 125.5, 125.1, 100.9, 94.1, 78.7, 67.4, 63.9, 62.7, 35.8, 34.9, 32.4, 29.3, 29.2, 26.5, 15.3, 15.1. **HRMS (ESI-Orbitrap) m/z:** $[M + Na]^+$ Calcd for $C_{33}H_{34}O_{10}Na^+$ 613.2044; found 613.2051.

(2*R*,3a'*S*,4*R*,6'*R*,7a'*S*)-6'-(*tert*-butyl)-2',4',5-trioxohexahydro-4'*H*,6'*H*-spiro [furan-3,8'-[3a,6]methanofuro[3,2-*c*]pyran]-2,4-diyl bis(4-(dimethylamino) benzoate) (2d)

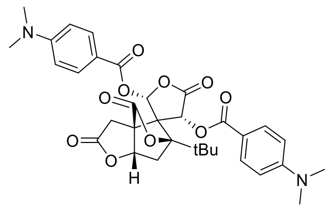

2d

Using **general procedure D** with 4-(dimethylamino)benzoyl chloride (141 µL, 0.93 mmol, 3.0 equiv.); yield 63% (195 µmol, 121 mg); white powder.

R*f* = 0.40 (hexane/EtOAc = 1:1); **^{1}H NMR (700 MHz, CDCl$_3$)** δ [ppm] = 7.93 (d, *J* = 9.0 Hz, 2H), 7.90 (d, *J* = 8.9 Hz, 2H), 7.34 (s, 1H), 6.70 (d, *J* = 9.0 Hz, 2H), 6.66 (d, *J* = 9.0 Hz, 2H), 6.23 (s, 1H), 4.42 (dd, *J* = 8.0, 4.2 Hz, 1H), 3.44 (d, *J* = 17.9 Hz, 1H), 3.11 (s, 6H), 3.10 (s, 1H), 3.07 (s, 6H), 2.87 (dd, *J* = 15.1, 4.2 Hz, 1H), 2.77 (dd, *J* = 15.1, 8.0 Hz, 1H), 1.26 (s, 9H). **^{13}C NMR (176 MHz, CDCl$_3$)** δ [ppm] = 173.5, 170.0, 169.4, 163.9, 163.8, 154.3, 154.2, 132.3, 132.2, 114.2, 113.5, 111.1, 110.7, 100.9, 93.8, 78.7, 66.9, 64.0, 62.7, 40.2, 40.1, 35.7, 34.9, 32.3, 26.6. **HRMS (ESI-Orbitrap) m/z:** $[M + Na]^+$ Calcd for $C_{33}H_{36}N_2O_{10}Na^+$ 643.2262; found 643.2267.

(2*R*,3a'*S*,4*R*,6'*R*,7a'*S*)-6'-(*tert*-butyl)-2',4',5-trioxohexahydro-4'*H*,6'*H*-spiro[furan-3,8'-[3a,6]methanofuro[3,2-*c*]pyran]-2,4-diyl bis(4-fluorobenzoate) (2e)

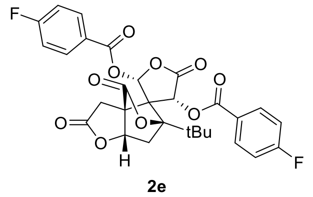

2e

Using **general procedure D** with 4-fluorobenzoyl chloride (110 µL, 0.93 mmol, 3.0 equiv.); yield 77% (239 µmol, 137 mg); white powder.

R*f* = 0.59 (hexane/EtOAc = 3:1); **^{1}H NMR (700 MHz, CDCl$_3$)** δ [ppm] = 8.09 – 8.06 (m, 2H), 8.04 – 8.01 (m, 2H), 7.34 (s, 1H), 7.27 (s, 1H), 7.25 (d, *J* = 8.4 Hz, 1H), 7.18 (t, *J* = 8.5 Hz, 2H), 6.23 (s, 1H), 4.47 (dd, *J* = 7.5, 4.6 Hz, 1H), 3.27 (d, *J* = 17.9 Hz, 1H), 3.12 (d, *J* = 17.8 Hz, 1H), 2.87 – 2.82 (m, 2H), 1.27 (s, 9H). **^{13}C NMR (176 MHz, CDCl$_3$)** δ [ppm] = 173.2, 169.0, 168.8, 167.5, 166.0, 162.9, 162.8, 132.9, 132.8, 124.3, 123.9, 116.8, 116.7, 116.3, 116.1, 101.0, 94.3, 78.7, 67.5, 63.9, 62.7, 35.8, 34.9, 32.4,

26.6. **HRMS (ESI-Orbitrap) m/z:** [M + Na]$^+$ Calcd for $C_{29}H_{24}F_2O_{10}Na^+$ 593.1230; found 593.1229.

(2*R*,3a'*S*,4*R*,6'*R*,7a'*S*)-6'-(*tert*-butyl)-2',4',5-trioxohexahydro-4'*H*,6'*H*-spiro[furan-3,8'-[3a,6]methanofuro[3,2-*c*]pyran]-2,4-diyl bis(2-fluorobenzoate) (2f)

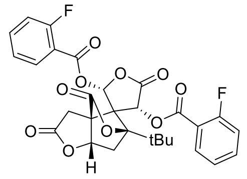

2f

Using **general procedure D** with 2-fluorobenzoyl chloride (111 µL, 0.93 mmol, 3.0 equiv.); yield 86% (267 µmol, 152 mg); white powder.

R*f* = 0.6 (hexane/EtOAc = 2:1); **^{1}H NMR (700 MHz, CDCl$_3$)** δ [ppm] = 8.01 (t, *J* = 7.5 Hz, 2H), 7.70 – 7.61 (m, 2H), 7.33 (d, *J* = 8.6 Hz, 2H), 7.28 (d, *J* = 7.9 Hz, 1H), 7.24 (d, *J* = 8.7 Hz, 1H), 7.20 – 7.16 (m, 1H), 6.25 (s, 1H), 4.44 (t, *J* = 6.1 Hz, 1H), 3.56 (d, *J* = 18.1 Hz, 1H), 3.01 (d, *J* = 18.1 Hz, 1H), 2.82 (d, *J* = 6.1 Hz, 2H), 1.27 (s, 9H). **^{13}C NMR (176 MHz, CDCl$_3$)** δ [ppm] = 173.8, 169.0, 163.2, 162.9, 161.8, 161.4, 136.5, 136.4, 133.1, 132.8, 124.8, 124.4, 117.5, 117.2, 116.4, 116.2, 100.9, 94.2, 78.5, 67.1, 63.6, 62.7, 35.8, 34.8, 31.6, 26.6. **HRMS (ESI-Orbitrap) m/z:** [M + Na]$^+$ Calcd for $C_{29}H_{24}F_2O_{10}Na^+$ 593.1230; found 593.1228.

(2*R*,3a'*S*,4*R*,6'*R*,7a'*S*)-6'-(*tert*-butyl)-2',4',5-trioxohexahydro-4'*H*,6'*H*-spiro[furan-3,8'-[3a,6]methanofuro[3,2-*c*]pyran]-2,4-diyl bis(2-(trifluoromethyl)benzoate) (2g)

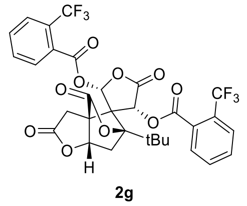

2g

Using **general procedure D** with 2-(trifluoromethyl)benzoyl chloride (137 µL, 0.93 mmol, 3.0 equiv.); yield 90% (279 µmol, 187 mg); white powder.

R*f* = 0.62 (hexane/EtOAc = 1:1); **^{1}H NMR (700 MHz, CDCl$_3$)** δ [ppm] = 7.83 (dd, *J* = 7.7, 3.8 Hz, 2H), 7.72 (d, *J* = 7.6 Hz, 2H), 7.70 – 7.67 (m, 2H), 7.67 – 7.63 (m, 1H),

7.62 (t, J = 7.6 Hz, 1H), 7.22 (s, 1H), 6.19 (s, 1H), 4.40 (dd, J = 7.9, 4.4 Hz, 1H), 2.95 (d, J = 18.2 Hz, 1H), 2.86 (d, J = 6.7 Hz, 1H), 2.83 – 2.80 (m, 1H), 2.77 (dd, J = 15.2, 4.4 Hz, 1H), 1.28 (s, 9H). **^{13}C NMR (176 MHz, CDCl$_3$)** δ [ppm] = 173.3, 169.1, 167.7, 164.1, 163.7, 133.1, 132.32, 132.27, 132.2, 131.9, 129.2 , 128.7, 127.8, 127.2, 127.1, 100.8, 94.1, 78.3, 67.6, 63.6, 62.9, 35.9, 35.0, 31.7, 26.6. **HRMS (ESI-Orbitrap) m/z:** $[M + Na]^+$ Calcd for $C_{31}H_{24}F_6O_{10}Na^+$ 693.1166; found 693.1154.

(2*R*,3a'*S*,4*R*,6'*R*,7a'*S*)-6'-(*tert*-butyl)-2',4',5-trioxohexahydro-4'*H*,6'*H*-spiro[furan-3,8'-[3a,6]methanofuro[3,2-*c*]pyran]-2,4-diyl bis(3-fluorobenzoate) (2h)

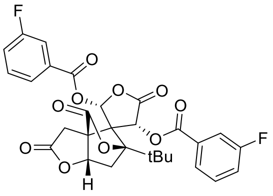

2h

Using **general procedure D** with 3-fluorobenzoyl chloride (113 µL, 0.93 mmol, 3.0 equiv.); yield 76% (236 µmol, 134 mg); white powder.

R*f* = 0.75 (hexane/EtOAc = 2:1); **^{1}H NMR (700 MHz, CDCl$_3$)** δ [ppm] = 7.85 (d, J = 7.8 Hz, 1H), 7.78 (d, J = 7.8 Hz, 1H), 7.72 – 7.68 (m, 2H), 7.54 (dq, J = 20.8, 6.4 Hz, 2H), 7.40 (dt, J = 15.9, 8.1 Hz, 2H), 7.33 (s, 1H), 6.33 (d, J = 1.8 Hz, 1H), 4.49 – 4.46 (m, 1H), 3.29 (d, J = 20.4 Hz, 1H), 3.21 (d, J = 18.7 Hz, 1H), 2.99 (d, J = 15.1 Hz, 1H), 2.66 (dd, J = 14.8, 8.1 Hz, 1H), 1.33 (s, 9H). **^{13}C NMR (176 MHz, CDCl$_3$)** δ [ppm] = 171.9, 168.7, 168.6, 163.6, 163.0, 162.7, 162.1, 131.2, 129.7, 129.3, 125.9, 122.2, 121.9, 117.4, 117.3, 117.1, 117.0, 101.6, 93.2, 79.1, 64.2, 62.7, 61.4, 35.5, 35.2, 30.3, 27.0. **HRMS (ESI-Orbitrap) m/z:** $[M + Na]^+$ Calcd for $C_{29}H_{24}F_2O_{10}Na^+$ 593.1230; found 593.1223.

(2*R*,3a'*S*,4*R*,6'*R*,7a'*S*)-6'-(*tert*-butyl)-2',4',5-trioxohexahydro-4'*H*,6'*H*-spiro[furan-3,8'-[3a,6]methanofuro[3,2-*c*]pyran]-2,4-diyl bis(3-(trifluoromethyl)benzoate) (2i)

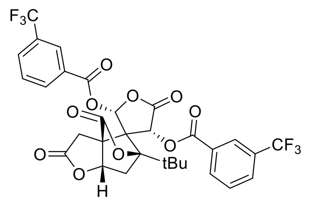

2i

Using **general procedure D** with 3-(trifluoromethyl)benzoyl chloride (140 µL, 0.93 mmol, 3.0 equiv.); yield 96% (298 µmol, 200 mg); white powder.

R*f* = 0.6 (hexane/EtOAc = 2:1); **^{1}H NMR (700 MHz, CDCl$_3$)** δ [ppm] = 8.29 (s, 2H), 8.24 (d, *J* = 8.0 Hz, 1H), 8.18 (d, *J* = 7.9 Hz, 1H), 7.95 (dd, *J* = 13.7, 7.9 Hz, 2H), 7.71 (dt, *J* = 22.3, 7.9 Hz, 2H), 7.36 (d, *J* = 1.9 Hz, 1H), 6.37 (s, 1H), 4.49 (dt, *J* = 7.5, 2.8 Hz, 1H), 3.32 (dd, *J* = 18.5, 1.8 Hz, 1H), 3.24 (dd, *J* = 18.4, 1.8 Hz, 1H), 2.96 (dd, *J* = 17.3, 2.5 Hz, 1H), 2.69 – 2.64 (m, 1H), 1.34 (s, 9H). **^{13}C NMR (176 MHz, CDCl$_3$)** δ [ppm] = 171.8, 168.6, 168.5, 162.9, 162.7, 133.3, 132.9, 131.8, 131.5, 131.2, 130.1, 130.0, 128.5, 128.2, 127.5, 126.8, 124.1, 101.6, 93.4, 79.1, 64.3, 62.7, 61.4, 35.5, 35.2, 30.3, 27.0. **HRMS (ESI-Orbitrap) m/z:** [M + Na]$^+$ Calcd for $C_{31}H_{24}F_6O_{10}Na^+$ 693.1166; found 693.1157.

3.2. General Procedure E: Synthesis of C-Ring modified 5a-5k

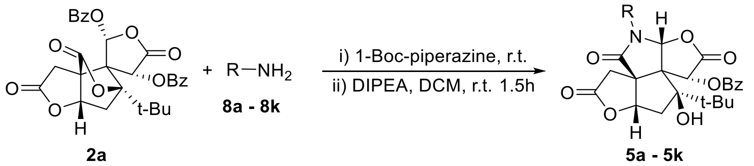

To an oven-dried round bottom flask equipped with a magnetic stir bar was added **2a** (75 mg, 0.14 mmol, 1.0 equiv.) in DCM (2 mL). 1-Boc-piperazine (53 mg, 0.28 mmol, 2.0 equiv.) was then added. The mixture was stirred at room temperature until complete consumption of **2a** as judged by TLC (typically 2-3.5 h). The appropriate amine **8a** to **8k** (0.28 mmol, 2.0 equiv.) and DIPEA (73 µL, 0.42 mmol, 3.0 equiv.) were then added. The reaction solution was stirred for 1.5 h. Then the mixture was diluted with saturated NH_4Cl solution (10 mL) and DCM (10 mL). After phase separation, the aqueous layer was extracted with DCM (2 x 10 mL). The combined organic layers were washed with brine (20 mL), dried over Na_2SO_4, and concentrated *in vacuo*. The crude was analyzed

by LCMS and ^{1}H NMR spectroscopy and purified by column chromatography (hexane/EtOAc) to afford **5a** to **5k**.

(3a*S*,5a*R*,8*R*,9*R*,10a*S*)-5-benzyl-9-(*tert*-butyl)-9-hydroxy-2,4,7-trioxooctahydro-4*H*,9*H*-furo[2,3-*b*]furo[3',2':2,3]cyclopenta[1,2-*c*]pyrrol-8-yl benzoate (5a)

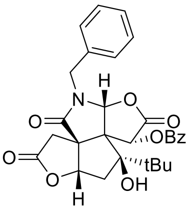

5a

Using **general procedure E** with benzylamine (**8a**, 31 µL, 0.28 mmol, 2.0 equiv.); yield 70% (0.098 mmol, 51 mg); white powder.

R*f* = 0.37 (hexane: EtOAc = 1:1, UV detection on TLC plate). **^{1}H NMR (700 MHz, CDCl$_3$)** δ 7.98 (d, *J* = 7.2 Hz, 2H), 7.63 (t, *J* = 7.5 Hz, 1H), 7.50 (t, *J* = 7.7 Hz, 2H), 7.36 (d, *J* = 5.1 Hz, 2H), 7.35 – 7.34 (m, 2H), 7.34 – 7.30 (m, 1H), 6.33 (s, 1H), 5.70 (s, 1H), 5.25 (t, *J* = 7.2 Hz, 1H), 4.98 (d, *J* = 14.6 Hz, 1H), 4.24 (d, *J* = 14.7 Hz, 1H), 3.49 (s, 1H), 3.09 (d, *J* = 18.9 Hz, 1H), 2.98 (d, *J* = 18.9 Hz, 1H), 2.54 (dd, *J* = 14.0, 7.2 Hz, 1H), 2.18 (dd, *J* = 14.0, 7.3 Hz, 1H), 0.95 (s, 9H). **^{13}C NMR (176 MHz, CDCl$_3$)** δ 176.4, 174.1, 168.2, 164.9, 134.7, 134.2, 130.3, 129.4, 129.0, 128.9, 128.6, 127.4, 89.3, 87.6, 82.6, 70.1, 60.3, 60.2, 51.1, 46.6, 43.3, 37.7, 36.2, 26.5. **HRMS (ESI-Orbitrap) m/z:** [M + Na]$^{+}$ Calcd for $C_{29}H_{29}NO_8Na^{+}$ 542.1785, found 542.1778.

(3a*S*,5a*R*,8*R*,9*R*,10a*S*)-9-(*tert*-butyl)-9-hydroxy-5-(4-methoxybenzyl)-2,4,7-trioxooctahydro-4*H*,9*H*-furo[2,3-*b*]furo[3',2':2,3]cyclopenta[1,2-*c*]pyrrol-8-yl benzoate (5b)

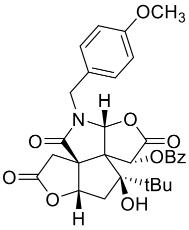

5b

Using **general procedure E** with 4-methoxybenzylamine (**8b**, 37 µL, 0.28 mmol, 2.0 equiv.); yield 49% (0.069 mmol, 38 mg); white powder.

R*f* = 0.21 (hexane: EtOAc = 2:1, UV detection on TLC plate). **^{1}H NMR (700 MHz, $CDCl_3$)** δ 7.99 (d, *J* = 7.5 Hz, 2H), 7.64 (t, *J* = 7.5 Hz, 1H), 7.50 (t, *J* = 7.7 Hz, 2H), 7.27 (d, *J* = 3.6 Hz, 2H), 6.89 (d, *J* = 8.6 Hz, 2H), 6.33 (s, 1H), 5.71 (s, 1H), 5.25 (t, *J* = 7.2 Hz, 1H), 4.90 (d, *J* = 14.6 Hz, 1H), 4.20 (d, *J* = 14.6 Hz, 1H), 3.82 (s, 3H), 3.08 (d, *J* = 18.9 Hz, 1H), 2.98 (d, *J* = 18.9 Hz, 1H), 2.56 (dd, *J* = 14.0, 7.2 Hz, 1H), 2.18 (dd, *J* = 14.0, 7.2 Hz, 1H), 0.97 (s, 9H). **^{13}C NMR (176 MHz, $CDCl_3$)** δ 176.3, 174.2, 168.2, 164.9, 159.7, 134.6, 130.7, 130.3, 129.0, 127.4, 126.1, 114.2, 89.4, 87.5, 82.6, 70.2, 60.3, 60.3, 55.5, 46.0, 43.3, 37.7, 36.2, 26.5. **HRMS (ESI-Orbitrap) m/z:** [M + Na]$^+$ Calcd for $C_{30}H_{31}NO_9Na^+$ 572.1891; found 572.1886.

(3a*S*,5a*R*,8*R*,9*R*,10a*S*)-9-(*tert*-butyl)-5-(2,4-dimethoxybenzyl)-9-hydroxy-2,4,7-trioxooctahydro-4*H*,9*H*-furo[2,3-*b*]furo[3',2':2,3]cyclopenta[1,2-*c*]pyrrol-8-yl benzoate (5c)

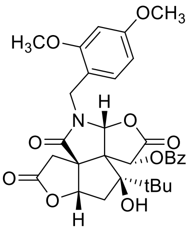

5c

Using **general procedure E** with 2,4-dimethoxybenzylamine (**8c**, 42 μL, 0.28 mmol, 2.0 equiv.); yield 46% (0.065 mmol, 37.5 mg); white powder.

R*f* = 0.68 (hexane: EtOAc = 1:1, UV detection on TLC plate). **^{1}H NMR (700 MHz, $CDCl_3$)** δ 8.00 – 7.97 (m, 2H), 7.64 – 7.61 (m, 1H), 7.51 – 7.48 (m, 2H), 7.28 (d, *J* = 8.4 Hz, 1H), 6.49 (d, *J* = 7.6 Hz, 2H), 6.32 (s, 1H), 5.88 (s, 1H), 5.19 (t, *J* = 7.2 Hz, 1H), 4.66 (d, *J* = 14.3 Hz, 1H), 4.40 (d, *J* = 14.3 Hz, 1H), 3.85 (s, 3H), 3.82 (s, 3H), 3.48 (s, 1H), 3.05 (d, *J* = 18.9 Hz, 1H), 2.95 (d, *J* = 18.9 Hz, 1H), 2.53 (dd, *J* = 13.7, 7.2 Hz, 1H), 2.11 (dd, *J* = 13.8, 7.2 Hz, 1H), 0.99 (s, 9H). **^{13}C NMR (176 MHz, $CDCl_3$)** δ 176.5, 174.3, 168.4, 164.9, 161.5, 158.5, 134.6, 133.0, 130.3, 129.0, 127.5, 115.0, 104.9, 99.1, 89.9, 87.4, 82.9, 70.3, 60.6, 60.0, 56.0, 55.6, 42.9, 41.7, 37.7, 36.1, 29.9, 26.6. **HRMS (ESI-Orbitrap) m/z:** [M + Na]$^+$ Calcd for $C_{31}H_{33}NO_{10}Na^+$ 602.1997; found 602.2001.

(3a*S*,5a*R*,8*R*,9*R*,10a*S*)-5-(((1*R*,3*R*,5*S*)-adamantan-1-yl)methyl)-9-(*tert*-butyl)-9-hydroxy-2,4,7-trioxooctahydro-4*H*,9*H*-furo[2,3-*b*]furo[3',2':2,3]cyclopenta[1,2-*c*]pyrrol-8-yl benzoate (5d)

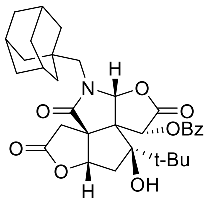

5d

Using **general procedure E** with 1-adamantanemethylamine (**8d**, 50 µL, 0.28 mmol, 2.0 equiv.); yield 44% (0.063 mmol, 36.5 mg); white powder.

R*f* = 0.70 (hexane: EtOAc = 1:1, UV detection on TLC plate). **^{1}H NMR (700 MHz, $CDCl_3$)** δ 8.00 (d, *J* = 7.9 Hz, 2H), 7.64 (t, *J* = 7.4 Hz, 1H), 7.51 (t, *J* = 7.7 Hz, 2H), 6.39 (s, 1H), 5.91 (s, 1H), 5.20 (t, *J* = 7.2 Hz, 1H), 3.10 (d, *J* = 14.4 Hz, 1H), 3.08 – 3.02 (m, 2H), 2.92 (d, *J* = 18.9 Hz, 1H), 2.59 (dd, *J* = 14.1, 7.2 Hz, 1H), 2.25 (dd, *J* = 14.0, 7.3 Hz, 1H), 1.98 (t, *J* = 3.4 Hz, 3H), 1.96 (s, 1H), 1.71 (d, *J* = 12.2 Hz, 3H), 1.65 (s, 2H), 1.61 (d, *J* = 13.3 Hz, 5H), 1.58 – 1.55 (m, 2H), 1.12 (s, 9H). **^{13}C NMR (176 MHz, $CDCl_3$)** δ 178.1, 174.3, 168.2, 164.9, 134.6, 130.3, 129.0, 127.5, 93.1, 87.4, 82.9, 70.4, 60.7, 59.6, 56.4, 43.5, 40.5, 38.0, 36.8, 36.4, 35.2, 29.8, 28.3. **HRMS (ESI-Orbitrap) m/z:** $[M + Na]^+$ Calcd for $C_{33}H_{39}NO_8Na^+$ 600.2568; found 600.2560.

(3a*S*,5a*R*,8*R*,8a*S*,9*R*,10a*S*)-9-(*tert*-butyl)-5-(cyclohexylmethyl)-9-hydroxy-2,4,7-trioxooctahydro-4*H*,9*H*-furo[2,3-*b*]furo[3',2':2,3]cyclopenta[1,2-*c*]pyrrol-8-yl benzoate (5e)

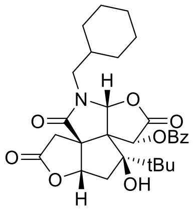

5e

Using **general procedure E** with (aminomethyl)cyclohexane (**8e**, 36 µL, 0.28 mmol, 2.0 equiv.); yield 34% (0.048 mmol, 25 mg); white powder.

R*f* = 0.38 (hexane: EtOAc = 2:1, UV detection on TLC plate). **^{1}H NMR (700 MHz, $CDCl_3$)** δ 8.02 – 7.95 (m, 2H), 7.64 – 7.59 (m, 1H), 7.49 (t, *J* = 7.8 Hz, 2H), 6.36 (s,

1H), 5.93 (s, 1H), 5.21 (t, J = 7.1 Hz, 1H), 3.46 (dd, J = 14.0, 8.4 Hz, 1H), 3.19 (d, J = 18.9 Hz, 1H), 3.07 (dd, J = 14.0, 6.7 Hz, 1H), 2.88 (d, J = 18.9 Hz, 1H), 2.62 (dd, J = 14.0, 7.2 Hz, 1H), 2.25 (dd, J = 14.0, 7.1 Hz, 1H), 1.99 (s, 1H), 1.79 – 1.76 (m, 1H), 1.75 – 1.72 (m, 2H), 1.68 – 1.64 (m, 2H), 1.24 – 1.22 (m, 1H), 1.21 – 1.18 (m, 1H), 1.16 (dt, J = 12.5, 3.1 Hz, 1H), 1.11 (s, 9H), 1.03 – 0.95 (m, 2H). **^{13}C NMR (176 MHz, CDCl$_3$)** δ 178.0, 173.6, 167.2, 165.4, 134.4, 130.2, 128.9, 127.9, 88.2, 87.7, 83.2, 70.8, 62.5, 59.4, 47.0, 43.4, 37.5, 36.6, 35.6, 31.0, 30.9, 26.5, 26.3, 25.7, 25.6. **HRMS (ESI-Orbitrap) m/z:** [M + Na]$^+$ Calcd for $C_{29}H_{35}NO_8Na^+$ 548.2255; found 548.2252.

(3a*S*,5a*R*,8*R*,8a*S*,9*R*,10a*S*)-9-(*tert*-butyl)-9-hydroxy-5-neopentyl-2,4,7-trioxooctahydro-4*H*,9*H*-furo[2,3-*b*]furo[3',2':2,3]cyclopenta[1,2-*c*]pyrrol-8-yl benzoate (5f)

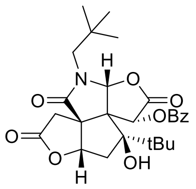

5f

Using **general procedure E** with neopentylamine (**8f**, 33 µL, 0.28 mmol, 2.0 equiv.); yield 34% (0.048 mmol, 24 mg); white powder.

R$_f$ = 0.47 (hexane: EtOAc = 2:1, UV detection on TLC plate). **^{1}H NMR (700 MHz, CDCl$_3$)** δ 7.99 (d, J = 7.8 Hz, 2H), 7.64 (t, J = 7.5 Hz, 1H), 7.50 (t, J = 7.7 Hz, 2H), 6.40 (s, 1H), 5.95 (s, 1H), 5.19 (t, J = 7.2 Hz, 1H), 3.23 (d, J = 14.3 Hz, 1H), 3.16 (d, J = 14.3 Hz, 1H), 3.07 (d, J = 19.0 Hz, 1H), 2.91 (d, J = 19.0 Hz, 1H), 2.61 (dd, J = 14.1, 7.1 Hz, 1H), 2.24 (dd, J = 14.1, 7.3 Hz, 1H), 1.11 (s, 9H), 1.00 (s, 9H). **^{13}C NMR (176 MHz, CDCl$_3$)** δ 178.2, 174.3, 168.2, 164.9, 134.6, 130.3, 129.03, 129.01, 127.5, 92.7, 87.4, 83.0, 70.4, 60.7, 59.6, 55.7, 43.4, 38.0, 36.4, 33.4, 28.0. **HRMS (ESI-Orbitrap) m/z:** [M + Na]$^+$ Calcd for $C_{27}H_{33}NO_8Na^+$ 522.2098; found 522.2096.

(3a*S*,5a*R*,8*R*,9*R*,10a*S*)-9-(*tert*-butyl)-9-hydroxy-5-(3-hydroxy-4-methoxyphenethyl)-2,4,7-trioxooctahydro-4*H*,9*H*-furo[2,3-*b*]furo[3',2':2,3]cyclopenta[1,2-*c*]pyrrol-8-yl benzoate (5g)

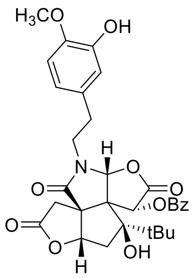

5g

Using **general procedure E** with 3-hydroxy-4-methoxyphenethylamine (**8g**, 47 mg, 0.28 mmol, 2.0 equiv.); yield 51% (0.071 mmol, 41 mg); white powder.

R*f* = 0.37 (hexane: EtOAc = 1:1, UV detection on TLC plate). **^{1}H NMR (700 MHz, CDCl$_3$)** δ 7.98 (d, *J* = 7.8 Hz, 2H), 7.64 (d, *J* = 7.4 Hz, 1H), 7.50 (t, *J* = 7.7 Hz, 2H), 6.81 – 6.77 (m, 2H), 6.71 (d, *J* = 10.4 Hz, 1H), 6.35 (s, 1H), 5.80 (s, 1H), 5.15 (t, *J* = 7.2 Hz, 1H), 3.87 (s, 3H), 3.64 (t, *J* = 7.4 Hz, 2H), 3.02 (d, *J* = 19.0 Hz, 1H), 2.89 – 2.82 (m, 2H), 2.80 (d, *J* = 6.2 Hz, 1H), 2.59 (dd, *J* = 14.0, 7.2 Hz, 1H), 2.17 (dd, *J* = 14.0, 7.2 Hz, 1H), 1.03 (s, 9H). **^{13}C NMR (176 MHz, CDCl$_3$)** δ 176.7, 174.2, 168.2, 164.9, 145.8, 145.6, 134.6, 131.2, 130.3, 129.0, 127.5, 120.3, 115.1, 111.0, 91.2, 87.3, 82.7, 70.1, 60.5, 59.9, 56.1, 44.3, 43.1, 37.8, 36.2, 33.5, 29.8. **HRMS (ESI-Orbitrap) m/z:** $[M + Na]^+$ Calcd for $C_{31}H_{33}NO_{10}Na^+$ 602.1997; found 602.1991.

(3a*S*,5a*S*,8*R*,9*R*,10a*S*)-9-(*tert*-Butyl)-5-(3,4-dihydroxyphenethyl)-9-hydroxy-2,4,7-trioxo-octahydro-4*H*,9*H*-furo[2,3-*b*]furo[3',2':2,3]cyclopenta[1,2-*c*]pyrrol-8-yl benzoate (5h)

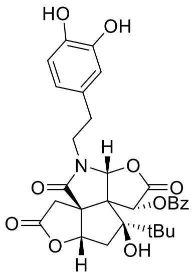

5h

Using **general procedure E** with dopamine hydrochloride (**8h**, 53 mg, 0.28 mmol, 2.0 equiv.), DIPEA (98 μL, 0.56 mmol, 4.0 equiv.); yield 24% (0.034 mmol, 19 mg); white powder.

R*f* = 0.62 (DCM: EtOAc = 1:1, UV detection on TLC plate). **^{1}H NMR (700 MHz, CDCl$_3$)** δ 7.95 (d, *J* = 7.3 Hz, 2H), 7.62 (t, *J* = 7.4 Hz, 1H), 7.48 (t, *J* = 7.7 Hz, 2H), 6.75 (d, *J* = 8.0 Hz, 1H), 6.72 (s, 1H), 6.58 (d, *J* = 8.0 Hz, 1H), 6.36 (s, 1H), 5.85 (s, 1H), 5.14 (t, *J* = 7.2 Hz, 1H), 3.61 (t, *J* = 7.5 Hz, 1H), 3.56 – 3.49 (m, 1H), 2.98 (d, *J* = 19.1 Hz, 1H), 2.80 – 2.69 (m, 3H), 2.56 (dd, *J* = 13.9, 7.2 Hz, 1H), 2.14 – 2.10 (m, 1H), 0.99 (s, 9H). **^{13}C NMR (176 MHz, CDCl$_3$)** δ 177.2, 174.8, 168.6, 164.9, 143.9, 142.9, 134.7, 130.4, 130.2, 129.0, 127.4, 121.2, 116.1, 115.7, 91.5, 87.1, 83.1, 70.2, 60.1, 44.4, 42.8, 37.8, 36.2, 33.3. **HRMS (ESI-Orbitrap) m/z:** [M + Na]$^+$ Calcd for $C_{30}H_{31}NO_{10}Na^+$ 588.1840; found 588.1827.

(3a*S*,5a*R*,8*R*,9*R*,10a*S*)-5-(2-(benzo[*d*][1,3]dioxol-5-yl)ethyl)-9-(*tert*-butyl)-9-hydroxy-2,4,7-trioxooctahydro-4*H*,9*H*-furo[2,3-*b*]furo[3',2':2,3]cyclopenta[1,2-*c*]pyrrol-8-yl benzoate (5i)

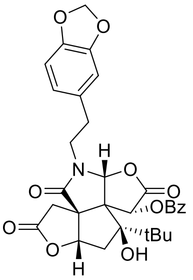

5i

Using **general procedure E** with homopiperonylamine (**8i**, 46 mg, 0.28 mmol, 2.0 equiv.); yield 70% (0.099 mmol, 57 mg); white powder.

R*f* = 0.48 (hexane: EtOAc = 1:1, UV detection on TLC plate). **^{1}H NMR (700 MHz, CDCl$_3$)** δ 8.00 – 7.97 (m, 2H), 7.64 (t, *J* = 7.2 Hz, 1H), 7.50 (t, *J* = 7.7 Hz, 2H), 6.77 – 6.74 (m, 2H), 6.70 (dd, *J* = 7.9, 1.7 Hz, 1H), 6.37 (s, 1H), 5.94 (s, 2H), 5.82 (s, 1H), 5.18 (t, *J* = 7.2 Hz, 1H), 3.68 – 3.62 (m, 2H), 3.03 (d, *J* = 19.0 Hz, 1H), 2.91 – 2.85 (m, 2H), 2.84 (d, *J* = 7.7 Hz, 1H), 2.56 (dd, *J* = 14.1, 7.2 Hz, 1H), 2.20 (dd, *J* = 14.1, 7.3 Hz, 1H), 1.06 (s, 9H). **^{13}C NMR (176 MHz, CDCl$_3$)** δ 176.5, 174.1, 168.1, 165.0, 148.0, 146.6, 134.7, 131.8, 130.3, 129.0, 127.4, 121.8, 109.3, 108.6, 101.2, 91.2, 87.4, 82.6, 70.1, 60.5, 59.9, 44.5, 43.3, 37.8, 36.3, 33.9. **HRMS (ESI-Orbitrap) m/z:** [M + Na]$^+$ Calcd for $C_{31}H_{31}NO_{10}Na^+$ 600.1840; found 600.1835.

(3a*S*,5a*R*,8*R*,9*R*,10a*S*)-9-(*tert*-butyl)-9-hydroxy-5-(4-nitrophenethyl)-2,4,7-trioxooctahydro-4*H*,9*H*-furo[2,3-*b*]furo[3',2':2,3]cyclopenta[1,2-*c*]pyrrol-8-yl benzoate (5j)

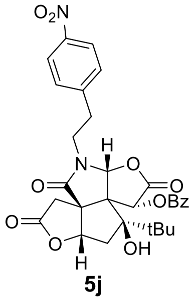

5j

Using **general procedure E** with 4-nitrophenethylamine hydrochloride (**8j**, 57 mg, 0.28 mmol, 2.0 equiv.); yield 44% (0.062 mmol, 36 mg); white powder.

$\mathbf{R}_f$ = 0.46 (hexane: EtOAc = 1:1, UV detection on TLC plate). **^{1}H NMR (700 MHz, CDCl$_3$)** δ 8.19 (d, *J* = 8.6 Hz, 2H), 8.01 – 7.97 (m, 2H), 7.66 – 7.62 (m, 1H), 7.51 (td, *J* = 7.4, 1.1 Hz, 2H), 7.45 – 7.43 (m, 2H), 6.40 (s, 1H), 5.89 (s, 1H), 5.17 (t, *J* = 7.2 Hz, 1H), 3.88 – 3.83 (m, 1H), 3.61 (ddd, *J* = 14.3, 8.5, 5.8 Hz, 1H), 3.09 – 3.04 (m, 2H), 3.03 (d, *J* = 18.9 Hz, 1H), 2.79 (d, *J* = 18.9 Hz, 1H), 2.56 (dd, *J* = 14.3, 7.2 Hz, 1H), 2.24 (dd, *J* = 14.2, 7.2 Hz, 1H), 1.10 (s, 9H). **^{13}C NMR (176 MHz, CDCl$_3$)** δ 176.4, 173.9, 168.0, 164.9, 147.1, 145.6, 134.7, 130.3, 129.9, 129.1, 127.3, 124.0, 91.3, 87.6, 82.4, 70.0, 60.4, 59.9, 43.8, 43.5, 37.9, 36.2, 34.3. **HRMS (ESI-Orbitrap) m/z:** [M + Na]$^+$ Calcd for $C_{30}H_{30}N_2O_{10}Na^+$ 601.1793, found 601.1784.

(3a*S*,5a*R*,8*R*,8a*S*,9*R*,10a*S*)-9-(*tert*-butyl)-9-hydroxy-5-(4-hydroxyphenethyl)-2,4,7-trioxooctahydro-4*H*,9*H*-furo[2,3-*b*]furo[3',2':2,3]cyclopenta[1,2-*c*]pyrrol-8-yl benzoate (5k)

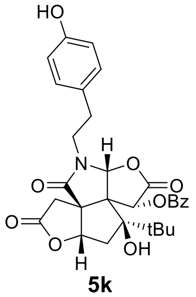

5k

Using **general procedure E** with tyramine (**8k**, 38 mg, 0.28 mmol, 2.0 equiv.); yield 48% (0.067 mmol, 37 mg); white powder.

R*f* = 0.27 (hexane: EtOAc = 2:1, UV detection on TLC plate). **^{1}H NMR (700 MHz, Methanol-d$_4$)** δ 7.94 (d, *J* = 7.3 Hz, 2H), 7.64 (q, *J* = 6.8, 6.3 Hz, 1H), 7.48 (t, *J* = 7.5 Hz, 2H), 7.06 (d, *J* = 8.1 Hz, 2H), 6.69 – 6.64 (m, 2H), 6.46 (s, 1H), 6.12 (s, 1H), 5.08 (t, *J* = 7.1 Hz, 1H), 3.67 (dt, *J* = 13.9, 8.2 Hz, 1H), 3.53 (ddd, *J* = 13.8, 8.2, 5.4 Hz, 1H), 2.84 (dd, *J* = 19.3, 4.4 Hz, 1H), 2.78 (t, *J* = 7.6 Hz, 2H), 2.70 (d, *J* = 19.2 Hz, 1H), 2.63 (dd, *J* = 13.8, 7.1 Hz, 1H), 2.07 (dd, *J* = 13.8, 7.1 Hz, 1H), 1.03 (s, 9H). **^{13}C NMR (176 MHz, Methanol-d$_4$)** δ 178.6, 176.3, 170.3, 166.0, 157.1, 135.5, 131.1, 131.0, 130.5, 130.0, 129.2, 116.2, 93.5, 87.5, 84.9, 71.8, 62.3, 61.3, 45.9, 43.2, 38.8, 37.1, 34.5, 27.0. **HRMS (ESI-Orbitrap) m/z:** $[M + Na]^+$ Calcd for $C_{30}H_{31}NO_9Na^+$ 572.1891, found 572.1884.

4. Biological Experiments

4.1. Cell Culture

Human microglial cells (HMC3; iCell Bioscience) were maintained in Minimum Essential Medium (MEM, Gibco) supplemented with 1× non-essential amino acids (NEAA, Gibco), 1 mM sodium pyruvate (Gibco), 10% fetal bovine serum (FBS, Gibco), and 100 U mL^{-1} penicillin–streptomycin (Gibco). Cells were cultured at 37 °C in a humidified incubator with 5% CO_2.

4.2. Cell viability assay

For cell viability assay, HMC3 cells were seeded in a 96-well plate at a density of 5×10^3 cells per well and allowed to adhere overnight. Cells were pretreated with RSL3 (200 nM; Bidepharm) for 2 h to induce ferroptotic stress, followed by replacement with fresh media containing the bilobalide analogues and incubation for an additional 22 h. Cell viability was quantified using a Cell Counting Kit-8 (CCK-8) assay according to the manufacturer's instructions.

5. X-Ray Data for Compounds 5j and 6

X-Ray Crystallographic Data for **5j** (CCDC: 2535289)

Bond precision:	C-C = 0.0039 A		Wavelength=0.71073
Cell:	a=9.1139(3)	b=12.3766(4)	c=13.1256(5)
	alpha=90	beta=109.970(1)	gamma=90
Temperature:	299 K		
	Calculated	Reported	
Volume	1391.53(8)	1391.53(8)	
Space group	P 21	P 1 21 1	
Hall group	P 2yb	P 2yb	
Moiety formula	C30 H30 N2 O10	C30 H30 N2 O10	
Sum formula	C30 H30 N2 O10	C30 H30 N2 O10	
Mr	578.56	578.56	
Dx,g cm-3	1.381	1.381	
Z	2	2	
Mu (mm-1)	0.105	0.105	
F000	608.0	608.0	
F000’	608.35		
h,k,lmax	12,16,17	12,16,17	
Nref	6707[3508]	6642	
Tmin,Tmax	0.963,0.990	0.656,0.746	
Tmin’	0.959		

Correction method= # Reported T Limits: Tmin=0.656 Tmax=0.746

AbsCorr = MULTI-SCAN

Data completeness= 1.89/0.99 Theta(max)= 27.961

R(reflections)= 0.0386(5564) wR2(reflections)= 0.0935(6642)

S = 1.021 Npar= 384

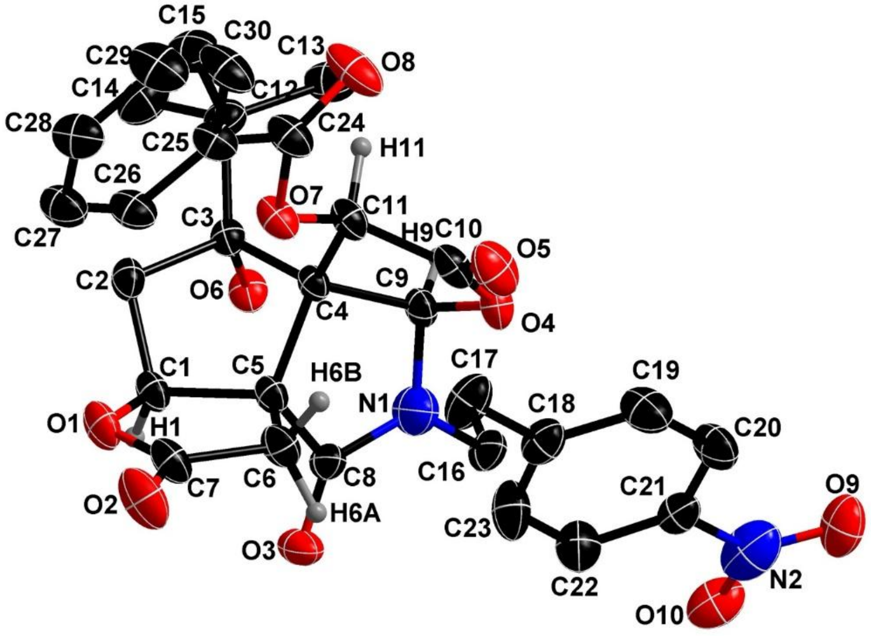

Figure S3. Thermal ellipsoid plot (30% probability level) of the compound **5j** with atom labeling

Table S2. Crystal data and structure refinement for **5j** (CCDC: 2535289)

Empirical formula	$C_{30}H_{30}N_2O_{10}$
Formula weight	578.56
Temperature/K	299.00
Crystal system	monoclinic
Space group	P21
a/Å	9.1139(3)
b/Å	12.3766(4)
c/Å	13.1256(5)
α/°	90
β/°	109.9700(10)
γ/°	90
Volume/Å^3	1391.53(8)
Z	2
ρcalcg/cm^3	1.381
μ/mm^{-1}	0.105
F(000)	608.0
Crystal size/mm^3	0.4 × 0.3 × 0.1
Radiation	MoKα (λ = 0.71073)
2Θ range for data collection/°	4.662 to 55.922
Index ranges	$-12 \leq h \leq 11, -16 \leq k \leq 16, -17 \leq l \leq 17$
Reflections collected	17810
Independent reflections	6642 [R_{int} = 0.0343, R_{sigma} = 0.0396]
Data/restraints/parameters	6642/1/384
Goodness-of-fit on F^2	1.021
Final R indexes [I>=2σ (I)]	R_1 = 0.0386, wR_2 = 0.0878
Final R indexes [all data]	R_1 = 0.0494, wR_2 = 0.0935
Largest diff. peak/hole / e Å^{-3}	0.16/-0.13
Flack parameter	-0.3(4)

X-Ray Crystallographic Data for **6** (CCDC: 2535290)

Bond precision:	C-C = 0.0037 A		Wavelength=0.71073
Cell:	a=10.9266(12)	b=10.6256(10)	c=12.1796(13)
	alpha=90	beta=102.681(3)	gamma=90
Temperature:	199 K		

	Calculated	Reported
Volume	1379.6(2)	1379.6(2)
Space group	P 21	P 1 21 1
Hall group	P 2yb	P 2yb
Moiety formula	C26 H29 N O9 [+ solvent]	C26 H29 N O9
Sum formula	C26 H29 N O9 [+ solvent]	C26 H29 N O9
Mr	499.50	499.50
Dx,g cm-3	1.202	1.202
Z	2	2
Mu (mm-1)	0.091	0.091
F000	528.0	528.0
F000’	528.31	
h,k,lmax	14,14,16	14,14,16
Nref	6898[3632]	6809
Tmin,Tmax	0.973,0.982	0.670,0.746
Tmin’	0.973	

Correction method= # Reported T Limits: Tmin=0.670 Tmax=0.746

AbsCorr = MULTI-SCAN

Data completeness= 1.87/0.99 Theta(max)= 28.336

R(reflections)= 0.0485(6402) wR2(reflections)=0.1470(6809)

S = 1.059 Npar= 328

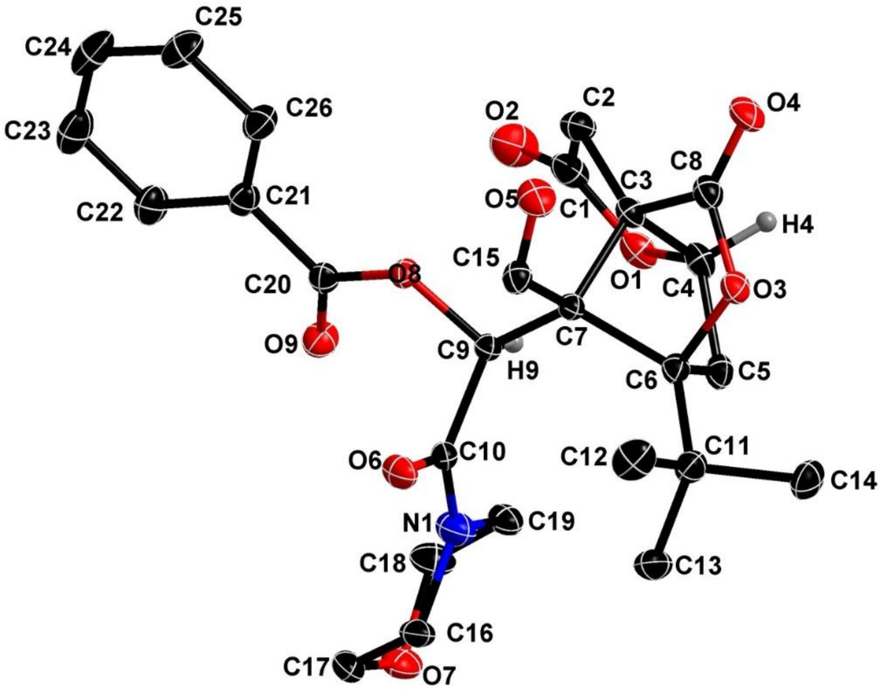

Figure S4. Thermal ellipsoid plot (30% probability level) of the compound **6** with atom labeling

Table S3. Crystal data and structure refinement for **6** (CCDC: 2535290)

Empirical formula	$C_{26}H_{29}NO_9$
Formula weight	499.50
Temperature/K	199.00
Crystal system	monoclinic
Space group	P21
a/Å	10.9266(12)
b/Å	10.6256(10)
c/Å	12.1796(13)
α/°	90
β/°	102.681(3)
γ/°	90
Volume/Å^3	1379.6(2)
Z	2
ρcalcg/cm^3	1.202
μ/mm^{-1}	0.091
F(000)	528.0
Crystal size/mm^3	0.3 × 0.3 × 0.2
Radiation	MoKα (λ = 0.71073)
2Θ range for data collection/°	6.844 to 56.672
Index ranges	$-14 \leq h \leq 14$, $-14 \leq k \leq 14$, $-16 \leq l \leq 16$
Reflections collected	40210
Independent reflections	6809 [R_{int} = 0.0516, R_{sigma} = 0.0384]
Data/restraints/parameters	6809/1/328
Goodness-of-fit on F^2	1.059
Final R indexes [I>=2σ (I)]	R_1 = 0.0485, wR_2 = 0.1449
Final R indexes [all data]	R_1 = 0.0507, wR_2 = 0.1470
Largest diff. peak/hole / e Å^{-3}	0.28/-0.31
Flack parameter	-0.76(16)

Sample Preparation: Single crystals of compounds **5j** and **6** suitable for X-ray diffraction analysis were obtained by slow evaporation of a solution in a 1:1 (v/v) mixture of dichloromethane (DCM) and ethyl acetate (EtOAc). Typically, 20 mg of **5j** or **6** was dissolved in the solvent mixture, and the crystals were grown at room temperature over a period of several days.

6. NMR Spectra of final products

6.1. NMR spectra of diBz-Bilobalide analogues 2a-2i

(2*R*,3a'*S*,4*R*,6'*R*,7a'*S*)-6'-(*tert*-butyl)-2',4',5-trioxohexahydro-4'*H*,6'*H*-spiro[furan-3,8'-[3a,6]methanofuro[3,2-*c*]pyran]-2,4-diyl bis(4-methylbenzoate) (2b)

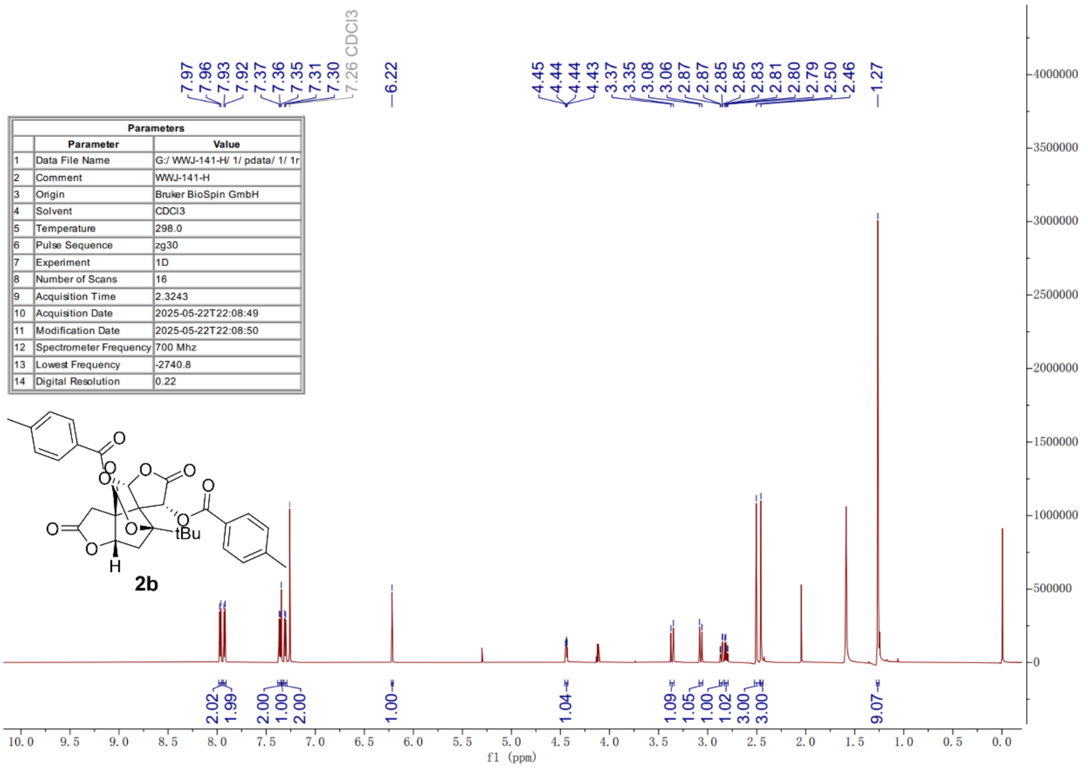

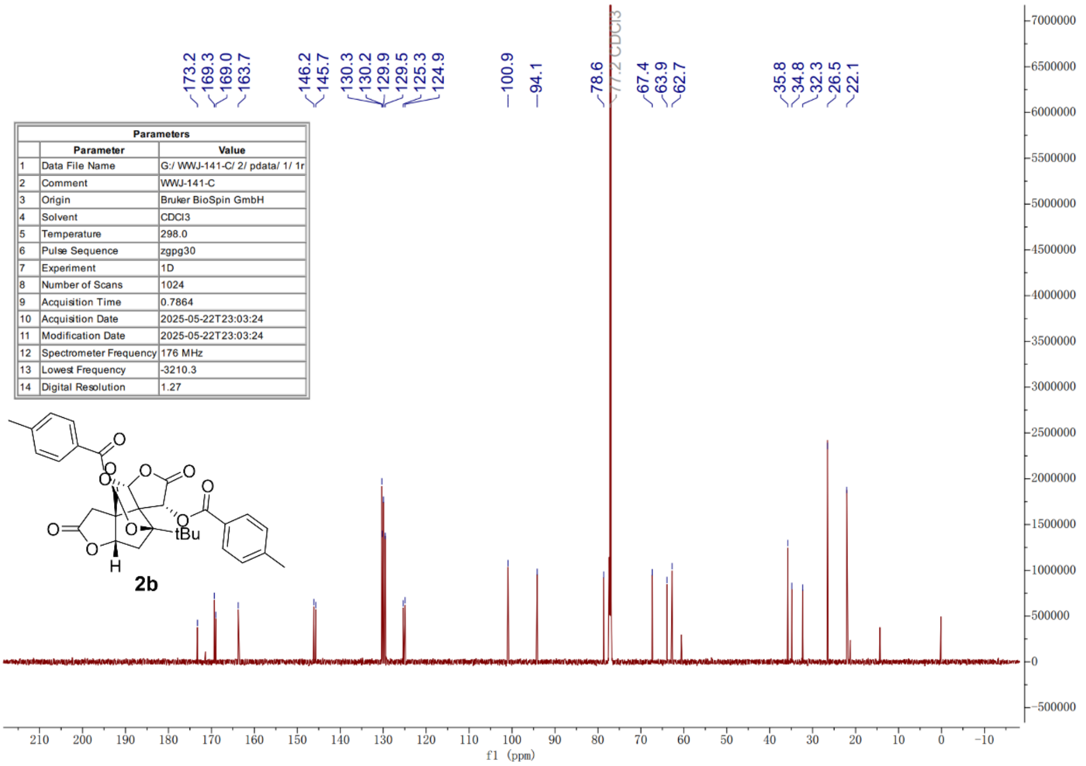

	Parameter	Value
1	Data File Name	G:/ WWJ-141-C/ 2/ pdata/ 1/ 1r
2	Comment	WWJ-141-C
3	Origin	Bruker BioSpin GmbH
4	Solvent	CDCl3
5	Temperature	298.0
6	Pulse Sequence	zgpg30
7	Experiment	1D
8	Number of Scans	1024
9	Acquisition Time	0.7864
10	Acquisition Date	2025-05-22T23:03:24
11	Modification Date	2025-05-22T23:03:24
12	Spectrometer Frequency	176 MHz
13	Lowest Frequency	-3210.3
14	Digital Resolution	1.27

(2*R*,3a'*S*,4*R*,6'*R*,7a'*S*)-6'-(*tert*-butyl)-2',4',5-trioxohexahydro-4'*H*,6'*H*-spiro[furan-3,8'-[3a,6]methanofuro[3,2-*c*]pyran]-2,4-diyl bis(4-ethylbenzoate) (2c)

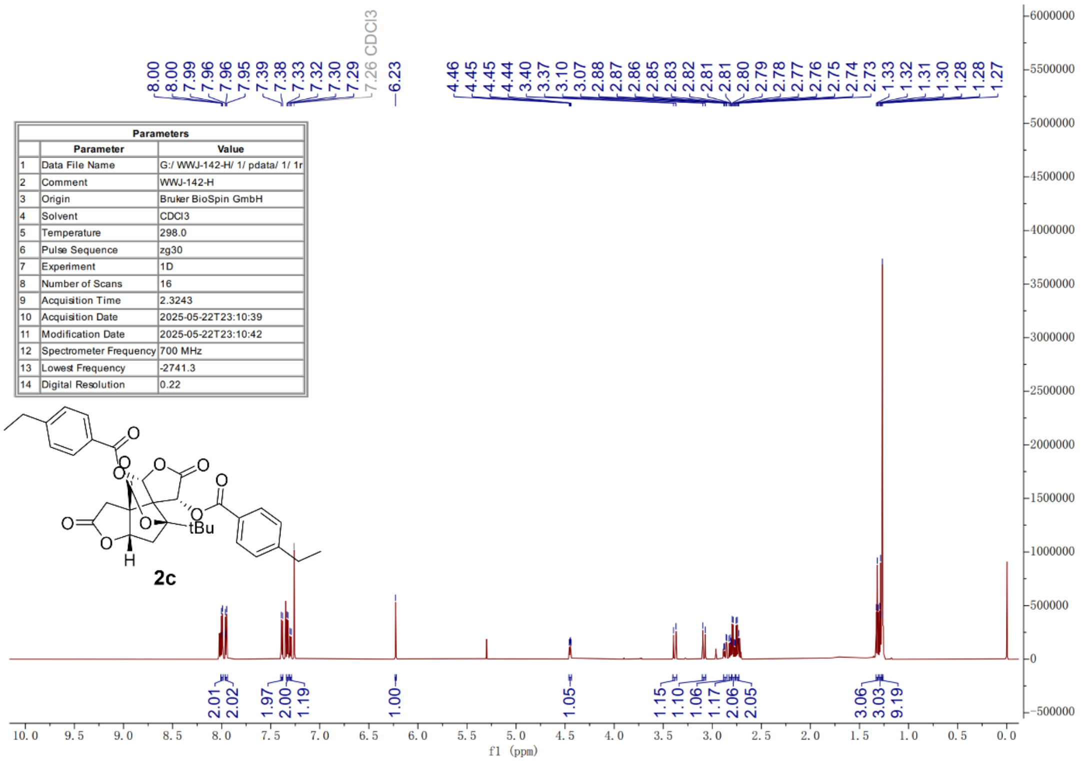

	Parameter	Value
1	Data File Name	G:/ WWJ-142-H/ 1/ pdata/ 1/ 1r
2	Comment	WWJ-142-H
3	Origin	Bruker BioSpin GmbH
4	Solvent	CDCl3
5	Temperature	298.0
6	Pulse Sequence	zg30
7	Experiment	1D
8	Number of Scans	16
9	Acquisition Time	2.3243
10	Acquisition Date	2025-05-22T23:10:39
11	Modification Date	2025-05-22T23:10:42
12	Spectrometer Frequency	700 MHz
13	Lowest Frequency	-2741.3
14	Digital Resolution	0.22

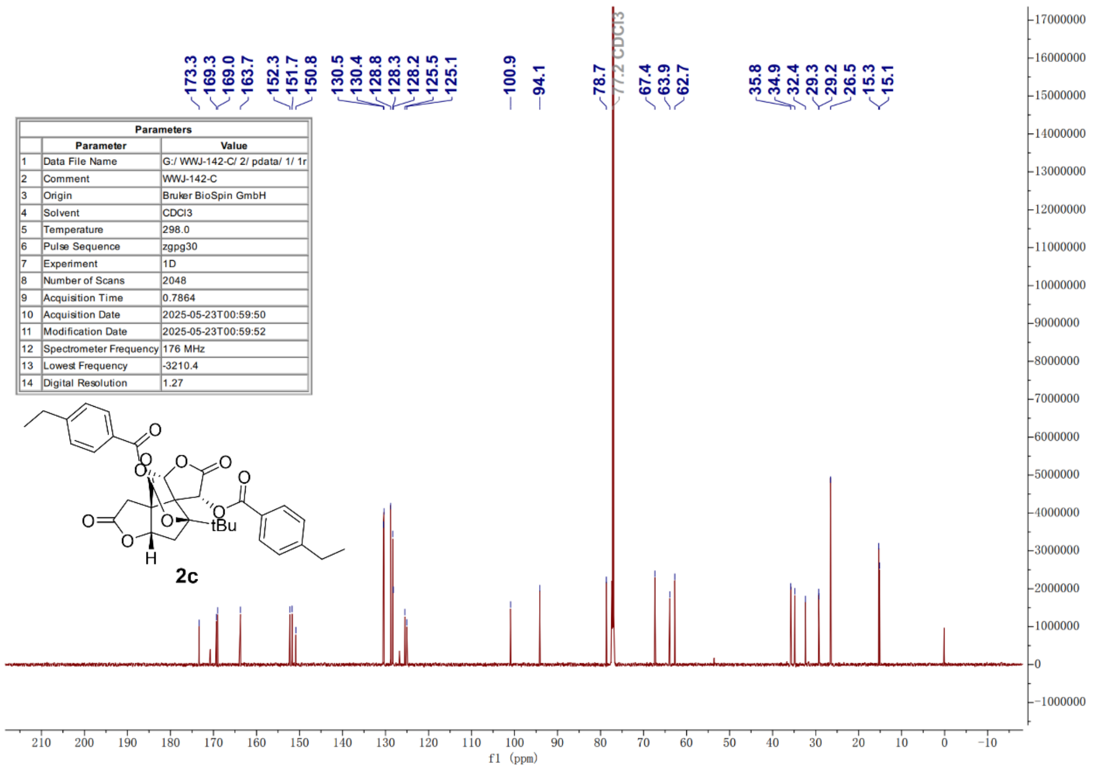

	Parameters	
	Parameter	Value
1	Data File Name	G:/ WWJ-142-C/ 2/ pdata/ 1/ 1r
2	Comment	WWJ-142-C
3	Origin	Bruker BioSpin GmbH
4	Solvent	CDCl3
5	Temperature	298.0
6	Pulse Sequence	zgpg30
7	Experiment	1D
8	Number of Scans	2048
9	Acquisition Time	0.7864
10	Acquisition Date	2025-05-23T00:59:50
11	Modification Date	2025-05-23T00:59:52
12	Spectrometer Frequency	176 MHz
13	Lowest Frequency	-3210.4
14	Digital Resolution	1.27

(2*R*,3a'*S*,4*R*,6'*R*,7a'*S*)-6'-(*tert*-butyl)-2',4',5-trioxohexahydro-4'*H*,6'*H*-spiro[furan-3,8'-[3a,6]methanofuro[3,2-*c*]pyran]-2,4-diyl bis(4-(dimethylamino)benzoate) (2d)

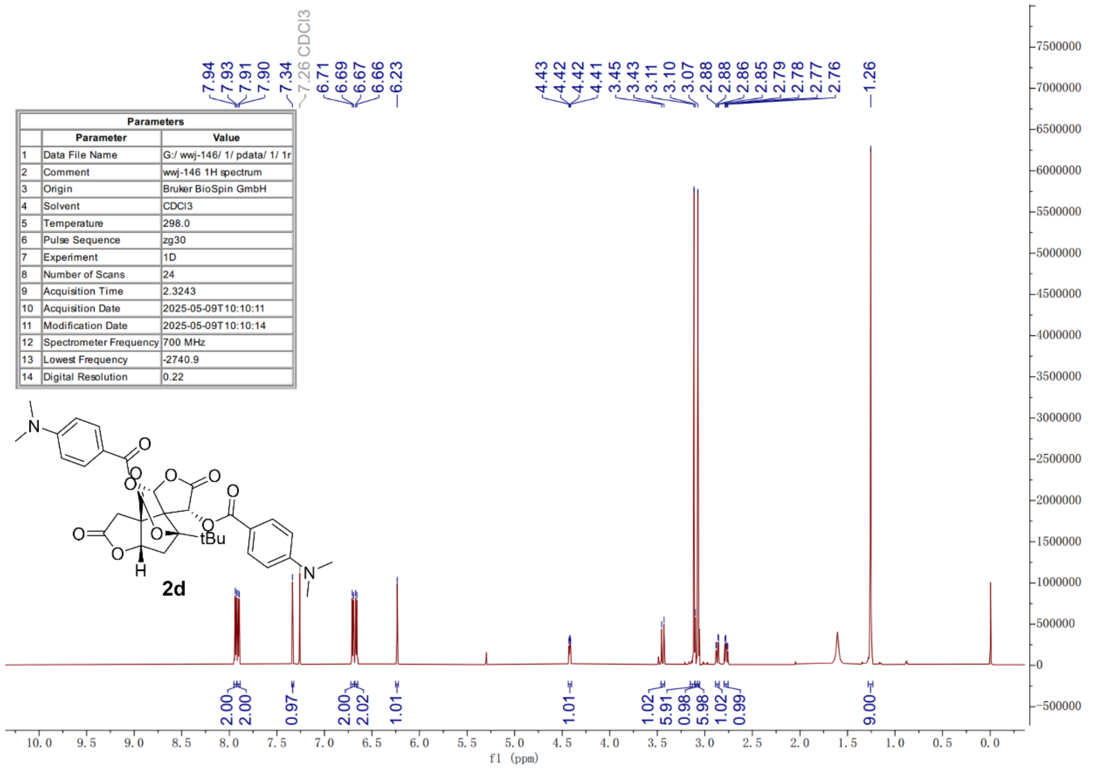

	Parameters	
	Parameter	Value
1	Data File Name	G:/ wwj-146/ 1/ pdata/ 1/ 1r
2	Comment	wwj-146 1H spectrum
3	Origin	Bruker BioSpin GmbH
4	Solvent	CDCl3
5	Temperature	298.0
6	Pulse Sequence	zg30
7	Experiment	1D
8	Number of Scans	24
9	Acquisition Time	2.3243
10	Acquisition Date	2025-05-09T10:10:11
11	Modification Date	2025-05-09T10:10:14
12	Spectrometer Frequency	700 MHz
13	Lowest Frequency	-2740.9
14	Digital Resolution	0.22

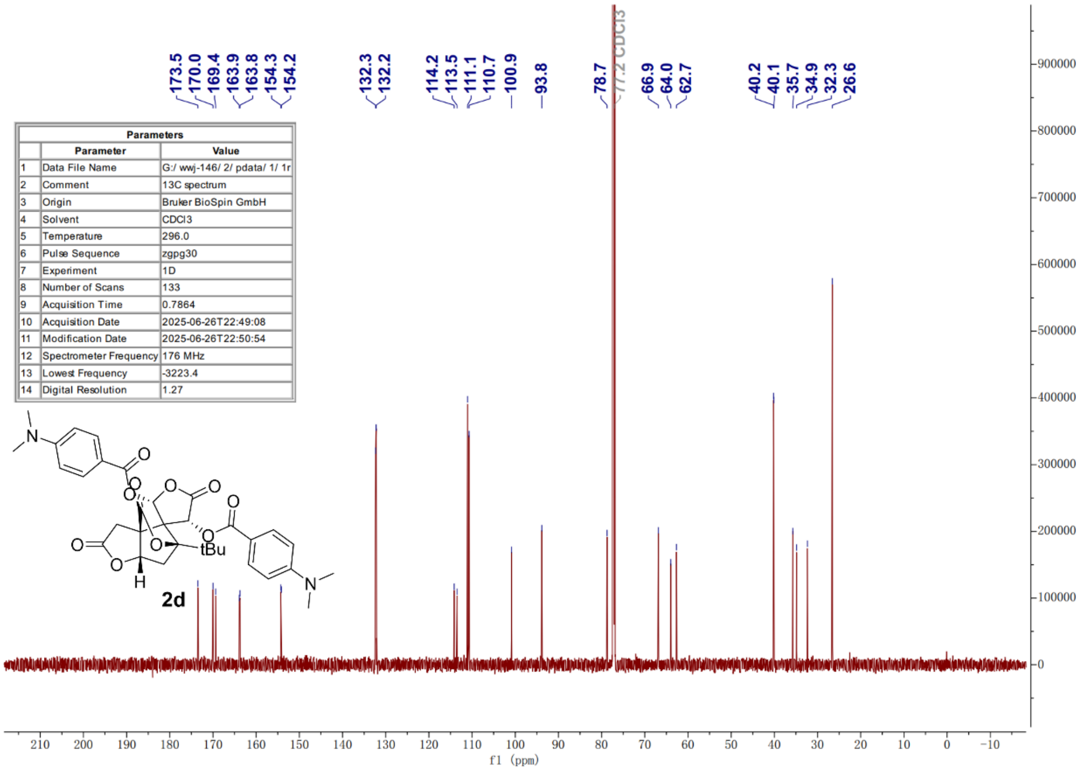

Parameters		
	Parameter	Value
1	Data File Name	G:/ wwj-146/ 2/ pdata/ 1/ 1r
2	Comment	13C spectrum
3	Origin	Bruker BioSpin GmbH
4	Solvent	CDCl3
5	Temperature	296.0
6	Pulse Sequence	zgpg30
7	Experiment	1D
8	Number of Scans	133
9	Acquisition Time	0.7864
10	Acquisition Date	2025-06-26T22:49:08
11	Modification Date	2025-06-26T22:50:54
12	Spectrometer Frequency	176 MHz
13	Lowest Frequency	-3223.4
14	Digital Resolution	1.27

(2*R*,3a'*S*,4*R*,6'*R*,7a'*S*)-6'-(*tert*-butyl)-2',4',5-trioxohexahydro-4'*H*,6'*H*-spiro[furan-3,8'-[3a,6]methanofuro[3,2-*c*]pyran]-2,4-diyl bis(4-fluorobenzoate) (2e)

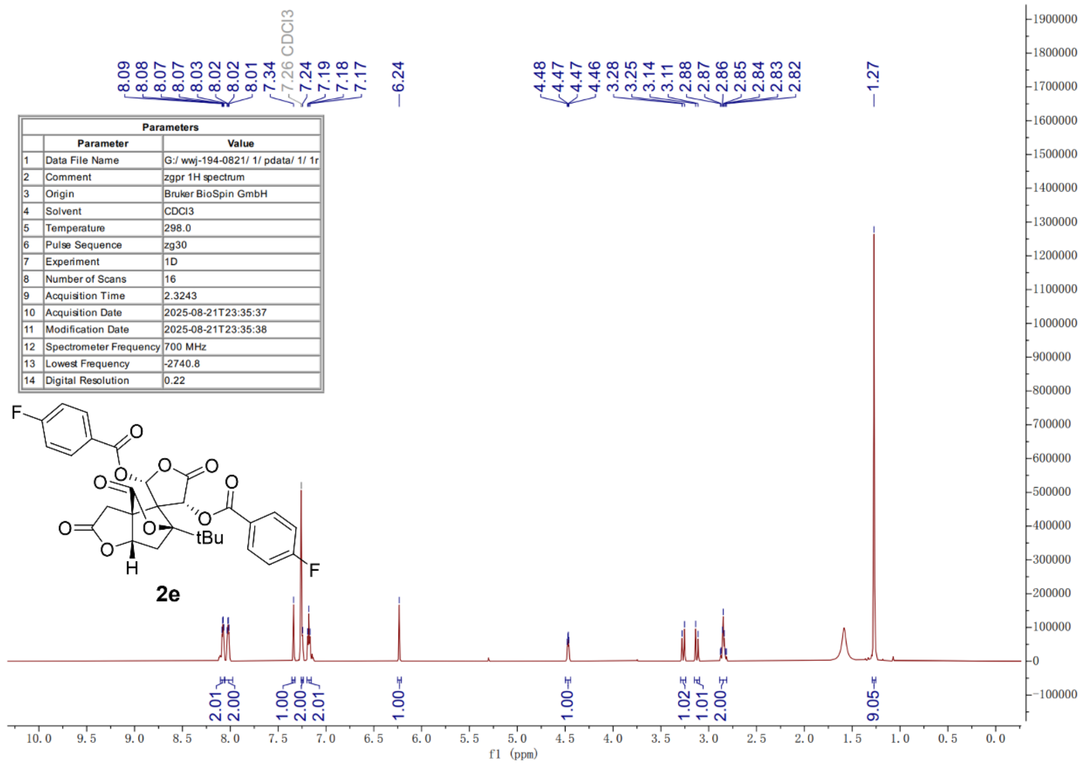

Parameters		
	Parameter	Value
1	Data File Name	G:/ wwj-194-0821/ 1/ pdata/ 1/ 1r
2	Comment	zgpr 1H spectrum
3	Origin	Bruker BioSpin GmbH
4	Solvent	CDCl3
5	Temperature	298.0
6	Pulse Sequence	zg30
7	Experiment	1D
8	Number of Scans	16
9	Acquisition Time	2.3243
10	Acquisition Date	2025-08-21T23:35:37
11	Modification Date	2025-08-21T23:35:38
12	Spectrometer Frequency	700 MHz
13	Lowest Frequency	-2740.8
14	Digital Resolution	0.22

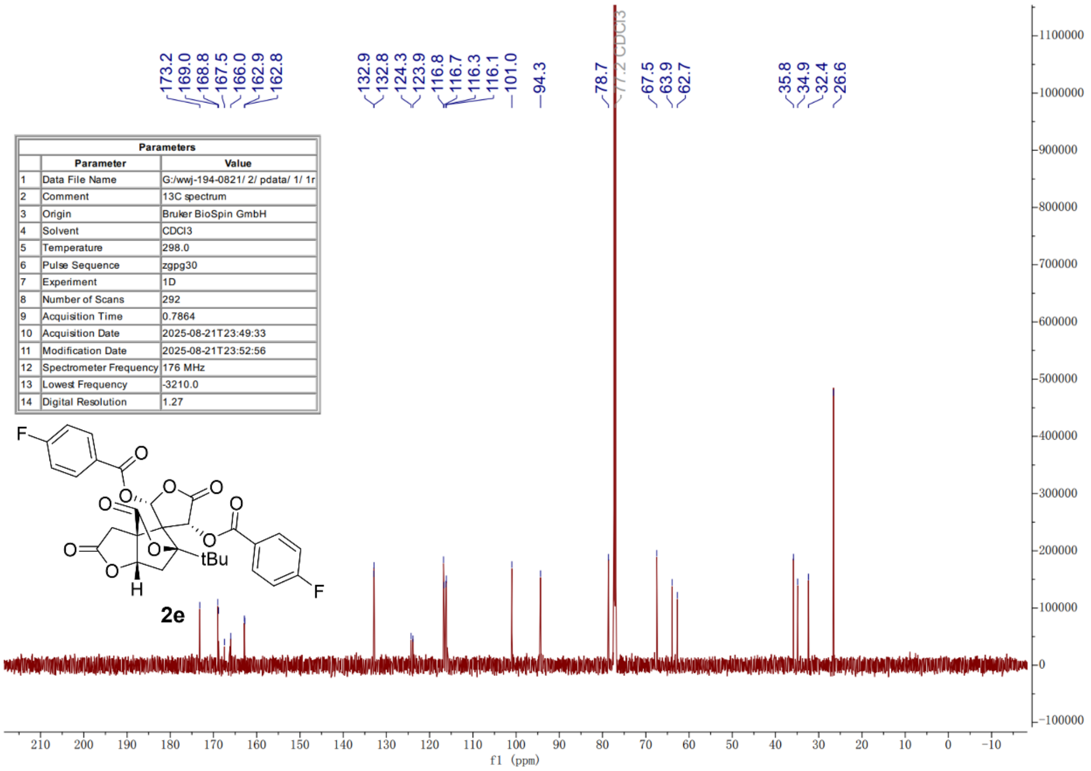

Parameters		
	Parameter	Value
1	Data File Name	G:/wwj-194-0821/ 2/ pdata/ 1/ 1r
2	Comment	13C spectrum
3	Origin	Bruker BioSpin GmbH
4	Solvent	CDCl3
5	Temperature	298.0
6	Pulse Sequence	zgpg30
7	Experiment	1D
8	Number of Scans	292
9	Acquisition Time	0.7864
10	Acquisition Date	2025-08-21T23:49:33
11	Modification Date	2025-08-21T23:52:56
12	Spectrometer Frequency	176 MHz
13	Lowest Frequency	-3210.0
14	Digital Resolution	1.27

(2*R*,3a'*S*,4*R*,6'*R*,7a'*S*)-6'-(*tert*-butyl)-2',4',5-trioxohexahydro-4'*H*,6'*H*-spiro[furan-3,8'-[3a,6]methanofuro[3,2-*c*]pyran]-2,4-diyl bis(2-fluorobenzoate) (2f)

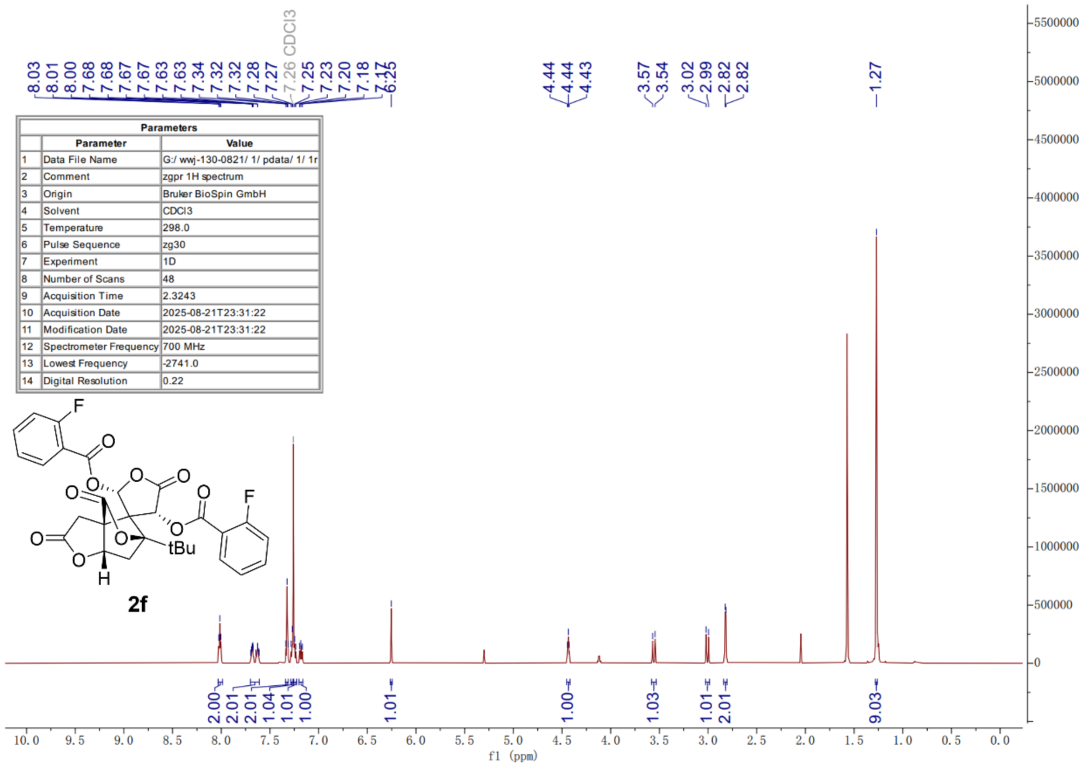

Parameters		
	Parameter	Value
1	Data File Name	G:/ wwj-130-0821/ 1/ pdata/ 1/ 1r
2	Comment	zgpr 1H spectrum
3	Origin	Bruker BioSpin GmbH
4	Solvent	CDCl3
5	Temperature	298.0
6	Pulse Sequence	zg30
7	Experiment	1D
8	Number of Scans	48
9	Acquisition Time	2.3243
10	Acquisition Date	2025-08-21T23:31:22
11	Modification Date	2025-08-21T23:31:22
12	Spectrometer Frequency	700 MHz
13	Lowest Frequency	-2741.0
14	Digital Resolution	0.22

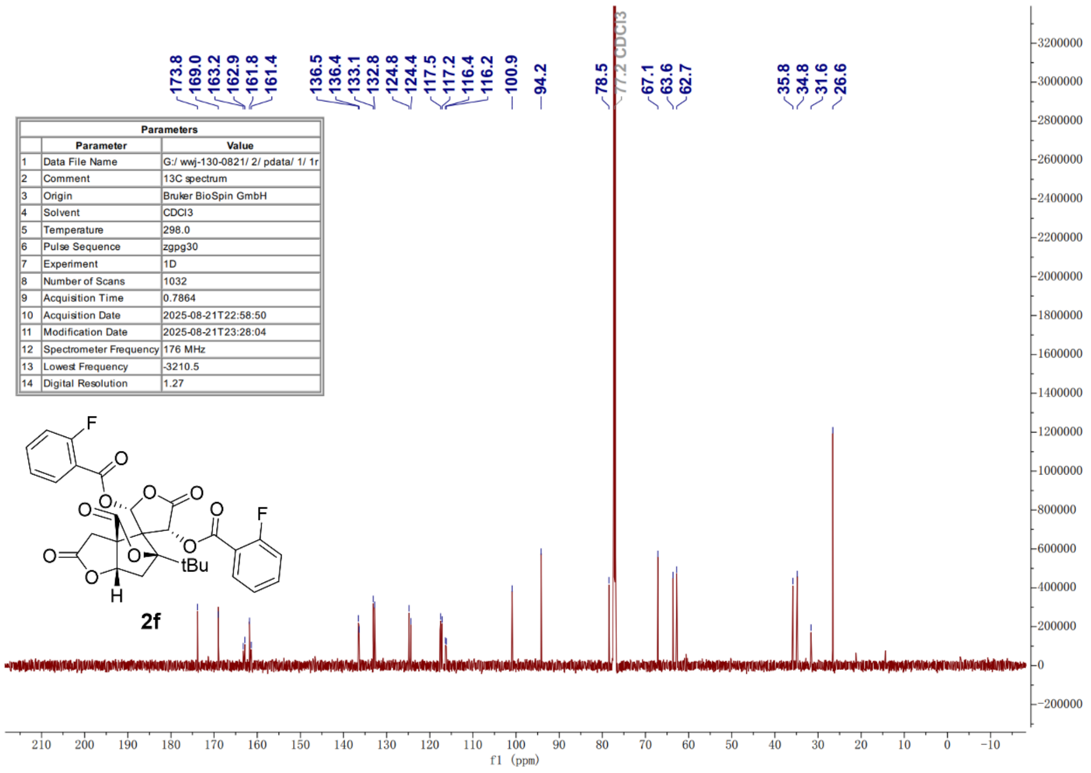

(2*R*,3a'*S*,4*R*,6'*R*,7a'*S*)-6'-(*tert*-butyl)-2',4',5-trioxohexahydro-4'*H*,6'*H*-spiro[furan-3,8'-[3a,6]methanofuro[3,2-*c*]pyran]-2,4-diyl bis(2-(trifluoromethyl)benzoate) (2g)

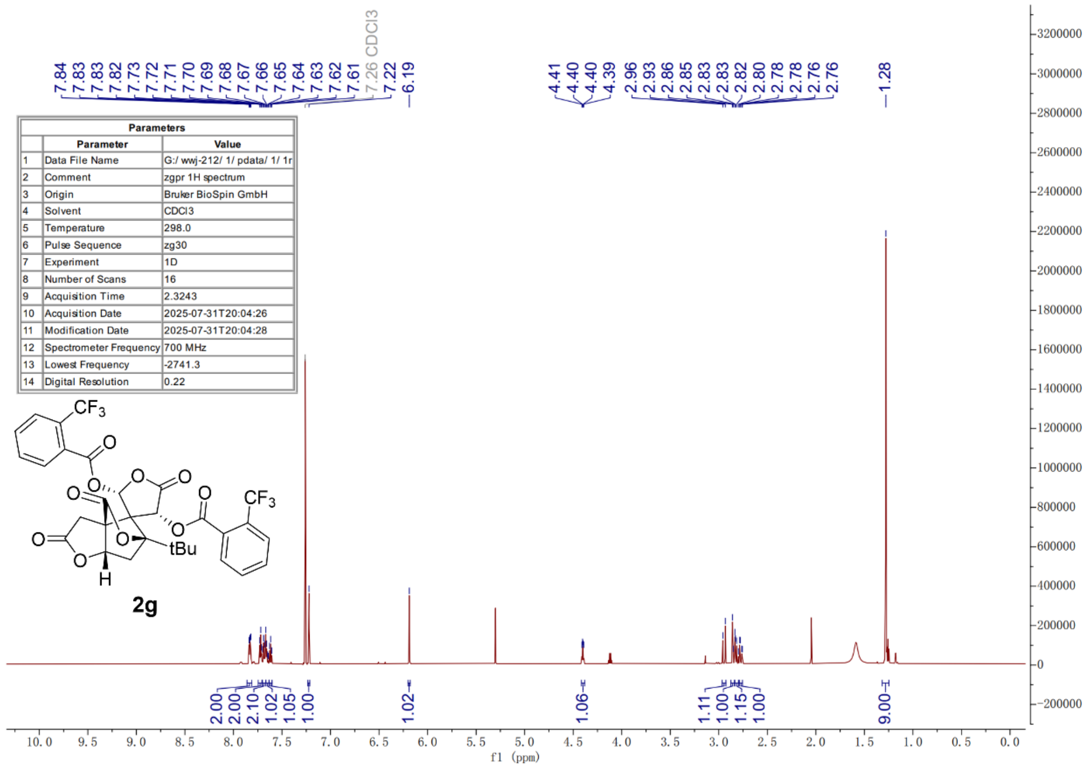

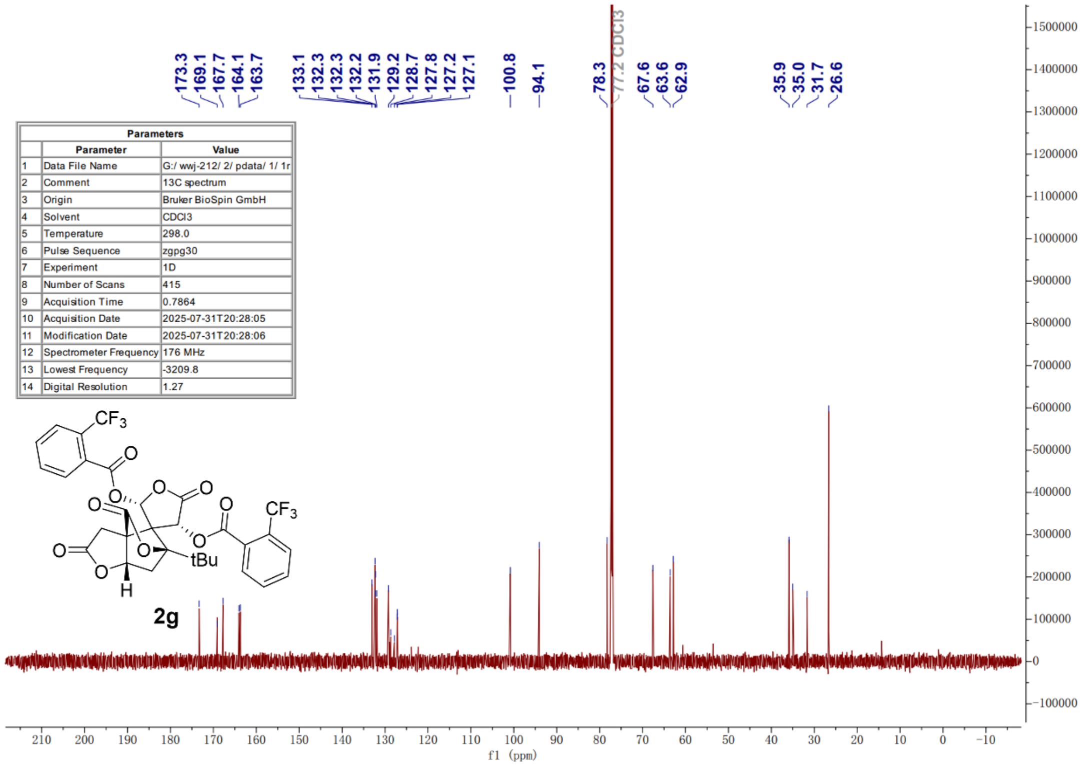

(2*R*,3a'*S*,4*R*,6'*R*,7a'*S*)-6'-(*tert*-butyl)-2',4',5-trioxohexahydro-4'*H*,6'*H*-spiro[furan-3,8'-[3a,6]methanofuro[3,2-*c*]pyran]-2,4-diyl bis(3-fluorobenzoate) (2h)

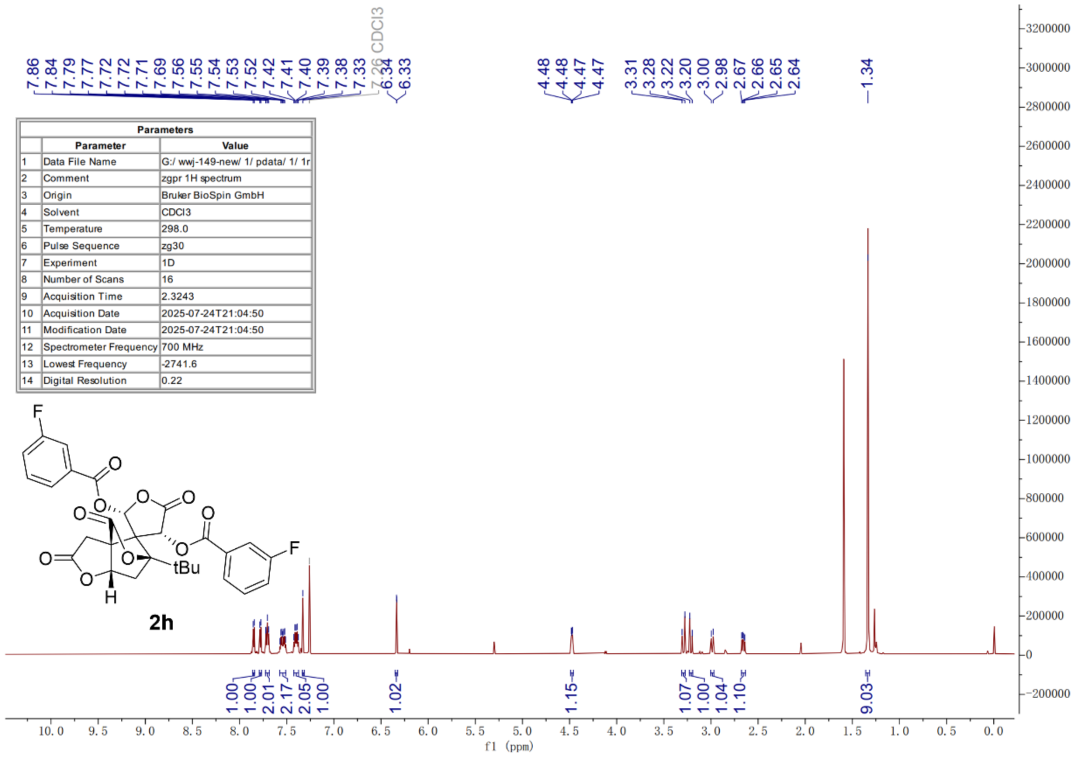

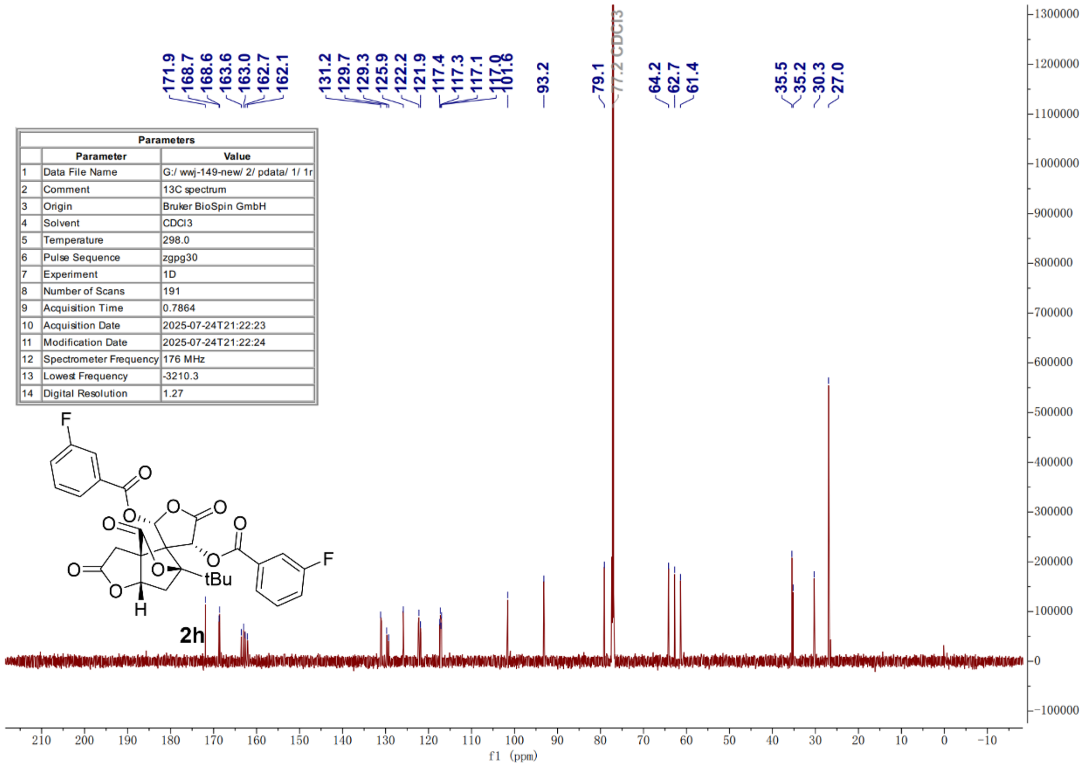

Parameters		
	Parameter	Value
1	Data File Name	G:/ wwj-149-new/ 2/ pdata/ 1/ 1r
2	Comment	13C spectrum
3	Origin	Bruker BioSpin GmbH
4	Solvent	CDCl3
5	Temperature	298.0
6	Pulse Sequence	zgpg30
7	Experiment	1D
8	Number of Scans	191
9	Acquisition Time	0.7864
10	Acquisition Date	2025-07-24T21:22:23
11	Modification Date	2025-07-24T21:22:24
12	Spectrometer Frequency	176 MHz
13	Lowest Frequency	-3210.3
14	Digital Resolution	1.27

(2*R*,3a'*S*,4*R*,6'*R*,7a'*S*)-6'-(*tert*-butyl)-2',4',5-trioxohexahydro-4'*H*,6'*H*-spiro[furan-3,8'-[3a,6]methanofuro[3,2-*c*]pyran]-2,4-diyl bis(3-(trifluoromethyl)benzoate) (2i)

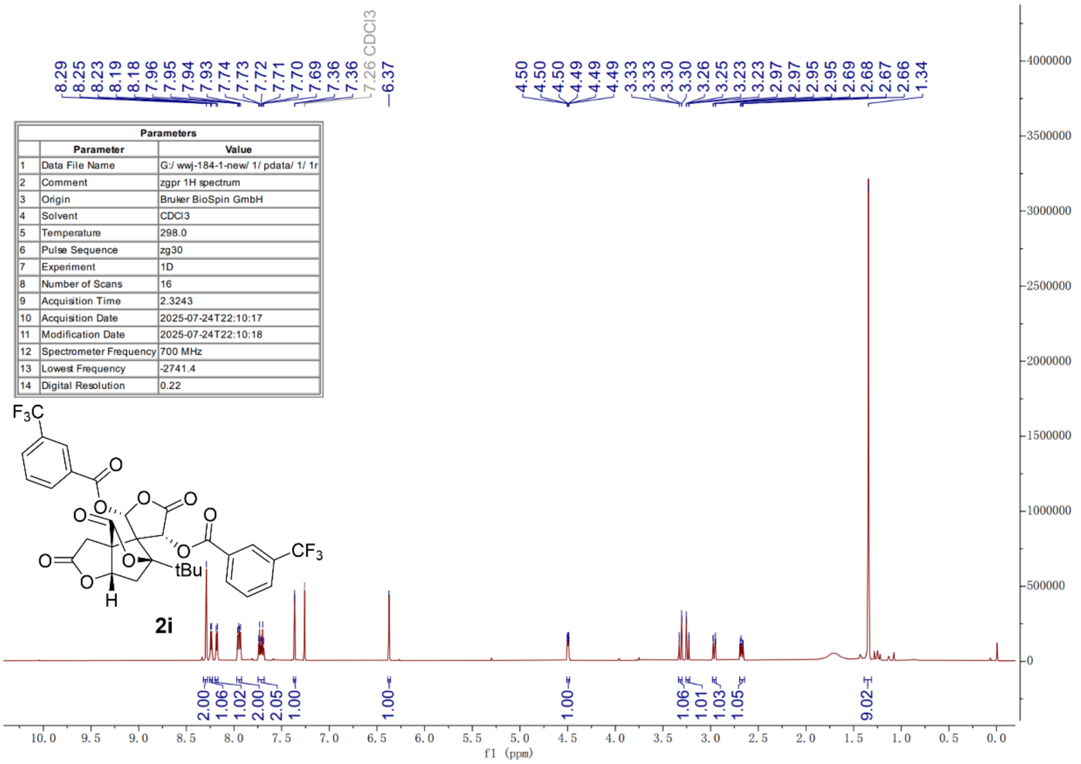

Parameters		
	Parameter	Value
1	Data File Name	G:/ wwj-184-1-new/ 1/ pdata/ 1/ 1r
2	Comment	zgpr 1H spectrum
3	Origin	Bruker BioSpin GmbH
4	Solvent	CDCl3
5	Temperature	298.0
6	Pulse Sequence	zg30
7	Experiment	1D
8	Number of Scans	16
9	Acquisition Time	2.3243
10	Acquisition Date	2025-07-24T22:10:17
11	Modification Date	2025-07-24T22:10:18
12	Spectrometer Frequency	700 MHz
13	Lowest Frequency	-2741.4
14	Digital Resolution	0.22

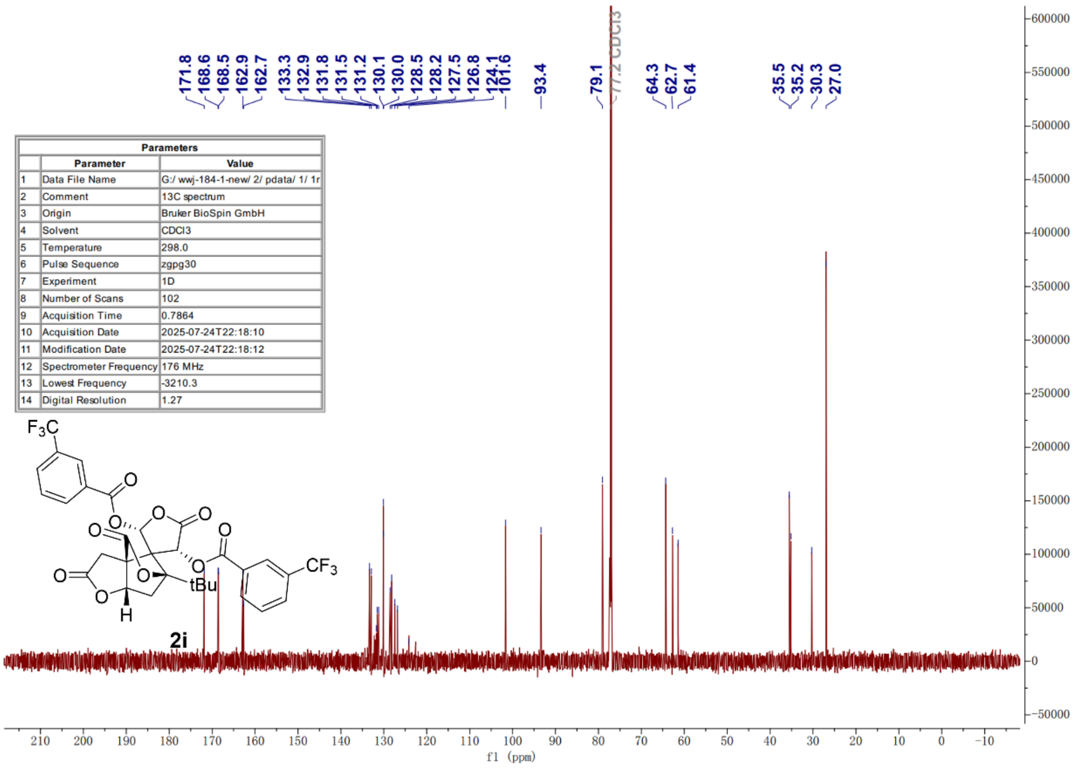

	Parameter	Value
1	Data File Name	G:/ wwj-184-1-new/ 2/ pdata/ 1/ 1r
2	Comment	13C spectrum
3	Origin	Bruker BioSpin GmbH
4	Solvent	CDCl3
5	Temperature	298.0
6	Pulse Sequence	zgpg30
7	Experiment	1D
8	Number of Scans	102
9	Acquisition Time	0.7864
10	Acquisition Date	2025-07-24T22:18:10
11	Modification Date	2025-07-24T22:18:12
12	Spectrometer Frequency	176 MHz
13	Lowest Frequency	-3210.3
14	Digital Resolution	1.27

6.2. NMR spectra of C-Ring Modification 5a-5k

(3a*S*,5a*R*,8*R*,9*R*,10a*S*)-5-benzyl-9-(*tert*-butyl)-9-hydroxy-2,4,7-trioxooctahydro-4*H*,9*H*-furo[2,3-*b*]furo[3',2':2,3]cyclopenta[1,2-*c*]pyrrol-8-yl benzoate (5a)

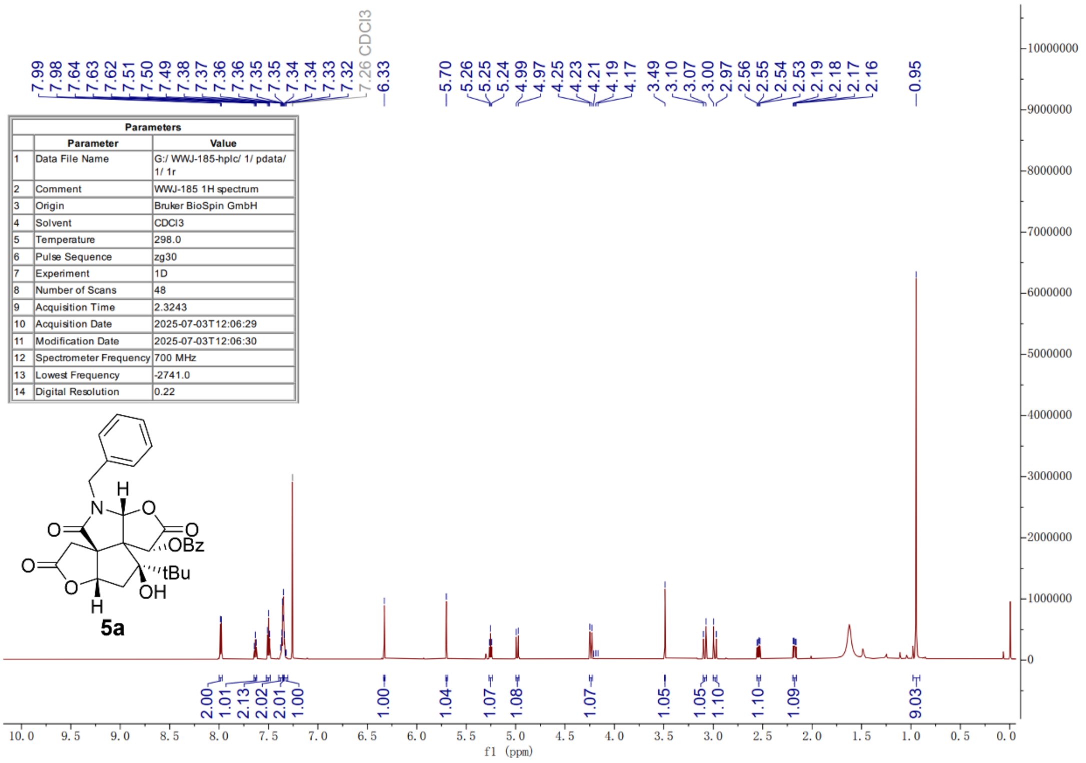

	Parameter	Value
1	Data File Name	G:/ WWJ-185-hplc/ 1/ pdata/ 1/ 1r
2	Comment	WWJ-185 1H spectrum
3	Origin	Bruker BioSpin GmbH
4	Solvent	CDCl3
5	Temperature	298.0
6	Pulse Sequence	zg30
7	Experiment	1D
8	Number of Scans	48
9	Acquisition Time	2.3243
10	Acquisition Date	2025-07-03T12:06:29
11	Modification Date	2025-07-03T12:06:30
12	Spectrometer Frequency	700 MHz
13	Lowest Frequency	-2741.0
14	Digital Resolution	0.22

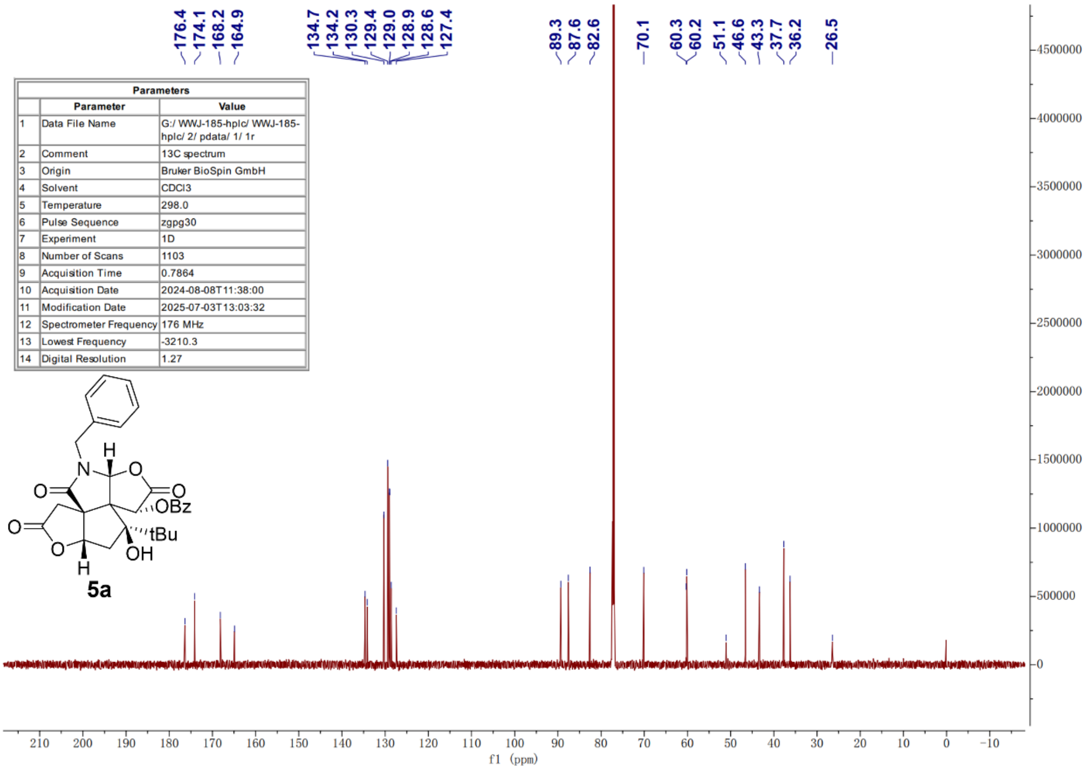

Parameters		
	Parameter	Value
1	Data File Name	G:/ WWJ-185-hplc/ WWJ-185-hplc/ 2/ pdata/ 1/ 1r
2	Comment	13C spectrum
3	Origin	Bruker BioSpin GmbH
4	Solvent	CDCl3
5	Temperature	298.0
6	Pulse Sequence	zgpg30
7	Experiment	1D
8	Number of Scans	1103
9	Acquisition Time	0.7864
10	Acquisition Date	2024-08-08T11:38:00
11	Modification Date	2025-07-03T13:03:32
12	Spectrometer Frequency	176 MHz
13	Lowest Frequency	-3210.3
14	Digital Resolution	1.27

Parameters		
	Parameter	Value
1	Data File Name	C:/ Users/ Wenji/ Downloads/ SCC539_600/ SCC539_600/ 3/ pdata/ 1/ 2rr
2	Comment	HMBC
3	Origin	Bruker BioSpin GmbH
4	Solvent	CD2Cl2
5	Temperature	298.7
6	Pulse Sequence	hmbcgplpndqf
7	Experiment	HMBC
8	Number of Scans	6
9	Acquisition Time	0.1420
10	Acquisition Date	2023-09-14T18:05:15
11	Modification Date	2026-03-15T23:36:22
12	Spectrometer Frequency	600 MHz, 151 MHz
13	Lowest Frequency	(236.9, -1219.6)
14	Digital Resolution	(3.52, 32.34)

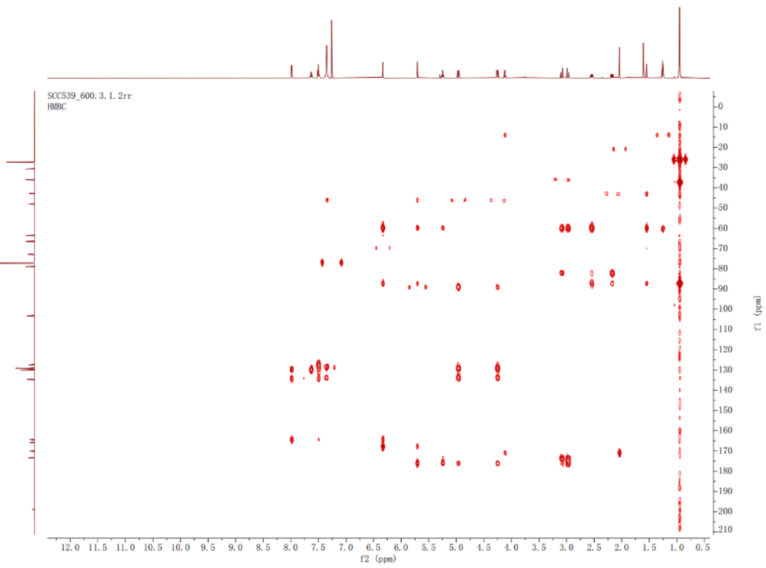

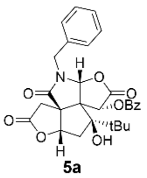

5a

Parameters		
	Parameter	Value
1	Data File Name	C:/ Users/ Wenji/ Downloads/ SCC539_600/ SCC539_600/ 2/ pdata/ 1/ 2rr
2	Comment	HSQC
3	Origin	Bruker BioSpin GmbH
4	Solvent	CD2Cl2
5	Temperature	298.8
6	Pulse Sequence	hsqcetgpsi2
7	Experiment	HSQC
8	Number of Scans	4
9	Acquisition Time	0.0655
10	Acquisition Date	2023-09-14T17:50:22
11	Modification Date	2026-03-15T23:36:22
12	Spectrometer Frequency	600 MHz, 151 MHz
13	Lowest Frequency	(-1264.8, -5749.9)
14	Digital Resolution	(7.63, 32.34)

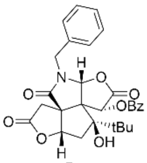

5a

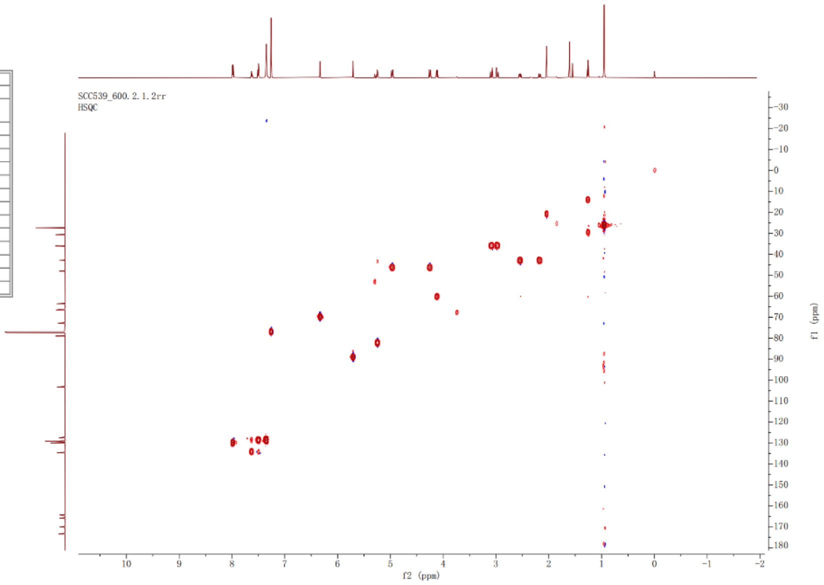

Parameters		
	Parameter	Value
1	Data File Name	C:/ Users/ Wenji/ Downloads/ SCC539_600/ SCC539_600/ 4/ pdata/ 1/ 2rr
2	Comment	NOESY
3	Origin	Bruker BioSpin GmbH
4	Solvent	CD2Cl2
5	Temperature	298.6
6	Pulse Sequence	noesygpph
7	Experiment	NOESY
8	Number of Scans	4
9	Acquisition Time	0.1420
10	Acquisition Date	2023-09-14T18:30:17
11	Modification Date	2026-03-15T23:36:23
12	Spectrometer Frequency	600 MHz, 600 MHZ
13	Lowest Frequency	(-66.1, -60.4)
14	Digital Resolution	(7.04, 7.04)

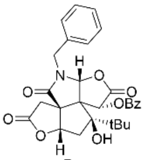

5a

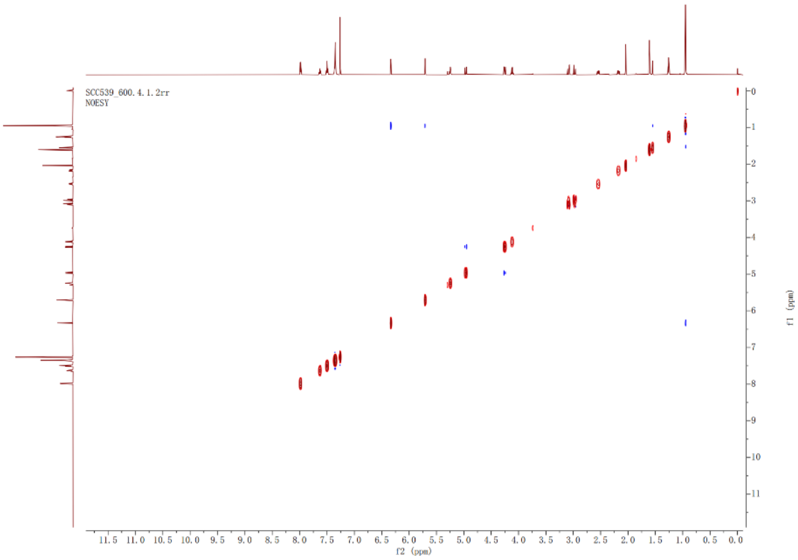

(3a*S*,5a*R*,8*R*,9*R*,10a*S*)-9-(*tert*-butyl)-9-hydroxy-5-(4-methoxybenzyl)-2,4,7-trioxooctahydro-4*H*,9*H*-furo[2,3-*b*]furo[3',2':2,3]cyclopenta[1,2-*c*]pyrrol-8-yl benzoate (5b)

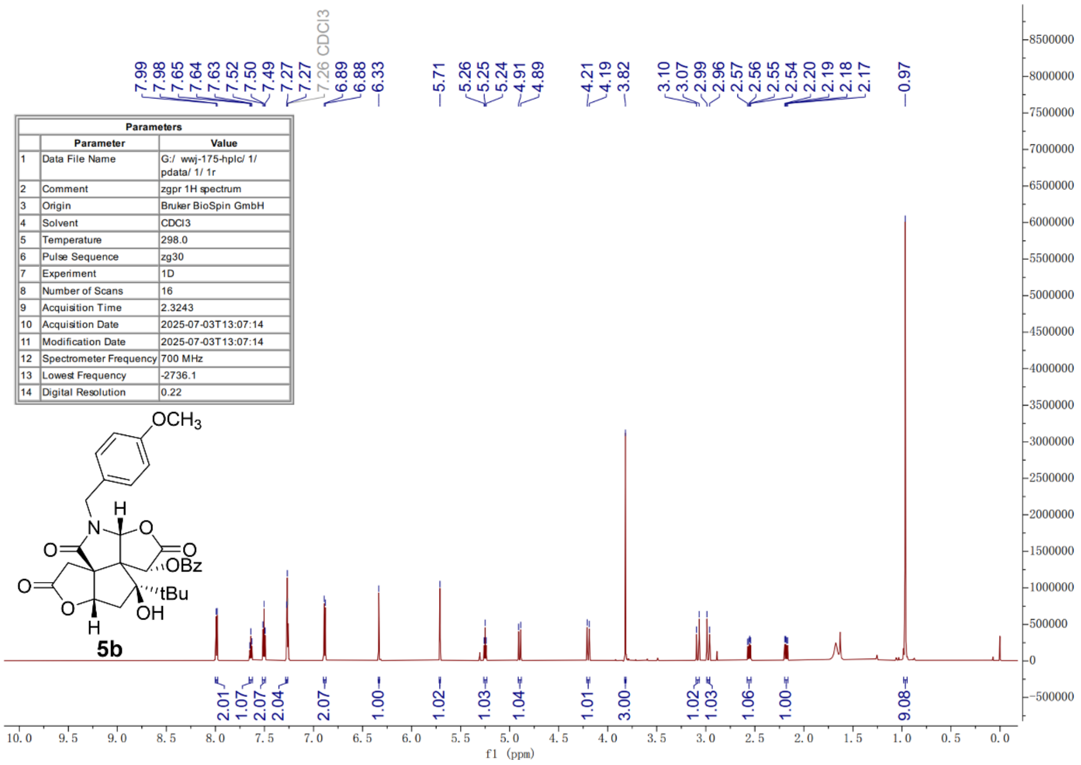

Parameters		
	Parameter	Value
1	Data File Name	G:/ wwj-175-hplc/ 1/ pdata/ 1/ 1r
2	Comment	zgpr 1H spectrum
3	Origin	Bruker BioSpin GmbH
4	Solvent	CDCl3
5	Temperature	298.0
6	Pulse Sequence	zg30
7	Experiment	1D
8	Number of Scans	16
9	Acquisition Time	2.3243
10	Acquisition Date	2025-07-03T13:07:14
11	Modification Date	2025-07-03T13:07:14
12	Spectrometer Frequency	700 MHz
13	Lowest Frequency	-2736.1
14	Digital Resolution	0.22

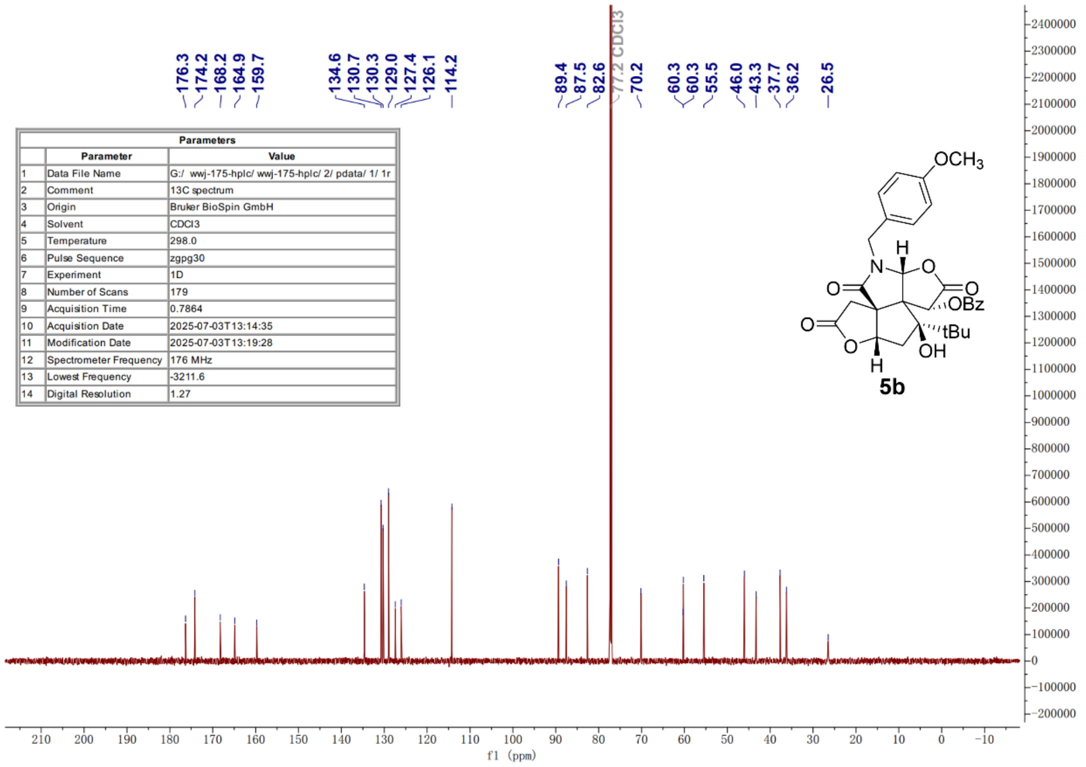

Parameters		
	Parameter	Value
1	Data File Name	G:/ wwj-175-hplc/ wwj-175-hplc/ 2/ pdata/ 1/ 1r
2	Comment	13C spectrum
3	Origin	Bruker BioSpin GmbH
4	Solvent	CDCl3
5	Temperature	298.0
6	Pulse Sequence	zgpg30
7	Experiment	1D
8	Number of Scans	179
9	Acquisition Time	0.7864
10	Acquisition Date	2025-07-03T13:14:35
11	Modification Date	2025-07-03T13:19:28
12	Spectrometer Frequency	176 MHz
13	Lowest Frequency	-3211.6
14	Digital Resolution	1.27

(3a*S*,5a*R*,8*R*,9*R*,10a*S*)-9-(*tert*-butyl)-5-(2,4-dimethoxybenzyl)-9-hydroxy-2,4,7-trioxooctahydro-4*H*,9*H*-furo[2,3-*b*]furo[3',2':2,3]cyclopenta[1,2-*c*]pyrrol-8-yl benzoate (5c)

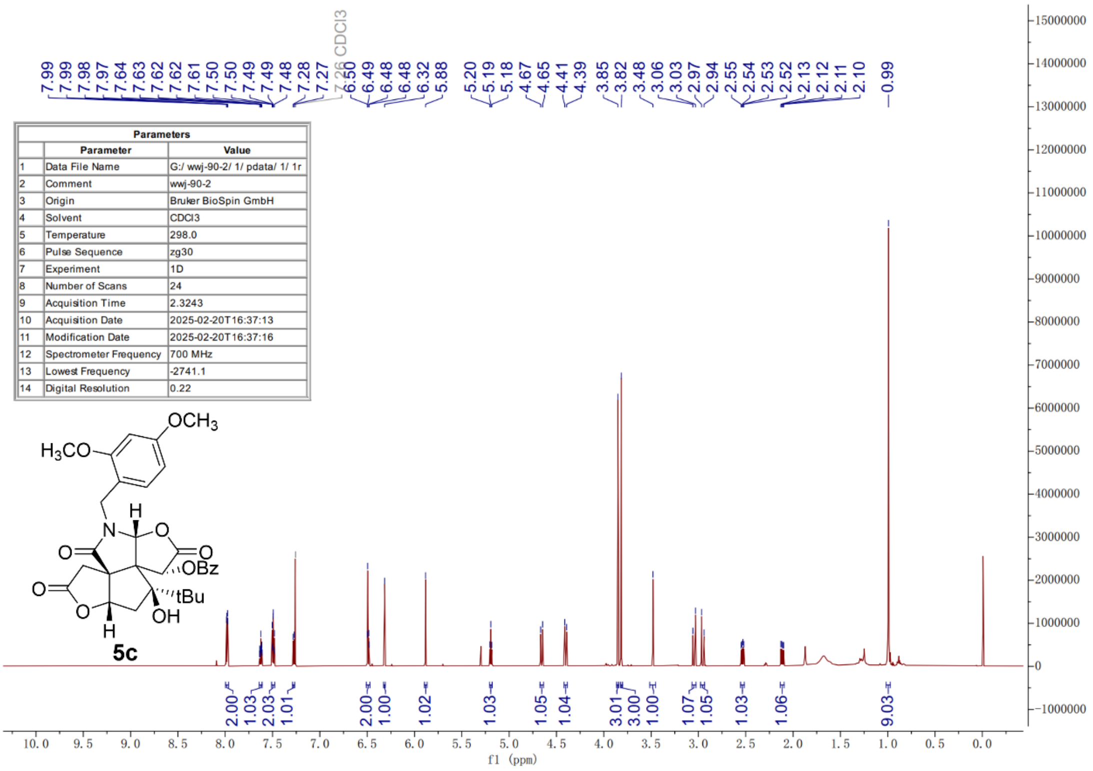

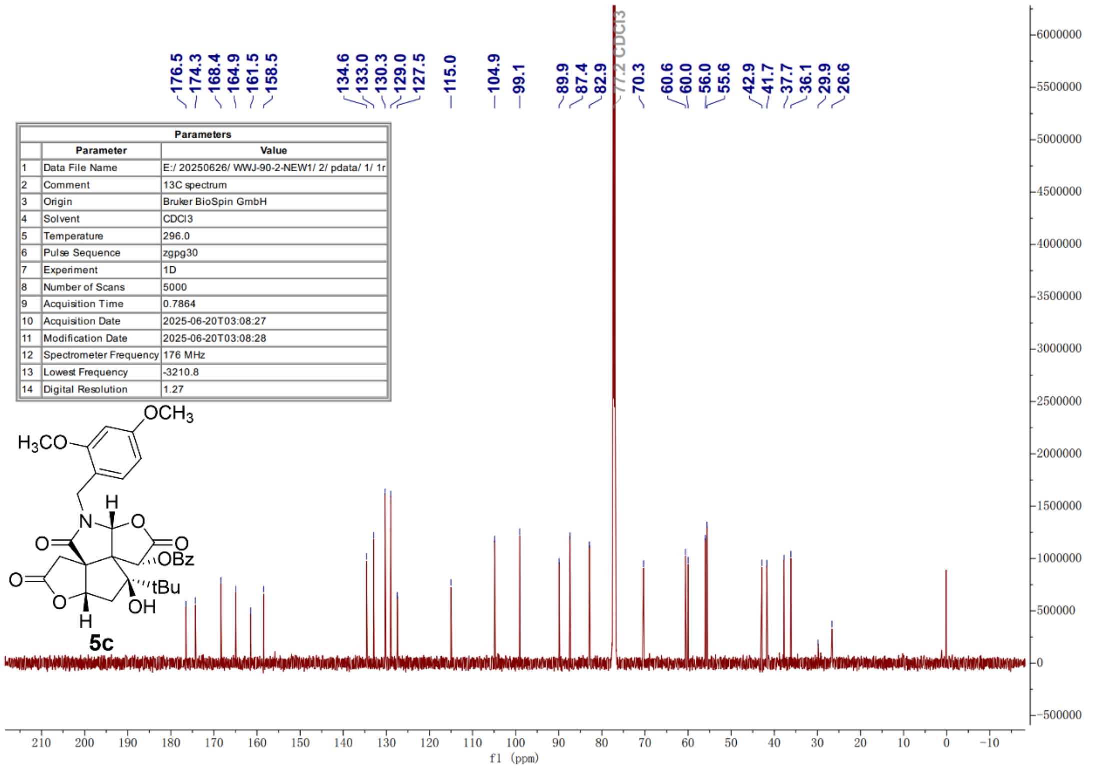

(3a*S*,5a*R*,8*R*,9*R*,10a*S*)-5-(((1*R*,3*R*,5*S*)-adamantan-1-yl)methyl)-9-(*tert*-butyl)-9-hydroxy-2,4,7-trioxooctahydro-4*H*,9*H*-furo[2,3-*b*]furo[3',2':2,3]cyclopenta[1,2-*c*]pyrrol-8-yl benzoate (5d)

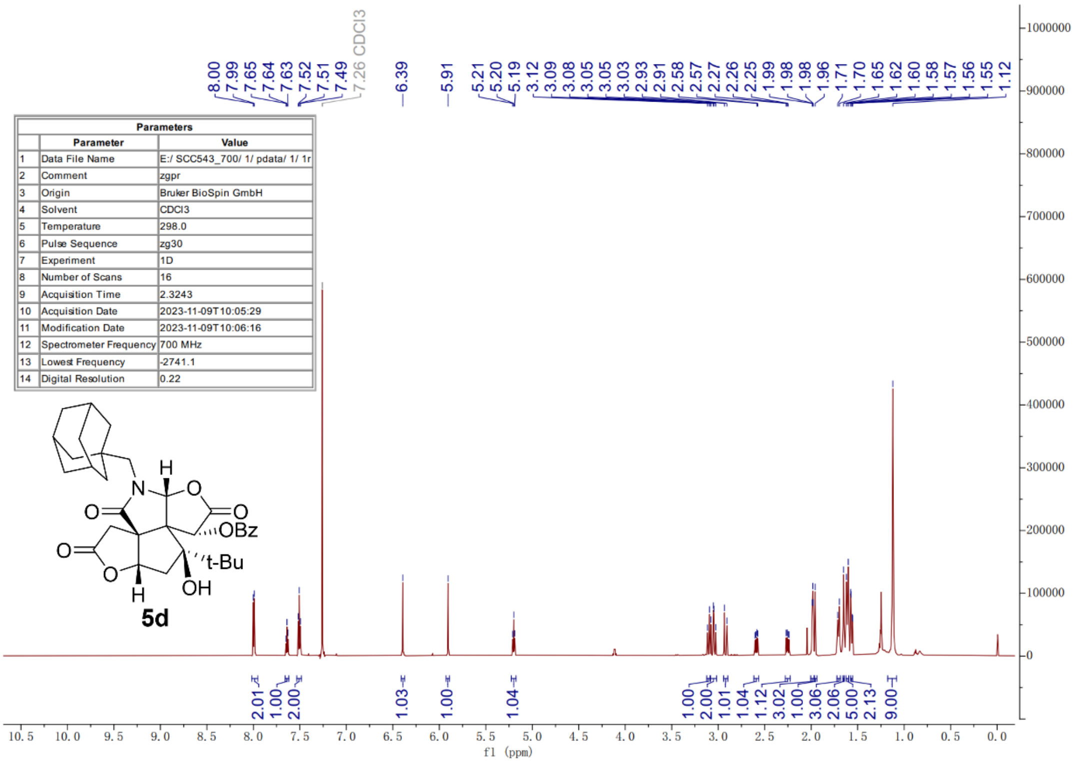

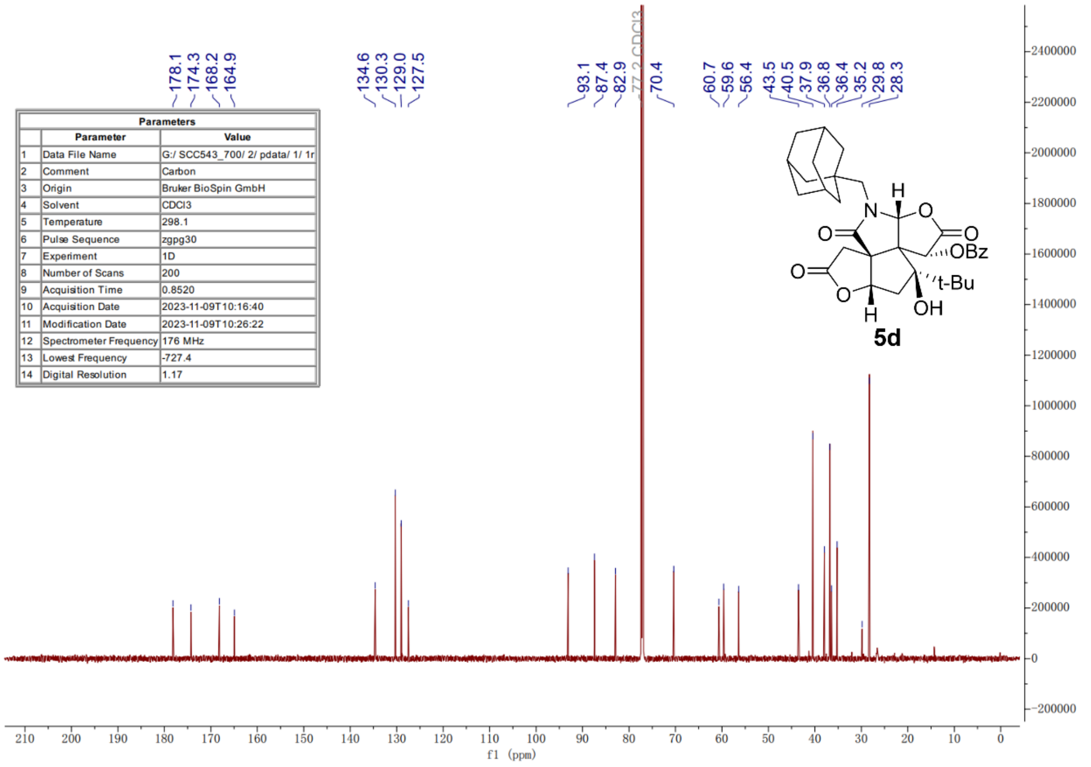

(3a*S*,5a*R*,8*R*,8a*S*,9*R*,10a*S*)-9-(*tert*-butyl)-5-(cyclohexylmethyl)-9-hydroxy-2,4,7-trioxooctahydro-4*H*,9*H*-furo[2,3-*b*]furo[3',2':2,3]cyclopenta[1,2-*c*]pyrrol-8-yl benzoate (5e)

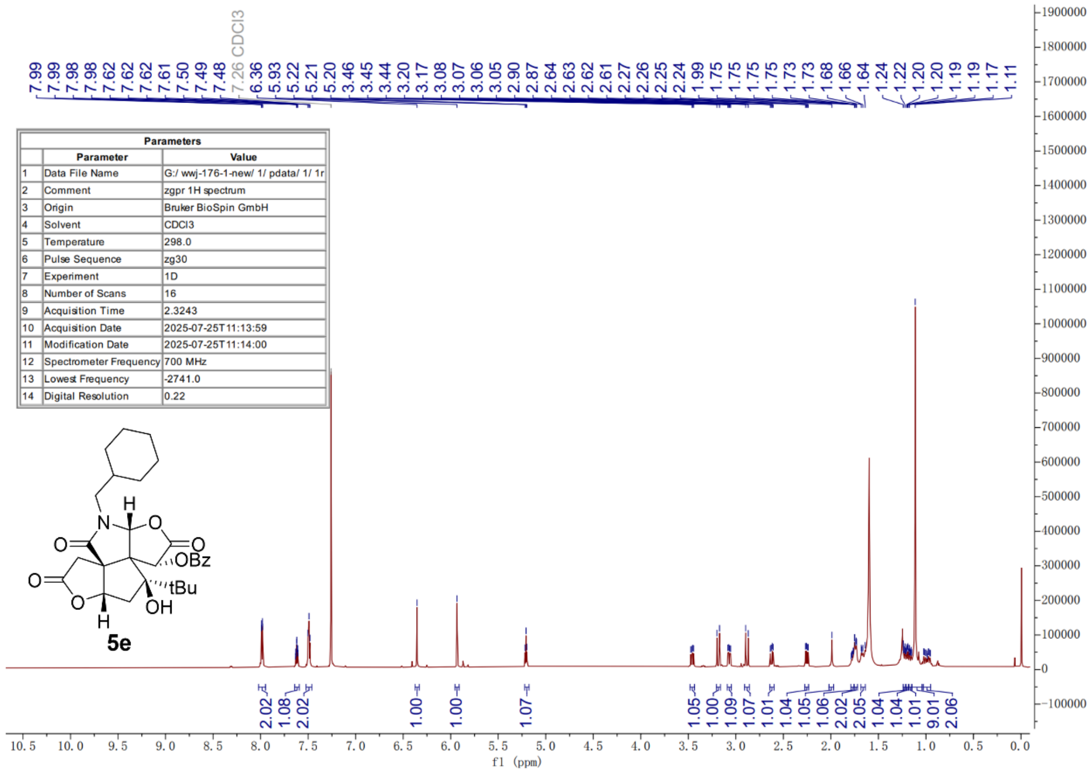

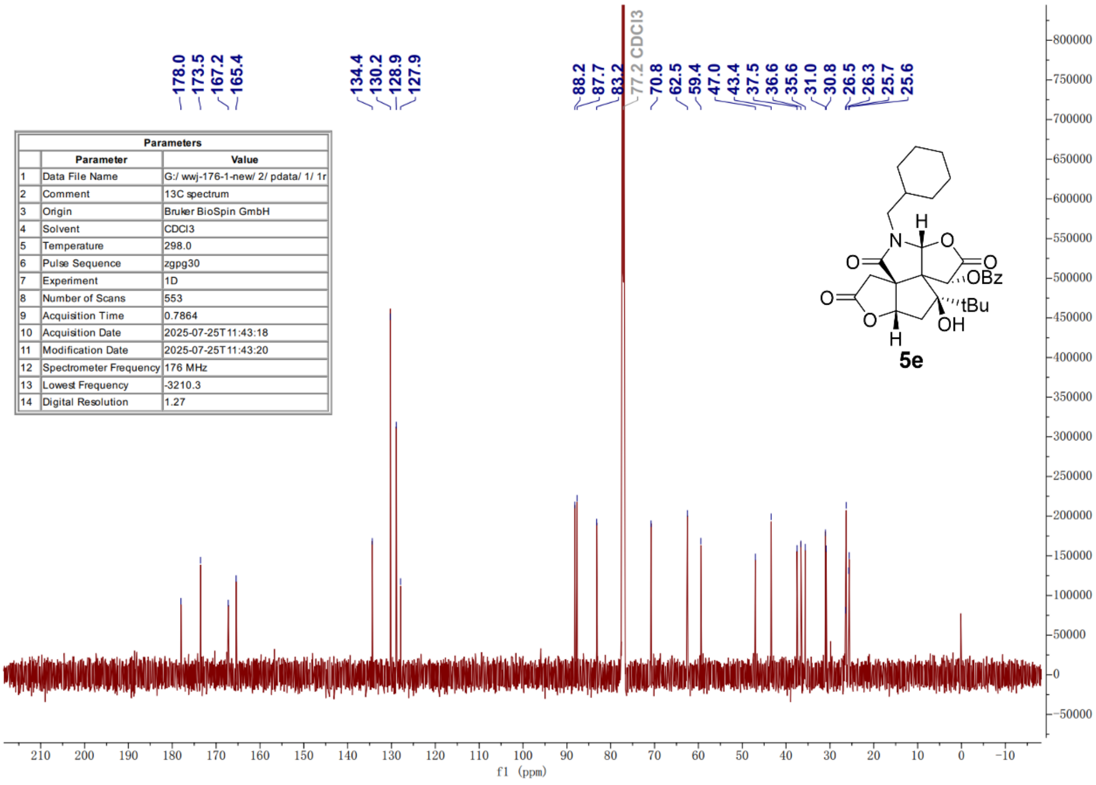

(3a*S*,5a*R*,8*R*,8a*S*,9*R*,10a*S*)-9-(*tert*-butyl)-9-hydroxy-5-neopentyl-2,4,7-trioxooctahydro-4*H*,9*H*-furo[2,3-*b*]furo[3',2':2,3]cyclopenta[1,2-*c*]pyrrol-8-yl benzoate (5f)

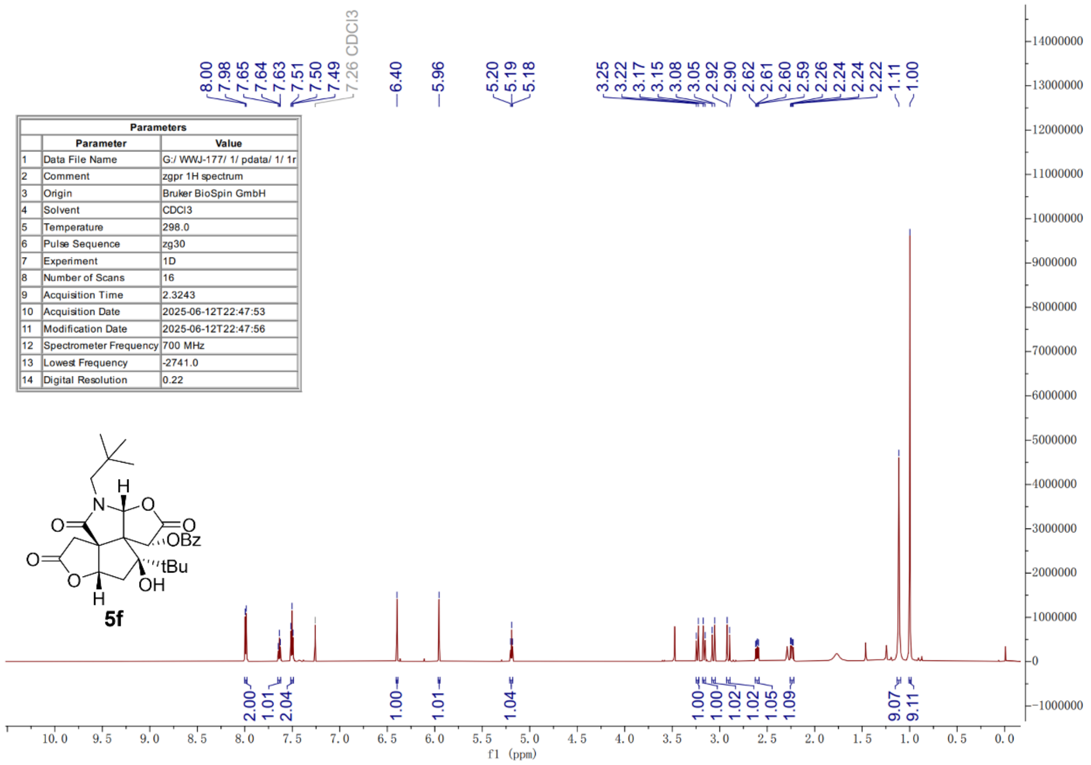

Parameters	
Parameter	Value
1 Data File Name	G:/ WWJ-177/ 1/ pdata/ 1/ 1r
2 Comment	zgpr 1H spectrum
3 Origin	Bruker BioSpin GmbH
4 Solvent	CDCl3
5 Temperature	298.0
6 Pulse Sequence	zg30
7 Experiment	1D
8 Number of Scans	16
9 Acquisition Time	2.3243
10 Acquisition Date	2025-06-12T22:47:53
11 Modification Date	2025-06-12T22:47:56
12 Spectrometer Frequency	700 MHz
13 Lowest Frequency	-2741.0
14 Digital Resolution	0.22

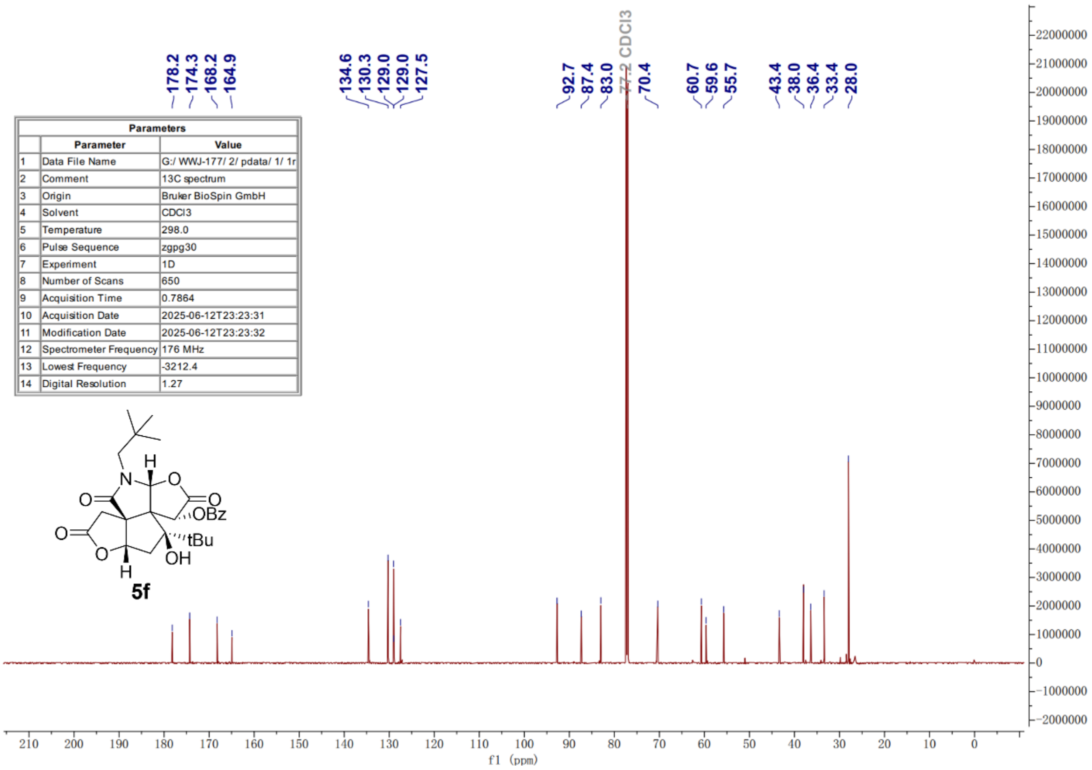

Parameters	
Parameter	Value
1 Data File Name	G:/ WWJ-177/ 2/ pdata/ 1/ 1r
2 Comment	13C spectrum
3 Origin	Bruker BioSpin GmbH
4 Solvent	CDCl3
5 Temperature	298.0
6 Pulse Sequence	zgpg30
7 Experiment	1D
8 Number of Scans	650
9 Acquisition Time	0.7864
10 Acquisition Date	2025-06-12T23:23:31
11 Modification Date	2025-06-12T23:23:32
12 Spectrometer Frequency	176 MHz
13 Lowest Frequency	-3212.4
14 Digital Resolution	1.27

(3a*S*,5a*R*,8*R*,9*R*,10a*S*)-9-(*tert*-butyl)-9-hydroxy-5-(3-hydroxy-4-methoxyphenethyl)-2,4,7-trioxooctahydro-4*H*,9*H*-furo[2,3-*b*]furo[3',2':2,3]cyclopenta[1,2-*c*]pyrrol-8-yl benzoate (5g)

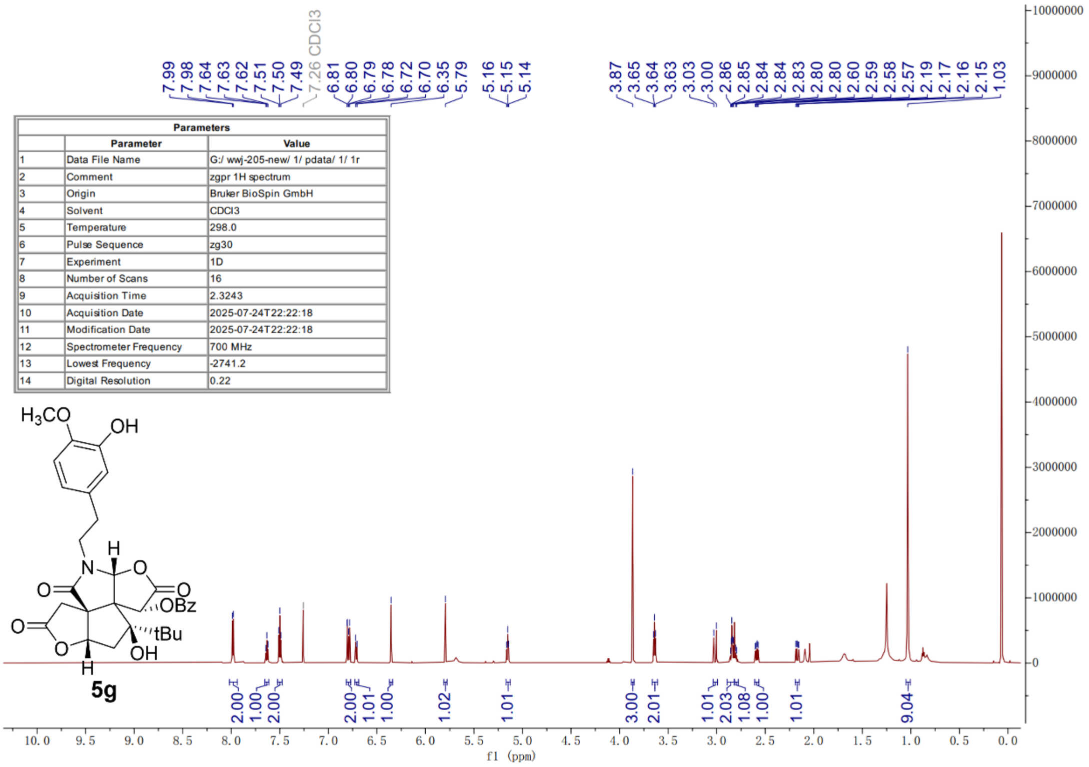

	Parameter	Value
1	Data File Name	G:/ wwj-205-new/ 1/ pdata/ 1/ 1r
2	Comment	zgpr 1H spectrum
3	Origin	Bruker BioSpin GmbH
4	Solvent	CDCl3
5	Temperature	298.0
6	Pulse Sequence	zg30
7	Experiment	1D
8	Number of Scans	16
9	Acquisition Time	2.3243
10	Acquisition Date	2025-07-24T22:22:18
11	Modification Date	2025-07-24T22:22:18
12	Spectrometer Frequency	700 MHz
13	Lowest Frequency	-2741.2
14	Digital Resolution	0.22

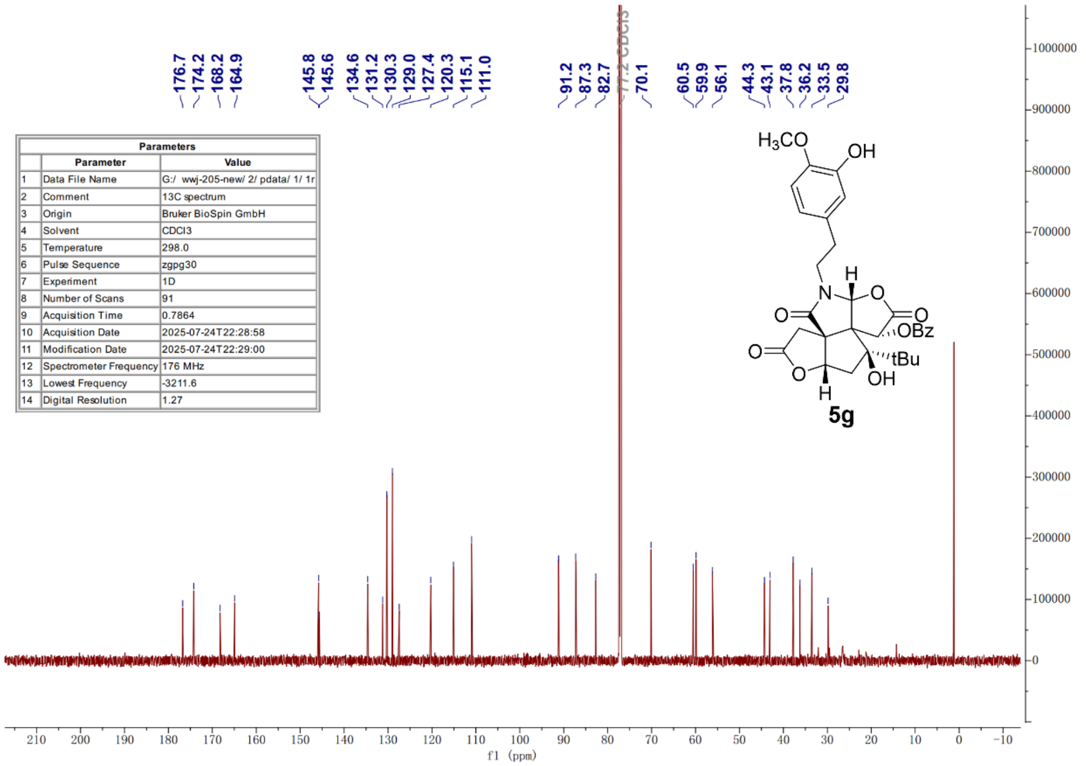

	Parameter	Value
1	Data File Name	G:/ wwj-205-new/ 2/ pdata/ 1/ 1r
2	Comment	13C spectrum
3	Origin	Bruker BioSpin GmbH
4	Solvent	CDCl3
5	Temperature	298.0
6	Pulse Sequence	zgpg30
7	Experiment	1D
8	Number of Scans	91
9	Acquisition Time	0.7864
10	Acquisition Date	2025-07-24T22:28:58
11	Modification Date	2025-07-24T22:29:00
12	Spectrometer Frequency	176 MHz
13	Lowest Frequency	-3211.6
14	Digital Resolution	1.27

(3a*S*,5a*S*,8*R*,9*R*,10a*S*)-9-(*tert*-Butyl)-5-(3,4-dihydroxyphenethyl)-9-hydroxy-2,4,7-trioxo-octahydro-4*H*,9*H*-furo[2,3-*b*]furo[3',2':2,3]cyclopenta[1,2-*c*]pyrrol-8-yl benzoate (5h)

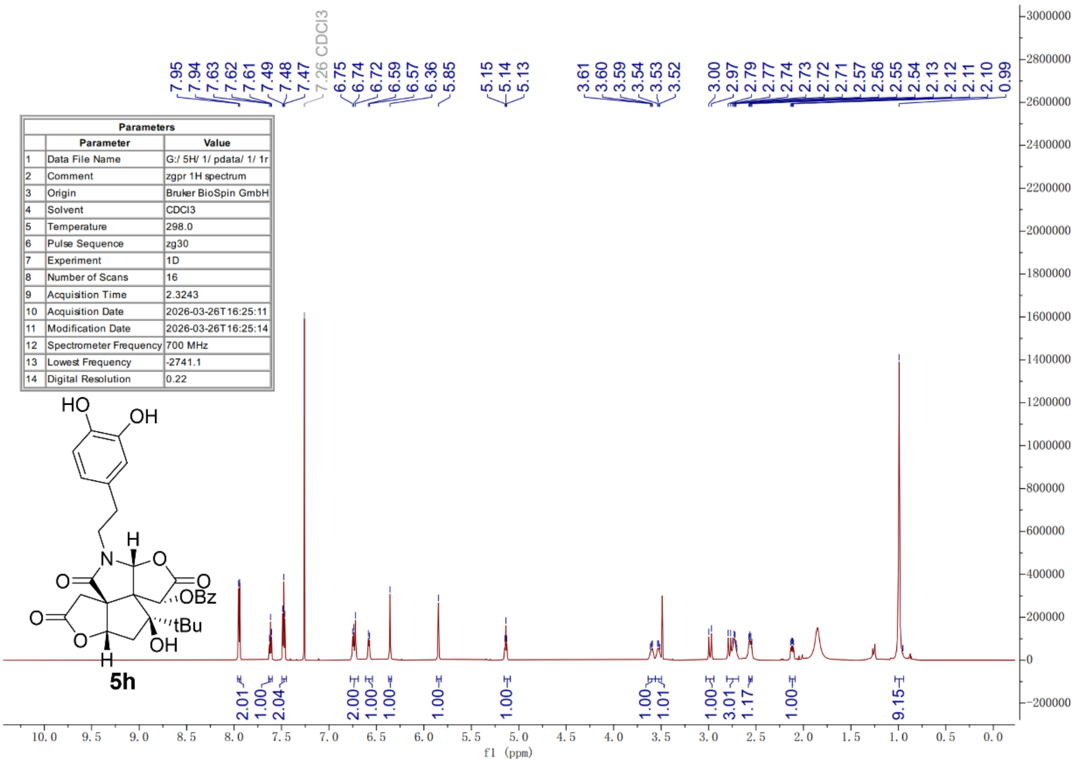

Parameters		
	Parameter	Value
1	Data File Name	G:/ 5H/ 1/ pdata/ 1/ 1r
2	Comment	zgpr 1H spectrum
3	Origin	Bruker BioSpin GmbH
4	Solvent	CDCl3
5	Temperature	298.0
6	Pulse Sequence	zg30
7	Experiment	1D
8	Number of Scans	16
9	Acquisition Time	2.3243
10	Acquisition Date	2026-03-26T16:25:11
11	Modification Date	2026-03-26T16:25:14
12	Spectrometer Frequency	700 MHz
13	Lowest Frequency	-2741.1
14	Digital Resolution	0.22

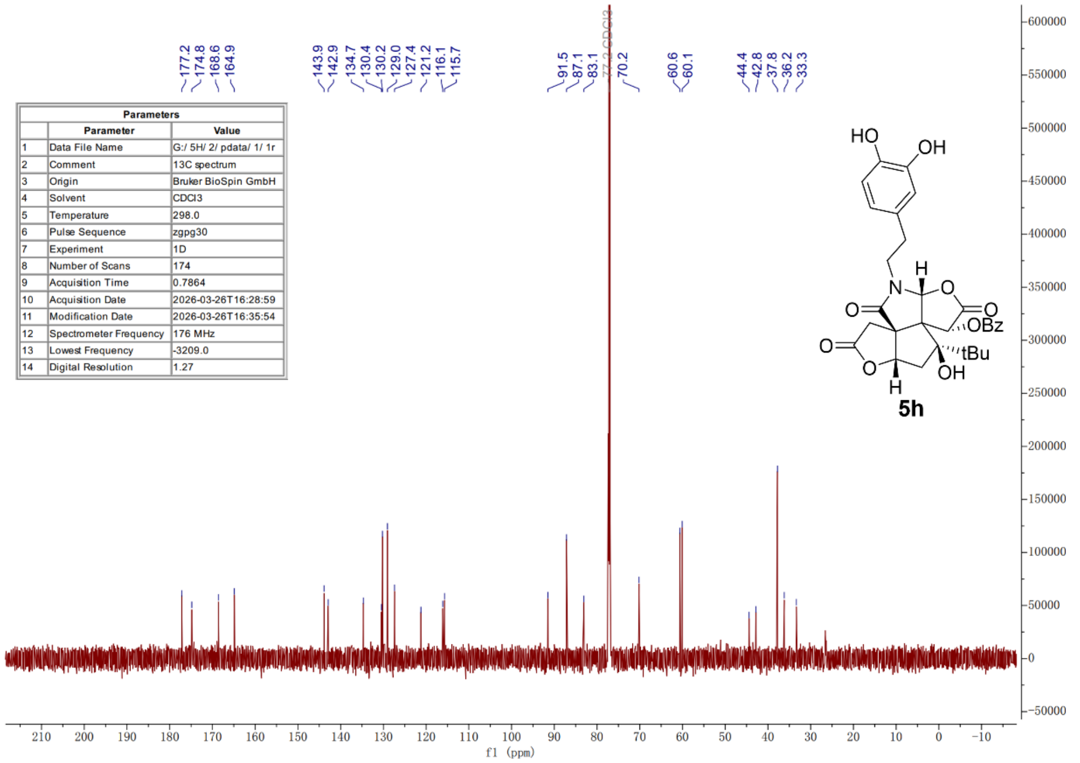

Parameters		
	Parameter	Value
1	Data File Name	G:/ 5H/ 2/ pdata/ 1/ 1r
2	Comment	13C spectrum
3	Origin	Bruker BioSpin GmbH
4	Solvent	CDCl3
5	Temperature	298.0
6	Pulse Sequence	zgpg30
7	Experiment	1D
8	Number of Scans	174
9	Acquisition Time	0.7864
10	Acquisition Date	2026-03-26T16:28:59
11	Modification Date	2026-03-26T16:35:54
12	Spectrometer Frequency	176 MHz
13	Lowest Frequency	-3209.0
14	Digital Resolution	1.27

(3a*S*,5a*R*,8*R*,9*R*,10a*S*)-5-(2-(benzo[*d*][1,3]dioxol-5-yl)ethyl)-9-(*tert*-butyl)-9-hydroxy-2,4,7-trioxooctahydro-4*H*,9*H*-furo[2,3-*b*]furo[3',2':2,3]cyclopenta[1,2-*c*]pyrrol-8-yl benzoate (5i)

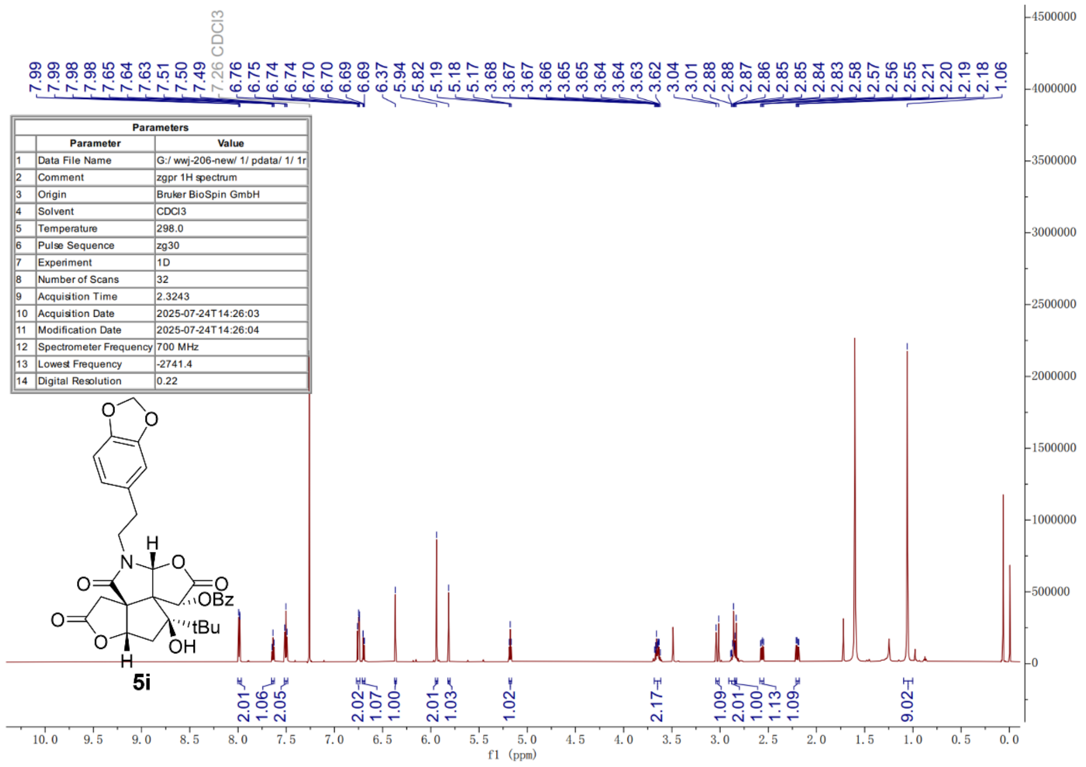

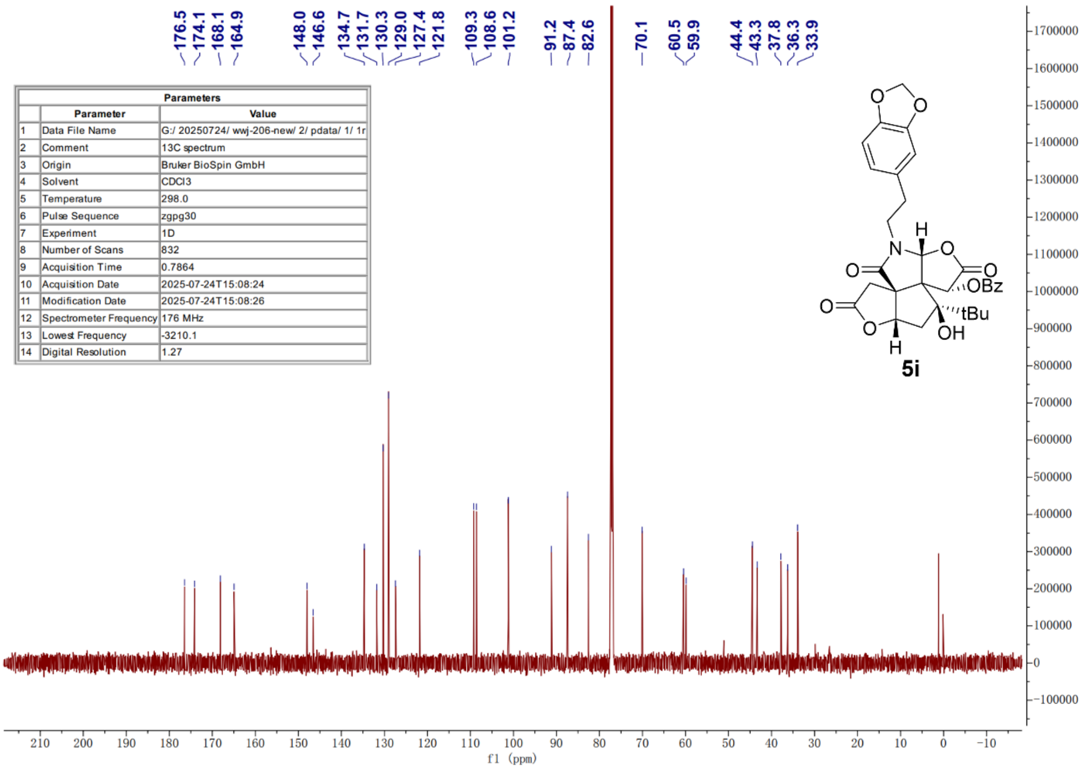

(3a*S*,5a*R*,8*R*,9*R*,10a*S*)-9-(*tert*-butyl)-9-hydroxy-5-(4-nitrophenethyl)-2,4,7-trioxooctahydro-4*H*,9*H*-furo[2,3-*b*]furo[3',2':2,3]cyclopenta[1,2-*c*]pyrrol-8-yl benzoate (5j)

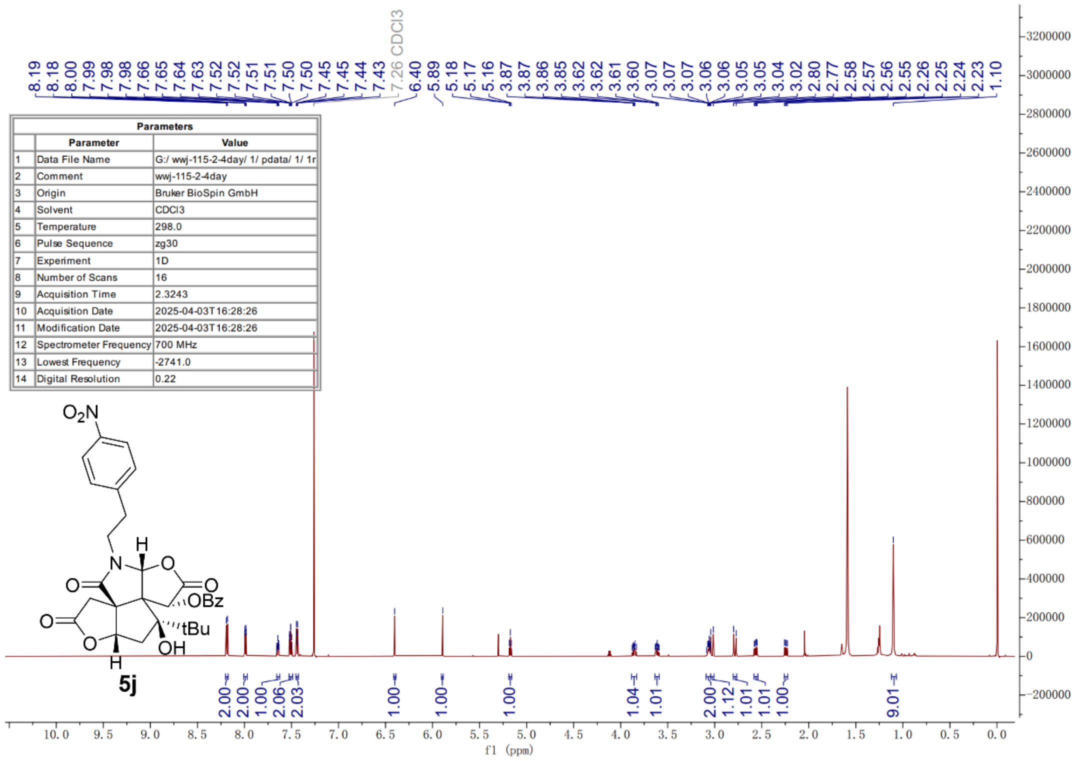

	Parameter	Value
1	Data File Name	G:/ wwj-115-2-4day/ 1/ pdata/ 1/ 1r
2	Comment	wwj-115-2-4day
3	Origin	Bruker BioSpin GmbH
4	Solvent	CDCl3
5	Temperature	298.0
6	Pulse Sequence	zg30
7	Experiment	1D
8	Number of Scans	16
9	Acquisition Time	2.3243
10	Acquisition Date	2025-04-03T16:28:26
11	Modification Date	2025-04-03T16:28:26
12	Spectrometer Frequency	700 MHz
13	Lowest Frequency	-2741.0
14	Digital Resolution	0.22

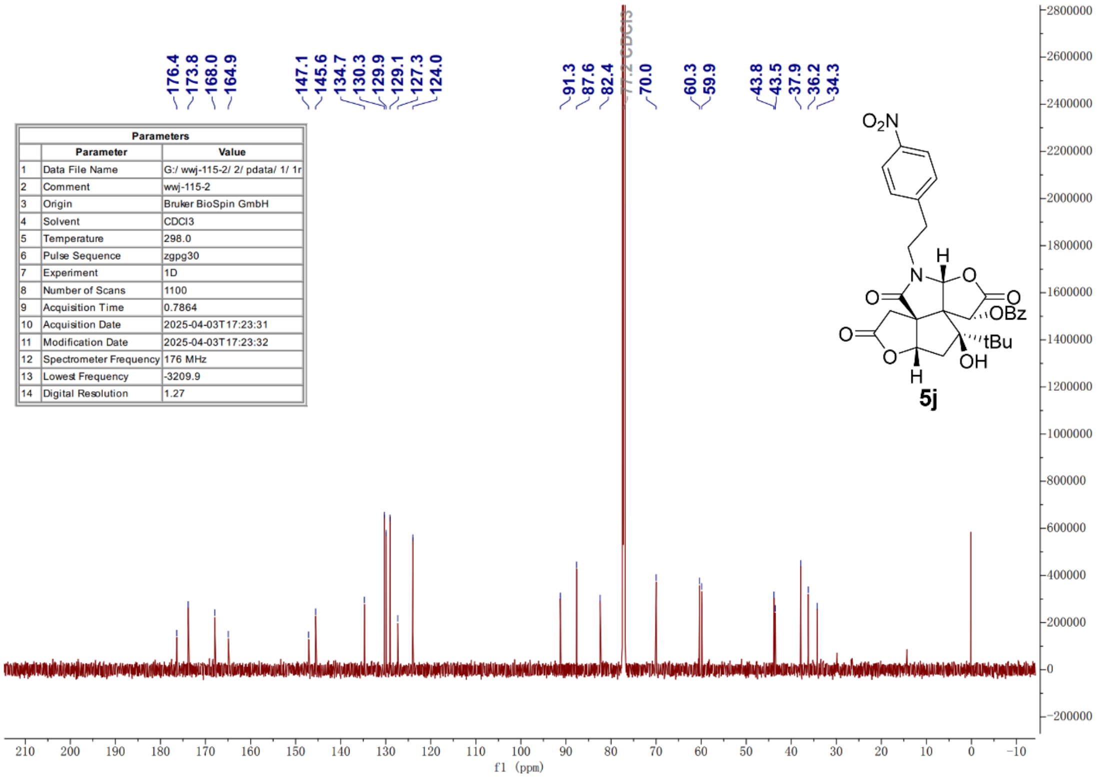

	Parameter	Value
1	Data File Name	G:/ wwj-115-2/ 2/ pdata/ 1/ 1r
2	Comment	wwj-115-2
3	Origin	Bruker BioSpin GmbH
4	Solvent	CDCl3
5	Temperature	298.0
6	Pulse Sequence	zgpg30
7	Experiment	1D
8	Number of Scans	1100
9	Acquisition Time	0.7864
10	Acquisition Date	2025-04-03T17:23:31
11	Modification Date	2025-04-03T17:23:32
12	Spectrometer Frequency	176 MHz
13	Lowest Frequency	-3209.9
14	Digital Resolution	1.27

(3a*S*,5a*R*,8*R*,8a*S*,9*R*,10a*S*)-9-(*tert*-butyl)-9-hydroxy-5-(4-hydroxyphenethyl)-2,4,7-trioxooctahydro-4*H*,9*H*-furo[2,3-*b*]furo[3',2':2,3]cyclopenta[1,2-*c*]pyrrol-8-yl benzoate (5k)

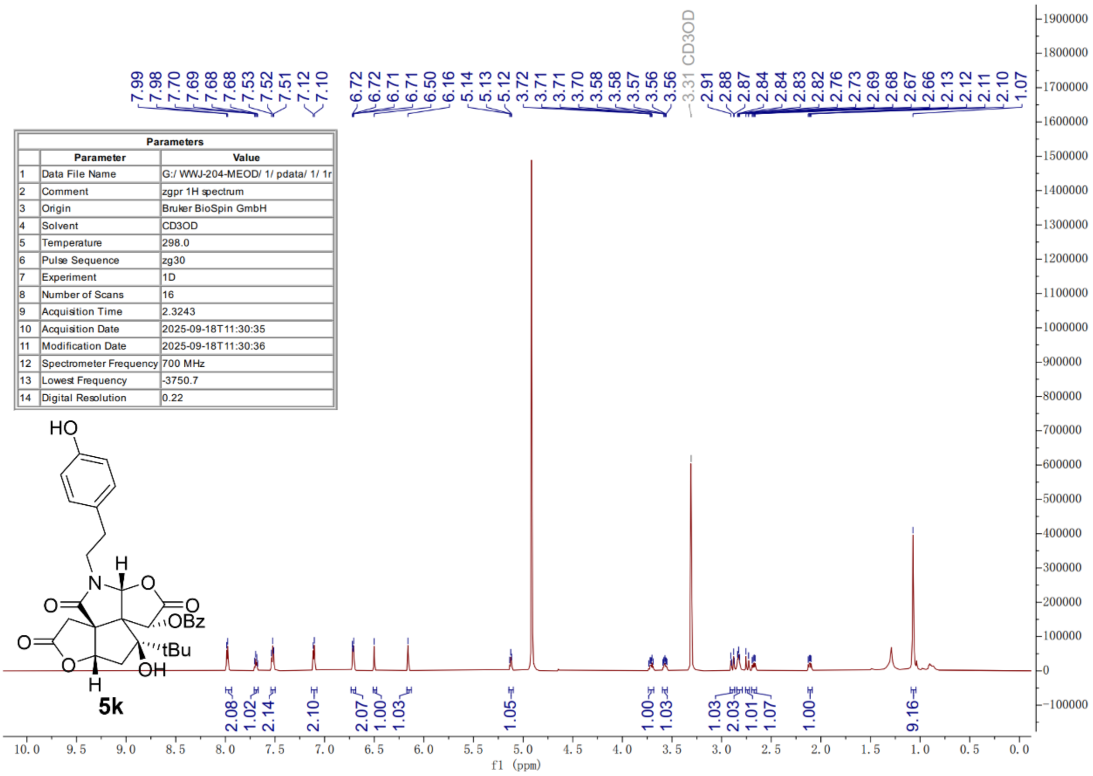

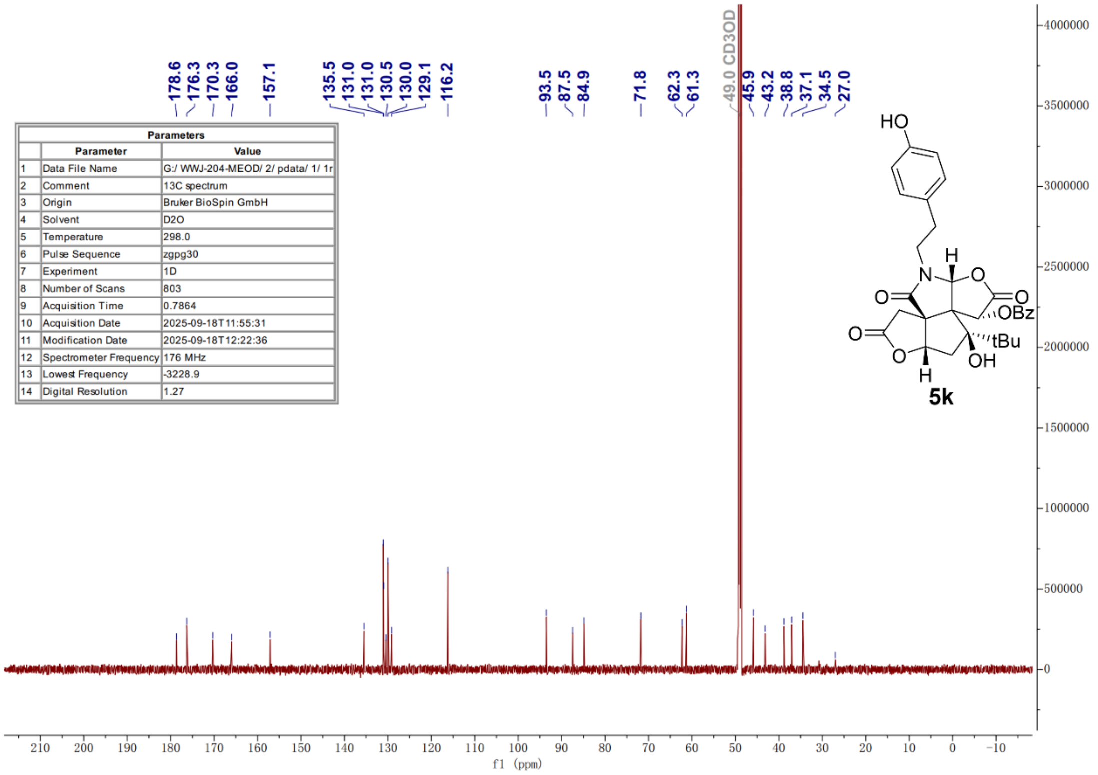

Supplement: Supplementary file 1 [file ol6c01262_si_001.pdf]
